# Synthesis and Antimicrobial Activity of Canthin-6-One Alkaloids

**DOI:** 10.3390/molecules30071546

**Published:** 2025-03-31

**Authors:** Xubing Qi, Yogini Jaiswal, Xinrong Xie, Yu Fan, Rongping Wu, Shaoyang Su, Yifu Guan, Leonard Williams, Xun Song

**Affiliations:** 1 Key Laboratory of Chemistry and Engineering of Forest Products (State Ethnic Affairs Commission), Guangxi Collaborative Innovation Center for Chemistry and Engineering of Forest Products, School of Chemistry and Chemical Engineering, Guangxi Minzu University, Nanning 530006, China; 18789419455@163.com (X.Q.); 20199030@gxmzu.edu.cn (X.X.); ffyu012@163.com (Y.F.); 18169417717@163.com (R.W.); 2 Center for Excellence in Post-Harvest Technologies, North Carolina Agricultural and Technical State University, The North Carolina Research Campus, Kannapolis, NC 28081, USA; ysjaiswa@ncat.edu; 3 Department of Applied Chemistry Teaching and Research, Guangxi Vocational University of Agriculture, Nanning 530007, China; susy@gxnzd.edu.cn; 4 College of Pharmacy, Shenzhen Technology University, Shenzhen 518118, China

**Keywords:** canthin-6-one alkaloids, synthesis, derivatives, antimicrobial activity

## Abstract

Canthin-6-one alkaloids have consistently attracted the interest of medicinal chemists due to their wide range of promising bioactivities, including antitumor, antifungal, antibacterial, and antiviral properties. However, their low natural abundance in plants has constrained the further exploration of their potential bioactivities. This study reports a comprehensive synthesis of canthin-6-one alkaloids, utilizing key Suzuki coupling and Cu-catalyzed amidation reactions to construct their core scaffold. Derivatives were synthesized with Koenig–Knorr glycosylation for the further modification of synthetic canthin-6-ones. The antimicrobial activities of the synthesized compounds were evaluated against *C. albicans*, *C. neoformans*, *S. aureus* and *E. coli* using the micro-dilution method. In total, 17 compounds were synthesized, including nine canthin-6-ones. Notably, alkaloids **4**, **5**, **7** and **12**-**13** were prepared for the first time, along with 8 new derivatives. Their structures were confirmed by NMR and MS analyses. At 50 µg/mL, the alkaloids **1**-**4** and **9** exhibited antimicrobial properties against *C. albicans*, *C. neoformans* and *S. aureus*. The antimicrobial activity of alkaloids **2**, **4**-**5** and **12**-**13** against these four microbial human pathogens is reported here for the first time. Overall, this research not only advances our understanding of canthin-6-one alkaloid synthesis, but also provides a foundation for developing novel compounds with pharmaceutical properties.

## 1. Introduction

**Figure 1:**
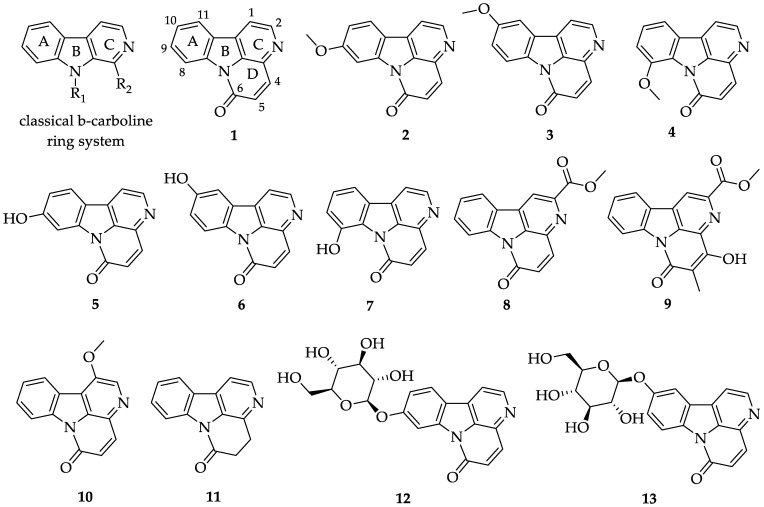
Structure of natural canthin-6-one alkaloids.

In recent decades, there has been a renewed focus on natural products for innovative drug discovery [1]. Alkaloids, a class of nitrogen-containing organic compounds found in plants, fungi, bacteria and other organisms, have been used therapeutically for over 3000 years [2,3]. Many alkaloids, including paclitaxel, vincristine, atropine, pilocarpine, quinine and vincamine, are used clinically [4]. The canthin-6-one (or canthinone) alkaloids are a subclass of β-carboline alkaloids with an additional D-ring (Figure 1). Canthin-6-one **1** was first isolated in 1952 by Haynes from the Australian plant species *Pentaceras australis* (F.Muell.) Benth. (Rutaceae) [5]. Since then, nearly 90 canthin-6-one alkaloids have been isolated from over fifty species (the structure of some canthin-6-one alkaloids is shown in Figure 1). These alkaloids are predominantly found in the root, bark and leaves of Rutaceae and Simaroubaceae, with smaller quantities present in their heartwood. Additionally, genera from Malvaceae, Amaranthaceae, Zygophyllaceae, Caryophylaceae and Leitneriaceae also contain canthin-6-one alkaloids [6].

Canthin-6-one alkaloids and their derivatives demonstrate a wide range of biological activities, including antiviral [7,8,9], antitumor [10,11,12], antiparasitic [13,14,15], anti-inflammatory [16,17], antibacterial [18,19,20] and antifungal [21,22]. Notably, canthine-6-one alkaloids and their derivatives have gained significant attention due to their antimicrobial potencies. For example, O’Donnell et al. isolated two canthin-6-ones, with minimum inhibitory concentrations (MIC) ranging from 8 to 32 µg/mL against a panel of fast-growing *Mycobacterium* species and 8 to 64 µg/mL against multidrug-resistant and methicillin-resistant strains of *Staphylococcus aureus* [20]. Wang et al. synthesized a novel series of hydrophilic derivatives of canthin-6-one with a peak MIC of 0.98 µg/mL against *S. aureus* [18,23,24]. Li et al. synthesized twenty-two new 3-*N*-benzylated 10-methoxy canthin-6-ones through a quaternization reaction. The in vitro antibacterial activity against *R. solanacearum*, *P. syringae* and *B. cereus* revealed that two compounds demonstrated 8-fold superior activities (MIC = 3.91 μg/mL) against the agricultural pathogenic bacteria *R. solanacearum* and *P. syringae*, compared to agrochemical streptomycin sulfate. They showed good antibacterial activity against *B. cereus*, along with favorable “drug-like” properties, low cytotoxicity and no adverse effects on seed germination [25]. Dai et al. synthesized C2- and C5-modified, D-ring-rearranged canthin-6-ones, along with C10 and truncated canthin-6-ones in two separate studies, to evaluate their antibacterial activity and SARs [26,27]. The C_2_-substituted and D-ring-rearranged compound exhibited broad-spectrum antibacterial activity, effective against all the tested bacteria, including *S. aureus*, *B. subtilis* (MIC = 12.5 nmol/mL), *MRSA*, *B. cereus* and *R. solanacearum* (MIC = 25 nmol/mL), but not *E. coli* (MIC > 200 nmol/mL) [26]. Additionally, the C-ring-truncated canthin-6-one showed a broad-spectrum antibacterial effect, with an eight-fold superior activity against *S. aureus*. It showed a two-fold superiority against *Ralstonia solanacearum* compared to fosfomycin sodium and propineb, with an MIC of 2 μg/mL.

**Figure 2:**
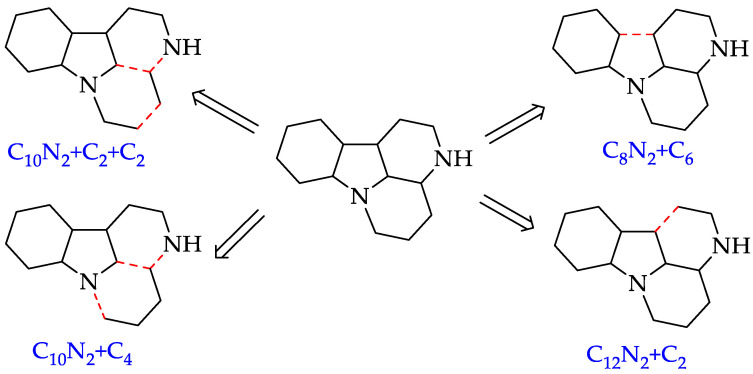
Different synthetic strategies to achieve canthin-6-one scaffolds.

Due to the promising applications of canthin-6-one alkaloids, numerous reports have been published on their synthesis. In 2022, Cebrián-Torrejón’s group evaluated a series of synthesis strategies for canthin-6-one scaffolds (Figure 2) [6]. Most of these strategies employed tryptamine or indole-based precursors, primarily achieved through reductive amination, Pictet–Spengler reactions, amidation reactions, catalytic hydrogen transfer and catalytic oxidation reactions. However, it is found that some of the original synthetic approaches do not rely on tryptamine or its derivatives. These methods include the following: the C_10_N_2_ + C_2_ + C_2_ strategy, which was used to synthesize compounds **8** and **9**; the C_10_N_2_ + C_4_ strategy, which was used to synthesize compounds **1**, **8** and **10**; the C_8_N_2_ + C_6_ strategy, which was used to synthesize compound **1**; and the C_12_N_2_ + C_2_ strategy, which led to the synthesis of canthine alkaloid **11**. Among these strategies, the C_8_N_2_ + C_6_ strategy is the most prominent, with canthin-6-one **1** synthesized in three steps, achieving a total yield of 86.4% [6]. The strategies mentioned above were used to structure and guide our synthesis approach in the present study.

So far, there are no reports on the synthesis of some canthin-6-one alkaloids, including canthin-6-ones **4**, **5**, **7**, **12** and **13**. There is limited literature available on the bioactivities of canthin-6-one alkaloids and their potential antimicrobial activities. Herein, we report the synthesis and evaluation of the antimicrobial activity of canthin-6-one alkaloids, along with a novel series of canthin-6-one alkaloids’ derivatives for the first time.

## 2. Results and Discussion

### 2.1. Synthesis of Canthin-6-Ones ***1***-***4***

**Figure 3:**
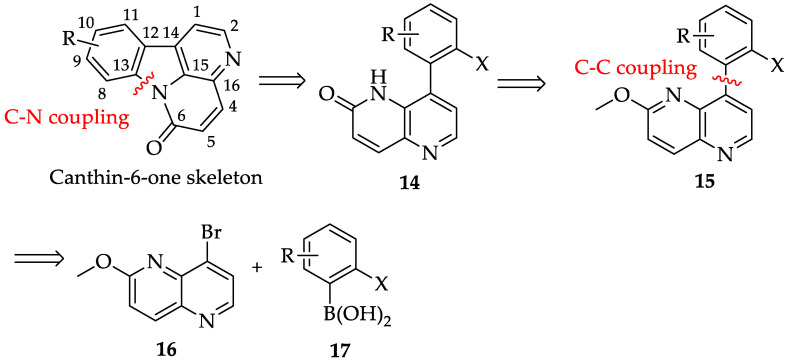
Retrosynthetic analysis of canthin-6-one skeleton.

A variety of synthetic strategies for constructing the canthin-6-one skeleton have been reported [6]. For instance, the canthin-6-one skeleton can be constructed through the cleavage of either the C1-C14 and C15-C16 bonds or the C6-N and C15-C16 bonds [21,28]. By comparing different synthetic stragegies, the retrosynthetic analysis of canthin-6-one skeleton in this study was based on reported synthetic routes for canthin-6-one alkaloids [29], and is illustrated in Figure 3. This approach primarily focused on constructing ring B. Intermediate **14** was generated by cleaving the amide bond at C13, while building blocks **16** and **17** were derived from intermediate **15** through the cleavage of the C12-C14 bond. These two bond formations were achieved via an intermolecular C-C coupling, followed by an intramolecular C-N coupling. Notably, incorporating ring A in the final stage of synthesis provided structural flexibility, allowing for modification at the C8-C11 positions.

**Scheme 1:**
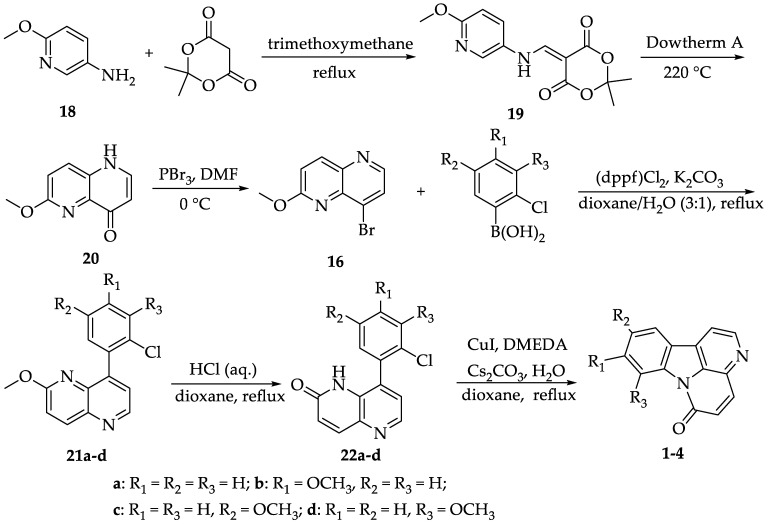
Synthesis of canthin-6-one alkaloids **1**-**4.**

To synthesize canthin-6-ones **1**-**4**, the key intermediate **16** was initially prepared via a three-step reaction sequence, starting with commercially available 6-methoxypyridin-3-amine **18** as the precursor (Scheme 1), as described in the reference [30]. Briefly, the first step involved the condensation of **18** with Meldrum’s acid and trimethoxymethane. This domino sequence included an addition-elimination reaction, resulting in the enamine intermediate **19** with a yield of 74.1%. In the next step, thermal Friedel–Crafts cyclization with decarboxylation was used to synthesize **20**, and it had a good yield of 73.3%. Finally, a straightforward bromination of **20** with PBr_3_ at 0 °C yielded the key intermediate **16**, which had a yield of 73.3% after purification by silica gel column chromatography. Compound **16** was obtained in three steps with an overall yield of 44.5%, as reported in the reference [30]. Using the same method, we achieved an overall yield of 39.8%. The reduction in overall yield is attributed to the purification of our final product by column chromatography.

Subsequently, compound **21** was prepared from intermediate **16** and 2-chlorophenylboronic acid vis Suzuki–Miyaura coupling, achieving an excellent yield (89.9–99.5%) when Pd(dppf)Cl_2_ was used together with K_2_CO_3_ in a dioxane/H_2_O solvent system. Refluxing **21** with aqueous HCl in dioxane resulted in the formation of naphthyridone **22**. During this step, some of product **22** may have formed a salt under acidic conditions, which led to a significant product loss when a direct extraction of the aqueous phase was carried out with CH_2_Cl_2_. To address this, we adjusted the pH to 7-8 using solid Na_2_CO_3_ before extraction, which resulted in a high-yield recovery of product **22** (82.2-97.7%). The NMR data for intermediates **21a**, **21b**, **22a** and **22b** were consistent with those of the corresponding intermediates reported in reference [29]. Intermediates **21c** and **21d** were obtained for the first time, along with **22c** and **22d**, and their structures were confirmed by NMR spectroscopy. The final C-N coupling was carried out under Buchwald’s conditions (CuI, DMEDA as the ligand and H_2_O in dioxane at reflux), finishing in 1.5-2.0 h with a yield of 90.9-98.9%. Therefore, it is evident that the approach successfully synthesized canthin-6-ones **1**-**4** in three steps from commercially available starting material **16** with high yields (Scheme 1) [29].

### 2.2. Synthesis of Compounds ***27***-***28***

**Scheme 2:**
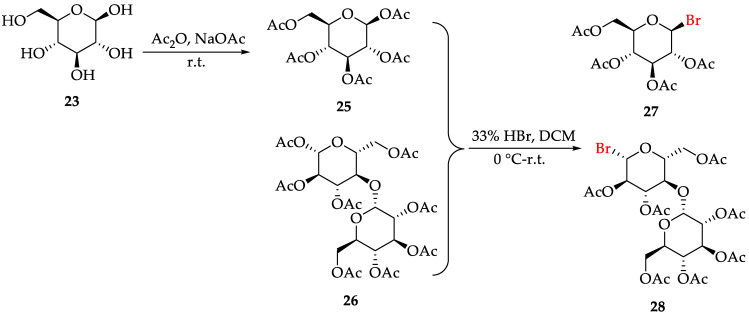
Preparation of compounds **27** and **28.**

The synthesis of glycoside alkaloids **12** and **13** was planned using Koenig–Knorr glycosylation. Therefore, 2,3,4,6-tetra-O-acetyl-β-D-glucopyranosyl bromide **27** needs to be synthesized first. β-D-glucose **23** was esterified followed by bromination to yield **27** [31], with a total yield of 49.0%, as described in Scheme 2. For the synthesis of the analogs of glycoside alkaloids **12**, acetobromo-β-D-maltose **28** was obtained using the same synthetic method [32], with commercially available aceto-β-D-maltose **26** as raw material (yield: 76.2%).

### 2.3. Synthesis of Compounds ***5***-***7***, ***12***-***13*** and ***30***-***31***

**Scheme 3:**
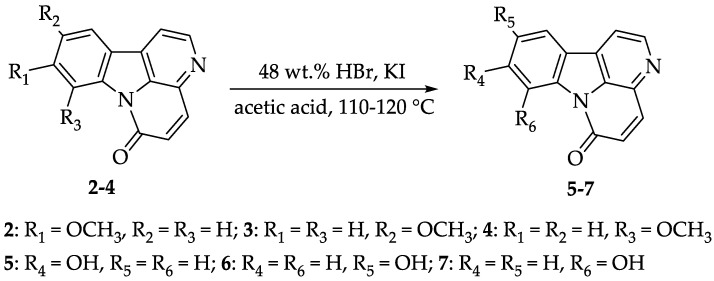
Synthesis of alkaloids **5**-**7.**

Zhao et al. reported that deprotecting the methoxy group of 10-methoxycanthin-6-one **3** using boron tribromide can yield 10-hydroxycanthin-6-one **6** [24]. However, this reaction requires a temperature of −78 °C, and the yield is moderate. To make the operation more convenient and achieve a higher yield, we explored a strategy for the methoxy-to-hydroxyl transformation. A widely used method for deprotecting the methoxy group involves 48 wt.% HBr. We successfully applied this method to synthesize **5**-**7**, and compounds **5** and **7** were obtained for the first time (Scheme 3). Using potassium iodide as a catalyst and adding a controlled amount of acetic acid under heating, the yields of alkaloids **5**-**7** ranged from 82.9 to 99.1%. An HPLC purity test was conducted for natural product **5** (Figure S1), confirming a purity of over 98%.

**Scheme 4:**
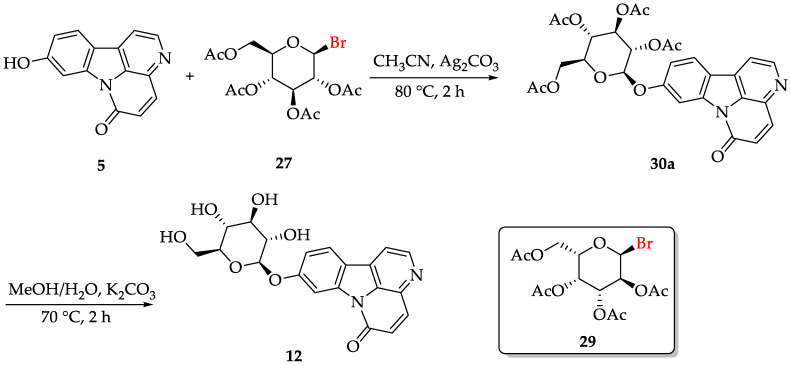
Synthesis of glucopyranoside alkaloids **12**.

With **5**-**7** in hand, glucopyranoside alkaloids and their derivatives were possible to synthesize (the names of derivatives are shown in Table 1). When canthin-6-one **5** reacted with bromide **27** in the presence of silver carbonate, Koenig–Knorr glycosylation occurred, yielding **30a** with an 83.5% yield. Subsequent hydrolysis with potassium carbonate led to the synthesis of glucopyranoside alkaloid **12** (with a yield of 86.6%) (Scheme 4). When the starting material is canthin-6-one **6**, the same method yields glucopyranoside alkaloid **13**. Glucopyranoside alkaloids **12** and **13** were synthesized in six steps, with overall yields of 61.5% and 41.2%, respectively, starting from commercially available starting material **16**. Similarly, when canthin-6-one **5** and **6** and commercially available available 2,3,4,6-Tetra-O-acetyl-α-D-galactopyranosyl bromide **29** or canthin-6-one **6** and bromide **28** are used as the starting material, analogs **31a**-**31c** can be obtained with good yields (yield range: 75.6–94.1%). Unfortunately, compound **7** did not react with compound **29**, likely due to the steric hindrance of the carbonyl group, which was relatively large and positioned close to the C8 site, preventing the glycosylation reaction from occurring.

### 2.4. Comparison of NMR Data

By comparing the NMR spectroscopic data, it was confirmed that the synthesized compounds **1**-**7** and **12**-**13** are natural canthin-6-ones **1**-**7** and **12**-**13**. For example, the ^13^C NMR chemical shifts of natural and synthetic compound **12** are presented in Table 2. The ^13^C NMR of canthin-6-one **12** closely matched that of the synthetic compound **12**, with the largest significant discrepancies of 0.2 ppm observed for some carbon atoms. This strongly supports the identification of synthetic compound **12** as the natural product of canthin-6-one **12**, as reported by Yamasaki et al. [33].

### 2.5. Initial Screening for Antimicrobial Activity

The synthesized compounds were initially evaluated against four microbial human pathogen strains (*C. albicans*, *C. neoformans*, *S. aureus* and *E. coli*) using the micro-dilution method. The results are summarized in Table 3.

As shown in Table 3, canthin-6-ones **1** and **7** demonstrated inhibition rates exceeding 80% against *C. albicans*, while canthin-6-ones **1**, **2**, **4** and **7** showed more than 70% inhibition against *C. neoformans* at 50 µg/mL, comparable to the positive control drug Ketoconazole. Among them, canthin-6-one **1** displayed the highest activity, with inhibition rates of 95.7% against *C. albicans* and 96.9% against *C. neoformans*. Canthin-6-ones **1**-**3** and **7** showed activity against *S. aureus*, with inhibition rates ranging from 88.1% to 99.8%. However, all tested compounds exhibited inhibition rates below 50% against *E. coli* at the same concentration, indicating a lack of antibacterial activity against this strain. Additionally, all derivatives showed no inhibitory effect against the four microbial phytopathogens.

The antimicrobial activity of canthin-6-ones **2**, **4**-**5** and **12**-**13** against these four microbial human pathogens was reported here for the first time, along with the antimicrobial activity of canthin-6-ones **2** and **4**-**5** against *C. albicans*, *C. neoformans* and *E. coli*.

## 3. Materials and Methods

### 3.1. General Chemical Procedures

All chemicals were purchased from commercial suppliers and used without further purification. All reagents and solvents were reagent or HPLC grade. ^1^H and ^13^C NMR spectra were recorded on a Bruker spectrometer at 400 MHz (Billerica, MA, USA) in the indicated solvents. Chemical shifts (δ) were expressed in ppm with reference to the solvent signals (CDCl_3_, ^1^H: 7.26 ppm; ^13^C: 77.00 ppm), and coupling constants (J) are reported in Hz. Standard acronyms representing multiplicity are used as follows: brs = broad singlet, s = singlet, d = doublet, t = triplet and m = multiplet. All NMR experiments were obtained using standard pulse sequences supplied by the vendor. Thin-layer chromatography (TLC) was performed on thin-layer chromatography GF 254 (10–40 μm) silica gel plates. Column chromatography was carried out on silica gel (200–300 mesh, Qingdao Marine Chemical Factory, Qingdao, China). LC-MS instrument was purchased from Agilent Technology (Hong Kong, China) Co., Ltd. (Agilent 1260 + 6125).

### 3.2. Synthesis of Target Compounds

#### 3.2.1. Synthesis of Compound **19** [30]

A mixture of 2,2-dimethyl-1,3-dioxane-4,6-dione (2.9 g, 20.0 mmol) and trimethoxymethane (19.1 g, 180.0 mmol) in ethanol (6 mL) was heated under reflux for 2 h. Afterwards, 6-methoxypyridin-3-amine (2.5 g, 20.0 mmol) was added, and the resulting solution was stirred under reflux for 12 additional hours. Upon cooling to room temperature, the mixture was diluted with hexane (30 mL) and stirred in an ice bath for 5–10 min, resulting in the formation of a significant amount of precipitate. The precipitate was filtered and washed with hexane, yielding the product (74.1% yield) as a purple solid. ^1^H NMR (400 MHz, CDCl_3_) δ (ppm): 11.17 (d, *J* = 14.20 Hz, 1H), 8.48 (d, *J* = 14.24 Hz, 1H), 8.11 (d, *J* = 2.88 Hz, 1H), 7.52 (dd, *J* = 8.92, 2.92 Hz, 1H), 6.82 (d, *J* = 8.92 Hz, 1H), 3.94 (s, 3H), 1.75 (s, 6H).

#### 3.2.2. Synthesis of Compound **20** [30]

Dowtherm A (approximately 3:7 diphenyl/diphenyl oxide, 40 mL) was added to a single-neck round-bottom flask equipped with a thermocouple and a funnel, and the solvent was heated to 220 °C. To the heated Dowtherm A, compound **19** (4.1 g, 14.7 mmol) was added over a 30 min period, maintaining the temperature at approximately 220 °C. The funnel was then rinsed with 10 mL of Dowtherm A. The reaction mixture was heated for an additional 10 min, after which the heat source was removed. The resulting suspension was allowed to cool to room temperature and treated with n-hexane (50 mL). The mixture was stirred in an ice bath for 5–10 min, and the solid product was filtered, washed with hexane (50 mL) and dried in vacuo to yield 1.9 g of compound **20** as a tan solid (73.3%). The crude product was used directly for the next step of the reaction. ^1^H NMR (400 MHz, DMSO-*d_6_*) δ (ppm): 11.87 (br s, 1H), 7.95 (br s, 2H), 7.16 (d, *J* = 8.96 Hz, 1H), 6.22 (br s, 1H), 3.93 (s, 3H).

#### 3.2.3. Synthesis of Compound **16** [30]

PBr_3_ (1.1 mL) was added to a solution of compound **20** (1.8 g, 10.2 mmol) in DMF (2.1 mL) under a nitrogen atmosphere at 0 °C. The mixture was stirred at 0 °C for 1 h, then quenched with H_2_O (107 mL) and 6 N NaOH (0.28 mL), followed by extraction with ethyl acetate (3 × 100 mL). The combined organic layers were washed with brine (50 mL) and dried over anhydrous Na_2_SO_4_, and the solvent was removed under vacuum. The residue was purified by flash column chromatography to obtain a white solid product with 73.3% yield. ^1^H NMR (400 MHz, DMSO-*d_6_*) δ (ppm): 8.59 (d, *J* = 4.76 Hz, 1H), 8.29 (d, *J* = 8.96 Hz, 1H), 8.07 (d, *J* = 4.68 Hz, 1H), 7.33 (d, *J* = 9.00 Hz, 1H), 4.06 (s, 3H).

#### 3.2.4. Synthesis of Compounds **21**

To a stirred solution of compound **16** (0.3 g, 1.3 mmol), a 3:1 mixture of 1,4-dioxane/H_2_O (2.5 mL), K_2_CO_3_ (0.3 g, 2.5 mmol), Pd(dppf)Cl_2_ (36.9 mg, 0.05 mmol) and benzene boric acid (1.9 mmol) was added. The reaction mixture was warmed to 100–105 °C and stirred for 2–3 h. Upon completion, the mixture was diluted with H_2_O (20 mL) and extracted with CH_2_Cl_2_ (3 × 20 mL). The combined organic layers were washed with brine (40 mL) and dried over anhydrous Na_2_SO_4_, and the solvent was removed under vacuum. The residue was purified by flash column chromatography to obtain the final product.

Compound **21a**: colorless transparent oil, yield: 89.9%. ^1^H NMR (400 MHz, CDCl_3_) δ (ppm): 8.83 (d, *J* = 4.44 Hz, 1H), 8.25 (d, *J* = 9.04 Hz, 1H), 7.55–7.50 (m, 2H), 7.44–7.40 (m, 1H), 7.39–7.33 (m, 2H), 7.12 (d, *J* = 9.04 Hz, 1H), 3.81 (s, 3H). ^13^C NMR (100 MHz, CDCl_3_) δ (ppm): 161.9, 147.5, 144.7, 142.3, 140.2, 140.0, 136.6, 133.7, 131.9, 129.5, 129.4, 126.3, 125.0, 116.7, 53.7.

Compound **21b**: white solid, yield: 90.1%. ^1^H NMR (400 MHz, DMSO-*d_6_*) δ (ppm): 8.84 (d, *J* = 4.52 Hz, 1H), 8.32 (d, *J* = 9.00 Hz, 1H), 7.62 (d, *J* = 4.44 Hz, 1H), 7.45 (d, *J* = 8.48 Hz, 1H), 7.26 (d, *J* = 9.00 Hz, 1H), 7.20 (d, *J* = 2.52 Hz, 1H), 7.05 (dd, *J* = 8.60, 2.56 Hz, 1H), 3.85 (s, 3H), 3.77 (s, 3H). ^13^C NMR (100 MHz, DMSO-*d_6_*) δ (ppm): 161.2, 159.8, 147.7, 143.4, 141.7, 140.4, 139.3, 133.2, 132.8, 127.9, 125.4, 116.4, 114.5, 112.8, 55.7, 53.2.

Compound **21c**: yellow transparent oil, yield: 98. 9%. ^1^H NMR (400 MHz, DMSO-*d_6_*) δ (ppm): 8.86 (d, *J* = 4.44 Hz, 1H), 8.33 (d, *J* = 9.00 Hz, 1H), 7.65 (d, *J* = 4.44 Hz, 1H), 7.50 (d, *J* = 8.28 Hz, 1H), 7.27 (d, *J* = 9.04 Hz, 1H), 7.11–7.03 (m, 2H), 3.79 (s, 3H), 3.76 (s, 3H). ^13^C NMR (100 MHz, DMSO-*d_6_*) δ (ppm): 161.3, 157.6, 147.7, 143.6, 141.6, 140.4, 139.0, 136.8, 129.9, 125.1, 123.8, 117.0, 116.5, 115.6, 55.6, 53.2.

Compound **21d**: yellow transparent oil, yield: 99.0%. ^1^H NMR (400 MHz, DMSO-*d_6_*) δ (ppm): 8.85 (d, *J* = 4.40 Hz, 1H), 8.33 (d, *J* = 9.04 Hz, 1H), 7.60 (d, *J* = 4.40 Hz, 1H), 7.42 (t, *J* = 8.04 Hz, 1H), 7.29–7.22 (m, 2H), 7.05 (dd, *J* = 7.64, 1.36 Hz, 1H), 3.92 (s, 3H), 3.72 (s, 3H). ^13^C NMR (100 MHz, DMSO-*d_6_*) δ (ppm): 161.3, 154.7, 147.7, 143.8, 141.6, 140.4, 139.1, 137.4, 127.2, 125.0, 123.3, 120.8, 116.5, 112.2, 56.3, 53.1.

#### 3.2.5. Synthesis of Compounds **22**

To a stirred solution of compound **21** (1.5 mmol) in 1,4-dioxane (7.5 mL), a 1:1 mixture of HCl and H_2_O (10.5 mL) was added. The reaction mixture was heated to 100–105 °C and stirred for 2–3 h. Upon completion, H_2_O (30 mL) was added, and the pH was adjusted to 7 by the addition of solid Na_2_CO_3_. The mixture was then extracted with CH_2_Cl_2_ (3 × 30 mL). The combined organic layers were washed with brine (30 mL) and dried over anhydrous Na_2_SO_4_, and the solvent was removed under vacuum. The crude product was obtained directly for the next step of the reaction.

Compound **22a**: white solid, yield: 97.7%. ^1^H NMR (400 MHz, CDCl_3_) δ (ppm): 8.61 (d, *J* = 4.68 Hz, 1H), 8.45 (br s, 1H), 8.04 (d, *J* = 9.80 Hz, 1H), 7.59 (dd, *J* = 7.84, 1.40 Hz, 1H), 7.53–7.42 (m, 2H), 7.34–7.28 (m, 2H), 6.85 (d, *J* = 9.80 Hz, 1H). ^13^C NMR (100 MHz, CDCl_3_) δ (ppm): 161.7, 145.1, 142.5, 137.9, 133.5, 132.3, 132.1, 131.4, 131.2, 130.8, 128.0, 126.0, 125.5.

Compound **22b**: white solid, yield: 96.3%. ^1^H NMR (400 MHz, DMSO-*d_6_*) δ (ppm): 10.97 (br s, 1H), 8.51 (d, *J* = 4.64 Hz, 1H), 7.99 (d, *J* = 9.72 Hz, 1H), 7.33 (d, *J* = 1.36 Hz, 1H), 7.32 (d, *J* = 5.28 Hz, 1H), 7.20 (d, *J* = 2.48 Hz, 1H), 7.04 (dd, *J* = 8.56, 2.52 Hz, 1H), 6.76 (d, *J* = 9.76 Hz, 1H), 3.85 (s, 3H). ^13^C NMR (100 MHz, DMSO-*d_6_*) δ (ppm): 161.6, 160.6, 144,1, 141.5, 137.2, 133.7, 133.5, 133.3, 132.2, 126.2, 126.0, 125.5, 115.1, 113.9, 55.8.

Compound **22c**: yellow solid, yield: 82.2%. ^1^H NMR (400 MHz, DMSO-*d_6_*) δ (ppm): 10.98 (br s, 1H), 8.53 (d, *J* = 4.64 Hz, 1H), 8.00 (d, *J* = 9.72 Hz, 1H), 7.49 (d, *J* = 8.88 Hz, 1H), 7.37 (d, *J* = 4.60 Hz, 1H), 7.08 (dd, *J* = 8.88, 3.08 Hz, 1H), 7.00 (d, *J* = 3.08 Hz, 1H), 6.78 (d, *J* = 9.76 Hz, 1H), 3.78 (s, 3H). ^13^C NMR (100 MHz, DMSO-*d_6_*) δ (ppm): 161.6, 158.3, 144.1, 141.4, 137.3, 134.1, 133.0, 130.6, 126.1, 125.7, 123.6, 116.8, 116.6, 55.6.

Compound **22d**: pink solid, yield: 96.1%. ^1^H NMR (400 MHz, DMSO-*d_6_*) δ (ppm): 10.97 (br s, 1H), 8.52 (d, *J* = 4.64 Hz, 1H), 8.00 (d, *J* = 9.80 Hz, 1H), 7.41 (t, *J* = 7.96 Hz, 1H), 7.33 (d, *J* = 4.64 Hz, 1H), 7.25 (dd, *J* = 8.40, 1.40 Hz, 1H), 6.95 (dd, *J* = 7.60, 1.40 Hz, 1H), 6.77 (d, *J* = 9.76 Hz, 1H), 3.92 (s, 3H). ^13^C NMR (100 MHz, DMSO-*d_6_*) δ (ppm): 161.6, 155.2, 144.2, 141.5, 137.2, 134.7, 133.9, 133.0, 128.2, 126.1, 125.6, 122.7, 121.0, 113.1, 56.3.

#### 3.2.6. Synthesis of Compounds **1**-**4**

To a stirred solution of compound **22** (1.0 mmol) in 1,4-dioxane (4.0 mL), Cs_2_CO_3_ (651.6 mg, 2.0 mmol), CuI (19.1 mg, 0.1 mmol), DMEDA (17.6 mg) and H_2_O (36.0 mg) were sequentially added. The reaction mixture was heated to 100–105 °C and stirred for 1.5–2 h. Upon completion, the mixture was diluted with H_2_O (20 mL) and extracted with CH_2_Cl_2_ (3 × 20 mL). The combined organic layers were washed with brine (30 mL) and dried over anhydrous Na_2_SO_4_, and the solvent was removed under reduced pressure. The residue was purified by flash column chromatography to afford the title products.

Compound **1**: pale yellow solid, yield: 90.9%. ^1^H NMR (400 MHz, CDCl_3_) δ (ppm): 8.80 (d, *J* = 5.08 Hz, 1H), 8.64 (d, *J* = 8.20 Hz, 1H), 8.08 (d, *J* = 7.72 Hz, 1H), 8.00 (d, *J* = 9.80 Hz, 1H), 7.93 (d, *J* = 4.96 Hz, 1H), 7.68 (ddd, *J* =8.20, 7.24, 1.20 Hz, 1H), 7.50 (td, *J* = 7.84, 1.08 Hz, 1H), 6.96 (d, *J* = 9.76 Hz, 1H). ^13^C NMR (100 MHz, CDCl_3_) δ (ppm): 159.7, 146.0, 139.8, 139.6, 136.4, 132.2, 131.0, 130.4, 129.1, 125.8, 124.5, 122.8, 117.4, 116.5. MS *m*/*z* 243.0 [M + Na]^+^ (calcd for C_14_H_8_N_2_O, 220.06).

Compound **2**: pale yellow solid, yield: 98.9%. ^1^H NMR (400 MHz, CDCl_3_) δ (ppm): 8.74 (d, *J* = 5.00 Hz, 1H), 8.15 (d, *J* = 2.40 Hz, 1H), 7.98 (d, *J* = 9.76 Hz, 1H), 7.90 (d, *J* = 8.64 Hz, 1H), 7.80 (d, *J* = 5.00 Hz, 1H), 7.04 (dd, *J* = 8.64, 2.40 Hz, 1H), 6.93 (d, *J* = 9.80 Hz, 1H), 3.97 (s, 3H). ^13^C NMR (100 MHz, CDCl_3_) δ (ppm): 162.6, 159.8, 146.1, 141.3, 140.0, 135.8, 132.4, 130.5, 128.6, 123.5, 117.3, 115.7, 114.3, 101.4, 56.1. MS *m*/*z* 273.1 [M + Na]^+^ (calcd for C_15_H_10_N_2_O_2_, 250.07).

Compound **3**: bright yellow solid, yield: 92.0%. ^1^H NMR (400 MHz, CDCl_3_) δ (ppm): 8.78 (d, *J* = 5.00 Hz, 1H), 8.50 (d, *J* = 8.92 Hz, 1H), 7.98 (d, *J* = 9.80 Hz, 1H), 7.89 (d, *J* = 5.00 Hz, 1H), 7.51 (d, *J* = 2.52 Hz, 1H), 7.22 (dd, *J* = 8.96, 2.52 Hz, 1H), 6.95 (d, *J* = 9.76 Hz, 1H), 3.94 (s, 3H). ^13^C NMR (100 MHz, CDCl_3_) δ (ppm): 159.3, 158.0, 145.6, 139.2, 136.3, 133.9, 132.5, 130.5, 129.2, 125.7, 118.1, 118.1, 116.5, 106.6, 56.1. MS *m*/*z* 251.1 [M + H]^+^ (calcd for C_15_H_10_N_2_O_2_, 250.07).

Compound **4**: bright yellow solid, yield: 95.0%. ^1^H NMR (400 MHz, CDCl_3_) δ (ppm): 8.74 (d, *J* = 5.28 Hz, 1H), 7.92 (d, *J* = 9.72 Hz, 1H), 7.89 (d, *J* = 4.96 Hz, 1H), 7.67 (dd, *J* = 7.72, 1.04 Hz, 1H), 7.45 (t, *J* = 7.92 Hz, 1H), 7.23 (d, *J* = 8.08 Hz, 1H), 6.96 (d, *J* = 9.64 Hz, 1H), 4.07 (s, 3H). ^13^C NMR (100 MHz, CDCl_3_) δ (ppm): 158.0, 149.8, 145.2, 138.8, 137.1, 130.7, 130.3, 129.3, 127.5, 127.1, 115.8, 115.2, 114.8, 57.5. MS *m*/*z* 273.0 [M + Na]^+^ (calcd for C_15_H_10_N_2_O_2_, 250.07).

#### 3.2.7. Synthesis of Compound **25** [31]

To a gently refluxing solution, Ac_2_O (14.2 g, 139.0 mmol) containing NaOAc (0.6 g, 7.0 mmol) and powdered D-glucose **23** (2.5 g, 13.9 mmol) were slowly added over a period of 15 min. The mixture was then heated in reflux for additional 5 min before being cooled to room temperature. The reaction was quenched by addition of ice under sonication, during which compound **25** was precipitated. The resulting white powder was filtered and washed with H_2_O until the acetic acid odor was removed, then dried under vacuum to yield the desired product as a white powder (4.2 g, 77.5% yield). ^1^H NMR (400 MHz, MeOD) δ (ppm): 5.82 (d, *J* = 8.32 Hz, 1H), 5.35 (t, *J* = 9.48 Hz, 1H), 5.11–5.01 (m, 2H), 4.28 (dd, *J* = 12.44, 4.52 Hz, 1H), 4.11 (dd, *J* = 12.48, 2.28 Hz, 1H), 4.05–3.99 (m, 1H), 2.08 (s, 3H), 2.05 (s, 3H), 2.02 (s, 6H), 1.98 (s, 3H).

#### 3.2.8. Synthesis of Compounds **27** and **28** [32]

To a solution of **25** or **26** (5.0 mmol) in CH_2_Cl_2_ (7.0 mL) cooled to 0 °C in an ice-water bath, 33% HBr in acetic acid solution (1.24 mL) was added dropwise. The mixture was gradually warmed to 25 °C and stirred for 1.5 h. The reaction was quenched by adding saturated Na_2_CO_3_ solution, and the mixture was extracted with CH_2_Cl_2_. The solvent was removed under reduced pressure, and the residue was purified by flash column chromatography to afford the desired products.

Compound **27**: white solid, yield: 63.2%. ^1^H NMR (400 MHz, DMSO-*d_6_*) δ (ppm): 5.40–5.05 (m, 2H), 4.96–4.76 (m, 2H), 4.72–4.64 (m, 1H), 4.13–4.08 (m, 1H), 4.02–3.97 (m, 1H), 2.00 (t, *J* = 1.12 Hz, 3H), 1.98–1.95 (m, 6H), 1.93 (d, *J* = 12.80 Hz, 3H).

Compound **28**: white solid, yield: 76.2%. ^1^H NMR (400 MHz, DMSO-*d_6_*) δ (ppm): 6.82 (d, *J* = 3.76 Hz, 1H), 5.42 (dd, *J* = 9.84, 8.56 Hz, 1H), 5.28 (d, *J* = 3.96 Hz, 1H), 5.26–5.19 (m, 1H), 5.04–4.96 (m, 1H), 4.91 (d, *J* = 3.84 Hz, 1H), 4.88 (dd, *J* = 3.80, 1.16 Hz, 1H), 4.42 (dd, *J* = 12.96, 2.36 Hz, 1H), 4.26–4.15 (m, 4H), 4.05–4.00 (m, 2H), 2.07 (s, 3H), 2.02 (s, 3H), 2.01 (s, 3H), 2.00 (s, 3H), 1.99 (s, 3H), 1.98 (s, 3H), 1.96 (s, 3H).

#### 3.2.9. Synthesis of Compounds **5**-**7**

To a stirred solution of compound **2** (62.6 mg, 0.25 mmol) and KI (4.2 mg, 0.025 mmol) in 48 wt.% HBr (0.85 mL, 7.5 mmol) under a nitrogen atmosphere, acetic acid (0.12 mL) was slowly added. The mixture was warmed to 110–120 °C and stirred overnight. At the end of the reaction, H_2_O (10.0 mL) was added, and the pH was adjusted to 7–8 by addition of solid Na_2_CO_3_. The solid was isolated by suction filtration, and the filter cake was washed sequentially with cold water and CH_2_Cl_2_. The solid was then dried under vacuum to obtain the desired products: **5**-**7**.

Compound **5**: dark yellow solid, yield: 99.1%. ^1^H NMR (400 MHz, DMSO-*d_6_*) δ (ppm): 10.71 (br s, 1H), 8.82 (d, *J* = 5.48 Hz, 1H), 8.28 (d, *J* = 5.44 Hz, 1H), 8.18 (d, *J* = 8.56 Hz, 1H), 8.11 (d, *J* = 9.84 Hz, 1H), 7.88 (d, *J* = 2.20 Hz, 1H), 7.03 (d, *J* = 9.84 Hz, 1H), 6.99 (dd, *J* = 8.60, 2.24 Hz, 1H). ^13^C NMR (100 MHz, DMSO-*d_6_*) δ (ppm): 161.8, 158.4, 142.5, 141.7, 136.4, 132.4, 131.9, 131.8, 130.1, 125.6, 116.2, 114.9, 114.6, 102.9. MS *m*/*z* 237.0 [M + H]^+^ (calcd for C_14_H_8_N_2_O_2_, 236.06).

Compound **6**: orange yellow solid, yield: 82.9%. ^1^H NMR (400 MHz, DMSO-*d_6_*) δ (ppm): 9.94 (br s, 1H), 8.78 (d, *J* = 5.04 Hz, 1H), 8.27 (d, *J* = 8.80 Hz, 1H), 8.23 (d, *J* = 5.04 Hz, 1H), 8.06 (d, *J* = 9.76 Hz, 1H), 7.65 (d, *J* = 2.48 Hz, 1H), 7.16 (dd, *J* = 8.72, 2.44 Hz, 1H), 6.94 (d, *J* = 9.72 Hz, 1H). ^13^C NMR (100 MHz, DMSO-*d_6_*) δ (ppm): 158.4, 155.7, 145.4, 139.0, 135.5, 132.0, 131.8, 129.7, 128.7, 125.5, 118.6, 117.3, 117.0, 109.1. MS *m*/*z* 235.1 [M − H]^−^ (calcd for C_14_H_8_N_2_O_2_, 236.06).

Compound **7**: yellow solid, yield: 85.5%. ^1^H NMR (400 MHz, DMSO-*d_6_*) δ (ppm): 11.91 (s, 1H), 8.86 (d, *J* = 4.88 Hz, 1H), 8.26 (d, *J* = 4.36 Hz, 1H), 8.24 (s, 1H), 7.72 (d, *J* = 7.52 Hz, 1H), 7.42 (t, *J* = 7.88 Hz, 1H), 7.13–7.07 (m, 2H). ^13^C NMR (100 MHz, DMSO-*d_6_*) δ (ppm): 160.4, 153.8, 147.2, 145.9, 141.5, 136.0, 131.0, 128.6, 127.0, 126.8, 126.8, 118.5, 117.8, 114.0. MS *m*/*z* 259.1 [M + Na]^+^ (calcd for C_14_H_8_N_2_O_2_, 236.06).

#### 3.2.10. Synthesis of Compounds **30**

Compound **7** (0.10 mmol) was dissolved in anhydrous CH_3_CN (2.5 mL), and Ag_2_CO_3_ (68.9 mg, 0.25 mmol) was added. The mixture was heated until it boiled, followed by the addition of a solution of **27**, **28** or **29** (0.30 mmol). The reaction was then stirred and heated under azeotropic conditions for an additional 2–3 h. Afterwards, the inorganic salts were removed by filtration, and the solvent was evaporated from the filtrate, leaving a dry residue. The product was purified by silica gel column chromatography using a stepwise gradient of PE/EtOAc (2:1 to 1:2), yielding a pure product: **30**.

Compound **30a**: brown solid, yield: 83.5%. ^1^H NMR (400 MHz, DMSO-*d_6_*) δ (ppm): 8.81 (d, *J* = 5.00 Hz, 1H), 8.35 (d, *J* = 8.64 Hz, 1H), 8.24 (d, *J* = 5.00 Hz, 1H), 8.16–8.14 (m, 1H), 8.12 (d, *J* = 4.16 Hz, 1H), 7.25 (dd, *J* = 8.64, 2.36 Hz, 1H), 6.99 (d, *J* = 9.88 Hz, 1H), 5.80 (d, *J* = 7.88 Hz, 1H), 5.51 (t, *J* = 9.56 Hz, 1H), 5.16 (dd, *J* = 9.76, 7.88 Hz, 1H), 5.05 (t, *J* = 9.72 Hz, 1H), 4.39–4.32 (m, 1H), 4.23 (dd, *J* = 12.36, 5.72 Hz, 1H), 4.11 (dd, *J* = 12.32, 2.40 Hz, 1H), 2.06 (s, 3H), 2.03 (s, 6H), 1.99 (s, 3H). ^13^C NMR (100 MHz, DMSO-*d_6_*) δ (ppm): 170.1, 169.6, 169.4, 169.2, 159.0, 158.4, 146.1, 140.1, 140.1, 135.5, 132.1, 130.0, 128.4, 124.8, 119.3, 116.9, 114.6, 104.6, 97.5, 71.9, 71.1, 70.8, 68.1, 61.7, 20.5, 20.4, 20.4, 20.3. MS *m*/*z* 589.4 [M + Na]^+^ (calcd for C_28_H_26_N_2_O_11_, 566.15).

Compound **30b**: brown solid, yield: 87.7%. ^1^H NMR (400 MHz, DMSO-*d_6_*) δ (ppm): 8.78 (d, *J* = 5.00 Hz, 1H), 8.23 (d, *J* = 4.96 Hz, 1H), 8.09 (d, *J* = 4.48 Hz, 1H), 7.66 (d, *J* = 1.96 Hz, 1H), 7.37–7.34 (m, 1H), 6.96 (t, *J* = 10.04 Hz, 2H), 5.70 (d, *J* = 8.00 Hz, 1H), 5.45 (t, *J* = 9.56 Hz, 1H), 5.16 (dd, *J* = 9.76, 7.96 Hz, 1H), 5.09–5.04 (m, 1H), 4.25 (dd, *J* = 12.28, 5.20 Hz, 1H), 4.18 (d, *J* = 2.44, 1H), 4.15 (d, *J* = 2.40, 1H), 2.09 (s, 3H), 2.03 (s, 3H), 2.01 (s, 3H), 1.99 (s, 3H). ^13^C NMR (100 MHz, DMSO-*d_6_*) δ (ppm): 170.0, 169.6, 169.3, 169.2, 158.4, 155.7, 145.6, 139.2, 135.7, 134.4, 131.9, 129.5, 128.6, 125.6, 119.7, 118.5, 117.0, 109.1, 97.8, 72.0, 71.0, 70.8, 68.0, 61.6, 20.5, 20.4, 20.4, 20.3. MS *m*/*z* 589.4 [M + Na]^+^ (calcd for C_28_H_26_N_2_O_11_, 566.15).

Compound **30c**: brown solid, yield: 86.5%. ^1^H NMR (400 MHz, DMSO-*d_6_*) δ (ppm): 8.78 (d, *J* = 5.00 Hz, 1H), 8.31 (d, *J* = 8.52 Hz, 1H), 8.20 (d, *J* = 5.00 Hz, 1H), 8.15–8.08 (m, 2H), 7.21 (dd, *J* = 8.60, 2.32 Hz, 1H), 6.96 (d, *J* = 9.76 Hz, 1H), 5.70 (d, *J* = 7.76 Hz, 1H), 5.44–5.37 (m, 2H), 5.35–5.27 (m, 1H), 4.56 (t, *J* = 7.32 Hz, 1H), 4.19–4.08 (m, 2H), 2.17 (s, 3H), 2.09 (s, 3H), 2.04 (s, 3H), 1.97 (s, 3H). ^13^C NMR (100 MHz, DMSO-*d_6_*) δ (ppm): 170.0, 170.0, 169.5, 169.3, 158.9, 158.5, 146.0, 140.1, 139.9, 135.4, 132.0, 129.1, 128.3, 124.7, 119.2, 116.8, 114.6, 104.3, 98.0, 70.7, 70.0, 68.4, 67.3, 61.5, 20.5, 20.5, 20.4, 20.4. MS *m*/*z* 589.3 [M + Na]^+^ (calcd for C_28_H_26_N_2_O_11_, 566.15).

Compound **30d**: light yellow solid, yield: 85.9%. ^1^H NMR (400 MHz, DMSO-*d_6_*) δ (ppm): 8.85 (d, *J* = 5.00 Hz, 1H), 8.44 (d, *J* = 8.92 Hz, 1H), 8.30 (d, *J* = 5.04 Hz, 1H), 8.13 (d, *J* = 9.80 Hz, 1H), 8.03 (d, *J* = 2.52 Hz, 1H), 7.42 (dd, *J* = 8.92, 2.52 Hz, 1H), 7.00 (d, *J* = 9.76 Hz, 1H), 5.62 (d, *J* = 7.12 Hz, 1H), 5.39 (d, *J* = 2.88 Hz, 1H), 5.35–5.26 (m, 2H), 4.51 (t, *J* = 6.40 Hz, 1H), 4.15 (d, *J* = 6.36 Hz, 2H), 2.17 (s, 3H), 2.10 (s, 3H), 2.00 (s, 3H), 1.97 (s, 3H). ^13^C NMR (100 MHz, DMSO-*d_6_*) δ (ppm): 170.0, 169.9, 169.6, 169.4, 158.7, 154.3, 145.9, 139.7, 136.0 134.5, 132.1, 129.2, 128.7, 125.5, 119.7, 117.5, 117.1, 111.5, 98.4, 70.5, 70.2, 68.4, 67.2, 61.3, 20.6, 20.5, 20.5, 20.4. MS *m*/*z* 589.4 [M + Na]^+^ (calcd for C_28_H_26_N_2_O_11_, 566.15).

Compound **30e**: yellow solid, yield: 44.5%. ^1^H NMR (400 MHz, DMSO-*d_6_*) δ (ppm): 8.86 (d, *J* = 4.96 Hz, 1H), 8.46 (d, *J* = 8.80 Hz, 1H), 8.33 (d, *J* = 5.00 Hz, 1H), 8.19 (d, *J* = 2.32 Hz, 1H), 8.15 (dt, *J* = 9.80, 2.76 Hz, 1H), 7.55 (dd, *J* = 8.84, 2.40 Hz, 1H), 7.00 (d, *J* = 9.76 Hz, 1H), 5.92 (d, *J* = 5.48 Hz, 1H), 5.41 (d, *J* = 3.84 Hz, 1H), 5.34–5.23 (m, 2H), 4.99 (d, *J* = 9.80 Hz, 1H), 4.96 (d, *J* = 4.48 Hz, 1H), 4.83 (dd, *J* = 10.36, 3.84 Hz, 1H), 4.50–4.46 (m, 1H), 4.28 (dd, *J* = 12.32, 2.24 Hz, 1H), 4.17–4.11 (m, 2H), 4.00–3.97 (m, 1H), 3.95–3.90 (m, 1H), 3.73 (d, *J* = 8.12 Hz, 1H), 2.05 (s, 3H), 2.03 (s, 3H), 2.02 (s, 3H), 2.00 (s, 3H), 1.99 (s, 3H), 1.98 (s, 3H), 1.95 (s, 3H). ^13^C NMR (100 MHz, DMSO-*d_6_*) δ (ppm): 170.3, 170.3, 170.0, 169.8, 169.5, 169.4, 169.2, 158.7, 150.3, 145.8, 139.8, 138.6, 135.9, 135.6, 132.0, 129.2, 128.6, 125.8, 125.3, 122.5, 117.9, 117.6, 116.8, 72.4, 71.8, 69.6, 69.0, 67.8, 67.5, 63.7, 63.5, 61.7, 59.7, 21.3, 20.8, 20.7, 20.6, 20.4, 20.3, 20.3. MS *m*/*z* 877.1 [M + Na]^+^ (calcd for C_40_H_42_N_2_O_19_, 854.24).

#### 3.2.11. Synthesis of Compounds **12**, **13** and **31(c**-**e)**

A solution of compound **30** (0.05 mmol) in a mixture of MeOH (2.7 mL) and H_2_O (0.5 mL) was heated to reflux with K_2_CO_3_ (40.1 mg) for 2–3 h. After removing MeOH and H_2_O under reduced pressure, the residue was subjected to silica gel column and purified using a stepwise gradient of CH_2_Cl_2_/MeOH (4:1 to 1:1), yielding pure products.

Compound **12**: yellow solid, yield: 86.6%. ^1^H NMR (400 MHz, DMSO-*d_6_*) δ (ppm): 8.80 (d, *J* = 5.00 Hz, 1H), 8.31 (d, *J* = 8.64 Hz, 1H), 8.23 (d, *J* = 5.00 Hz, 1H), 8.19 (d, *J* = 2.24 Hz, 1H), 8.14 (d, *J* = 9.76 Hz, 1H), 7.32 (dd, *J* = 8.60, 2.32 Hz, 1H), 6.99 (d, *J* = 9.80 Hz, 1H), 5.46 (br s, 1H), 5.15 (br s, 2H), 5.03 (d, *J* = 7.20 Hz, 1H), 4.60 (br s, 1H), 3.73 (dd, *J* = 11.96, 2.20 Hz, 1H), 3.54 (dd, *J* = 12.00, 5.16 Hz, 1H), 3.30–3.26 (m, 2H), 3.26–3.18 (m, 2H). ^13^C NMR (100 MHz, DMSO-*d_6_*) δ (ppm): 159.8, 159.0, 146.1, 140.1, 140.1, 135.4, 132.0, 129.5, 128.4, 124.6, 118.4, 116.7, 114.4, 104.6, 101.1, 77.2, 76.5, 73.3, 69.5, 60.5. MS *m*/*z* 421.1 [M + Na]^+^ (calcd for C_20_H_18_N_2_O_7_, 398.11).

Compound **13**: yellow solid, yield: 83.2%. ^1^H NMR (400 MHz, DMSO-*d_6_*) δ (ppm): 8.85 (d, *J* = 5.00 Hz, 1H), 8.41 (d, *J* = 8.92 Hz, 1H), 8.30 (d, *J* = 5.04 Hz, 1H), 8.14 (d, *J* = 9.76 Hz, 1H), 8.09 (d, *J* = 2.52 Hz, 1H), 7.45 (dd, *J* = 8.92, 2.60 Hz, 1H), 7.00 (d, *J* = 9.80 Hz, 1H), 5.02 (d, *J* = 7.16 Hz, 1H), 3.71 (dd, *J* = 12.00, 2.20 Hz, 1H), 3.54 (d, *J* = 5.56 Hz, 1H), 3.51 (d, *J* = 5.52 Hz, 1H), 3.30–3.26 (m, 2H), 3.20 (d, *J* = 8.84 Hz, 1H). ^13^C NMR (100 MHz, DMSO-*d_6_*) δ (ppm): 158.7, 155.6, 145.9, 139.6, 135.9, 133.8, 132.0, 129.6, 128.8, 125.4, 120.7, 117.6, 116.9, 110.8, 101.2, 77.3, 76.8, 73.4, 69.7, 60.7. MS *m*/*z* 421.1 [M + Na]^+^ (calcd for C_20_H_18_N_2_O_7_, 398.11).

Compound **31c**: white solid, yield: 94.1%. ^1^H NMR (400 MHz, DMSO-*d_6_*) δ (ppm): 8.76 (d, *J* = 5.00 Hz, 1H), 8.24 (d, *J* = 8.64 Hz, 1H), 8.16 (d, *J* = 5.00 Hz, 1H), 8.14 (d, *J* = 2.36 Hz, 1H), 8.09 (d, *J* = 9.76 Hz, 1H), 7.28 (dd, *J* = 8.68, 2.32 Hz, 1H), 6.94 (d, *J* = 9.80 Hz, 1H), 5.00 (d, *J* = 7.56 Hz, 1H), 3.77 (d, *J* = 3.32 Hz, 1H), 3.70–3.64 (m, 2H), 3.62–3.53 (m, 2H), 3.53–3.48 (m, 1H). ^13^C NMR (100 MHz, DMSO-*d_6_*) δ (ppm): 159.9, 158.9, 146.0, 140.0, 135.3, 131.9, 129.4, 128.3, 124.5, 118.2, 116.6, 114.5, 104.4, 101.6, 75.6, 73.2, 70.4, 68.0, 60.2. MS *m*/*z* 421.0 [M + Na]^+^ (calcd for C_20_H_18_N_2_O_7_, 398.11).

Compound **31d**: white solid, yield: 92.7%. ^1^H NMR (400 MHz, DMSO-*d_6_*) δ (ppm): 8.83 (d, *J* = 5.00 Hz, 1H), 8.38 (d, *J* = 8.88 Hz, 1H), 8.25 (d, *J* = 5.00 Hz, 1H), 8.11 (d, *J* = 9.76 Hz, 1H), 8.05 (d, *J* = 2.44 Hz, 1H), 7.44 (dd, *J* = 8.88, 2.48 Hz, 1H), 6.98 (d, *J* = 9.76 Hz, 1H), 5.31(br s, 1H), 4.99 (d, *J* = 7.64 Hz, 1H), 4.95 (br s, 1H), 4.74 (br s, 1H), 4.59 (br s, 1H), 3.75 (d, *J* = 3.28 Hz, 1H), 3.70–3.63 (m, 2H), 3.61–3.52 (m, 2H), 3.46 (dd, *J* = 9.60, 3.24 Hz, 1H). ^13^C NMR (100 MHz, DMSO-*d_6_*) δ (ppm): 158.6, 155.6, 145.8, 139.5, 135.9, 133.7, 132.0, 129.4, 128.7, 125.3, 120.0, 117.4, 116.9, 110.9, 101.8, 75.7, 73.4, 70.4, 68.2, 60.4. MS *m*/*z* 421.1 [M + Na]^+^ (calcd for C_20_H_18_N_2_O_7_, 398.11).

Compound **31e**: yellow solid, yield: 75.6%. ^1^H NMR (400 MHz, DMSO-*d_6_*) δ (ppm): 8.85 (d, *J* = 5.00 Hz, 1H), 8.42 (d, *J* = 8.92 Hz, 1H), 8.29 (d, *J* = 5.00 Hz, 1H), 8.14 (d, *J* = 9.76 Hz, 1H), 8.09 (d, *J* = 2.44 Hz, 1H), 7.46 (dd, *J* = 8.92, 2.48 Hz, 1H), 7.00 (d, *J* = 9.76 Hz, 1H), 5.10 (d, *J* = 7.68 Hz, 1H), 5.07 (d, *J* = 3.76 Hz, 1H), 3.79 (d, *J* = 11.08 Hz, 1H), 3.70–3.58 (m, 4H), 3.54–3.42 (m, 6H), 3.17 (s, 1H), 3.09 (t, *J* = 9.00 Hz, 2H). ^13^C NMR (100 MHz, DMSO-*d_6_*) δ (ppm): 158.7, 155.4, 150.1, 145.9, 135.9, 133.9, 132.0, 129.5, 128.7, 125.4, 120.0, 117.5, 116.9, 110.8, 100.9, 100.8, 79.2, 76.4, 75.4, 73.7, 73.3, 72.9, 72.5, 69.9, 60.9, 60.3. MS *m*/*z* 559.2 [M − H]^−^ (calcd for C_26_H_28_N_2_O_12_, 560.16).

### 3.3. Microbroth Dilution Assay

The bioassay studies employed four microbial human pathogen strains: *Staphylococcus aureus* (ATCC 43300), *Escherichia coli* (ATCC 25922), *Candida albicans* (ATCC 10231) and *Cryptococcus neoformans* (ATCC 66031). Bacterial pathogens *S. aureus* and *E. coli* were maintained in Tryptic Soy media (TSA, TSB; BD Biosciences, San Jose, CA, USA) at 37 °C. Fungal microorganisms *C. albicans* and *C. neoformans* were propagated in yeast malt (YM; BD Biosciences) and Sabouraud dextrose media (SDA; BD Biosciences), respectively, at 30 °C. All liquid cultures were incubated with constant agitation at 200 rpm using an orbital shaker.

Antimicrobial activity evaluation was performed through broth microdilution method [34]. Test compounds were initially solubilized in DMSO to achieve a 20 mg/mL stock solution, which was subsequently stored at 4 °C until experimental use. Microbial suspensions were prepared from overnight cultures, adjusted to an optical density (OD_600_) ranging from 0.03 to 0.06, representing the exponential growth phase. These suspensions were then diluted to 1:10 in appropriate broth media (except for *C. neoformans*, which was used undiluted) and aliquoted into 96-well microplates (195 μL/well). Following incubation periods of 24 h for bacterial strains and 48 h for fungal strains, optical density measurements were recorded at 600 nm. The percentage inhibition was determined by subtracting the blank well absorbance from sample readings, normalizing against the average absorbance of DMSO control wells and multiplying by 100.

## 4. Conclusions

In summary, the efficient synthesis of canthin-6-one alkaloids **4**, **5**, **7** and **12**-**13** and 8 novel derivatives was achieved for the first time. The synthetic process involved key Pd-catalyzed Suzuki–Miyaura coupling, Cu-catalyzed amidation and Koenig–Knorr glycosylation reactions, using **16** as the starting material. Antimicrobial activity was evaluated, revealing that canthin-6-one alkaloids **1**-**4** and **7** exhibited antimicrobial properties against *C. albicans*, *C. neoformans* and *S. aureus* comparable to those of the positive control drugs at 50 µg/mL. The antimicrobial activity of alkaloids **2** and **4**-**5** against these four microbial human pathogens and canthin-6-one alkaloids **6**-**7** against *C. albicans*, *C. neoformans* and *E. coli* is reported here for the first time. However, none of the derivatives demonstrated antimicrobial activity, and all synthesized compounds were ineffective against *E. coli* at 50 µg/mL. It can be concluded that the substituents on the A-ring of canthin-6-one play a crucial role in its antimicrobial properties. This study lays the foundation for the further development of antimicrobial agents.

## Figures and Tables

**Table 1:** Compounds for Koenig-Knorr glycosylation and hydrolysis reactions

NO.	Compound Names
**12**	canthin-6-one-9-O-β-D-glucopyranoside
**13**	canthin-6-one-10-O-β-D-glucopyranoside
**30a**	canthin-6-one-9-O-tetraacetyl-β-D-glucopyranoside
**30b**	canthin-6-one-10-O-tetraacetyl-β-D-glucopyranoside
**30c**	canthin-6-one-9-O-tetraacetyl-α-D-galactopyranoside
**30d**	canthin-6-one-10-O-tetraacetyl-α-D-galactopyranoside
**30e**	canthin-6-one-10-O-heptaacetyl-β-D-maltoside
**31c**	canthin-6-one-9-O-α-D-galactopyranoside
**31d**	canthin-6-one-10-O-α-D-galactopyranoside
**31e**	canthin-6-one-10-O-β-D-maltoside

**Table 2:** Comparison of ^13^C NMR spectroscopic data obtained for natural **12** and synthetic **12** in DMSO-*d_6._*

No.	Natural	Synthetic	Δδ
1	114.3	114.4	0.1
2	145.9	146.1	0.2
4	139.9	140.1	0.2
5	128.2	128.4	0.2
6	158.9	159.0	0.1
8	104.5	104.6	0.1
9	159.7	159.8	0.1
10	116.5	116.7	0.2
11	124.4	124.6	0.2
12	118.2	118.4	0.2
13	139.9	140.1	0.2
14	129.3	129.5	0.2
15	131.8	132.0	0.2
16	135.3	135.4	0.1
G-1	101.0	101.1	0.1
G-2	73.2	73.3	0.1
G-3	77.2	77.2	0
G-4	69.5	69.5	0
G-5	76.5	76.5	0
G-6	60.5	60.5	0

**Table 3:** Antimicrobial activity of the synthesized compounds against four microbial human pathogens.

Compounds	Average Inhibition Rate (%)			
No.	*C. albicans*	*C. neoformans*	*S. aureus*	*E. coli*
**1**	95.7	96.9	99.7	15.8
**2**	58.4	76.3	99.8	14.0
**3**	39.7	57.9	88.1	4.1
**4**	55.9	96.6	46.3	1.2
**5**	−13.5	−37.8	10.8	5.2
**6**	7.3	35.0	14.8	−0.1
**7**	81.3	83.2	98.3	13.9
**12**	−3.2	22.2	−9.8	5.6
**13**	1.2	4.7	−5.1	1.8
**31c**	−2.6	13.4	0.4	4.2
**31d**	0.2	0.2	−3.3	4.2
**31e**	1.3	0.5	0.8	1.2
Ketoconazole	99.9	98.2		
Vanconomycin			94.6	
Chloromycin				99.2

Note: Data were shown as mean ± SD. Ketoconazole, Vanconomycin and Chloromycin served as the positive control agents in the antimicrobial assays. All compounds and positive controls were tested at 50 µg/mL.

## Data Availability

All data are contained within the article and Supplementary Materials.

## Supplementary Materials

The following supporting information can be downloaded at: https://www.mdpi.com/article/10.3390/molecules30071546/s1, Procedure S1: Preperation of compounds **27** and **28**; Figure S1: HPLC of canthin-6-one **5**; Figure S2: ^1^H NMR spectrum of compound **19**; Figure S3: ^1^H NMR spectrum of compound **20**; Figure S4: ^1^H NMR spectrum of compound **16**; Figures S5 and S6: ^1^H and ^13^C NMR spectrum of compound **21a**; Figures S7 and S8: ^1^H and ^13^C NMR spectrum of compound **21b**; Figures S9 and S10: ^1^H and ^13^C NMR spectrum of compound **21c**; Figures S11 and S12: ^1^H and ^13^C NMR spectrum of compound **21d**; Figures S13 and S14: ^1^H and ^13^C NMR spectrum of compound **22a**; Figures S15 and S16: ^1^H and ^13^C NMR spectrum of compound **22b**; Figures S17 and S18: ^1^H and ^13^C NMR spectrum of compound **22c**; Figures S19 and S20: ^1^H and ^13^C NMR spectrum of compound **22d**; Figures S21 and S22: ^1^H and ^13^C NMR spectrum of compound **1**; Figures S23 and S24: ^1^H and ^13^C NMR spectrum of compound **2**; Figures S25 and S26: ^1^H and ^13^C NMR spectrum of compound **3**; Figures S27 and S28: ^1^H and ^13^C NMR spectrum of compound **4**; Figure S29: ^1^H NMR spectrum of compound **25**; Figure S30: ^1^H NMR spectrum of compound **27**; Figure S31: ^1^H NMR spectrum of compound **28**; Figures S32 and S33: ^1^H and ^13^C NMR spectrum of compound **5**; Figures S34 and S35: ^1^H and ^13^C NMR spectrum of compound **6**; Figures S36 and S37: ^1^H and ^13^C NMR spectrum of compound **7**; Figures S38 and S39: ^1^H and ^13^C NMR spectrum of compound **30a**; Figures S40 and S41: ^1^H and ^13^C NMR spectrum of compound **30b**; Figures S42 and S43: ^1^H and ^13^C NMR spectrum of compound **30c**; Figures S44 and S45: ^1^H and ^13^C NMR spectrum of compound **30d**; Figures S46 and S47: ^1^H and ^13^C NMR spectrum of compound **30e**; Figures S48 and S49: ^1^H and ^13^C NMR spectrum of compound **12**; Figure S50 and S51: ^1^H and ^13^C NMR spectrum of compound **13**; Figures S52 and S53: ^1^H and ^13^C NMR spectrum of compound **31a**; Figures S54 and S55: ^1^H and ^13^C NMR spectrum of compound **31b**; Figures S56 and S57: ^1^H and ^13^C NMR spectrum of compound **31c**; Figures S58–S74: MS of all target compounds. NMR spectrum of compound **31e**; Figures S58–S74: MS of all target compounds.
