# Supplementary material for: Synthesis and Antimicrobial Activity of Canthin-6-One Alkaloids
Source: Molecules. 2025 Mar 31;30(7):1546. doi: 10.3390/molecules30071546 (PMC11990583; doi:10.3390/molecules30071546)
Supplement: Supplementary file 1 [file molecules-30-01546-s001.zip › molecules-3524641-supplementary.pdf]

Supplementary Materials

Synthesis and Antimicrobial Activity of Canthin-6-One Alkaloids

Xubing Qi [1,†], Yogini Jaiswal [2,†], Xinrong Xie [1], Yu Fan [1], Rongping Wu [1], Shaoyang Su [3], Yifu Guan [1,*], Leonard Williams [2,*] and Xun Song [4,*]

[1] Key Laboratory of Chemistry and Engineering of Forest Products (State Ethnic Affairs Commission), Guangxi Collaborative Innovation Center for Chemistry and Engineering of Forest Products, School of Chemistry and Chemical Engineering, Guangxi Minzu University, Nanning 530006, China; 18789419455@163.com (X.Q.); 20199030@gxmzu.edu.cn (X.X.); ffyu012@163.com (Y.F.); 18169417717@163.com (R.W.)

[2] Center for Excellence in Post-Harvest Technologies, North Carolina Agricultural and Technical State University, The North Carolina Research Campus, Kannapolis, NC 28081, USA; ysjaiswa@ncat.edu (Y.J.)

[3] Department of Applied Chemistry Teaching and Research, Guangxi Vocational University of Agriculture, Nanning 530007, China; susy@gxnzd.edu.cn (S.S)

[4] College of Pharmacy, Shenzhen Technology University, Shenzhen 518118, China

* Correspondence: guanyifu@gxmzu.edu.cn (Y.G.); llw@ncat.edu (L.W.); songxun@sztu.edu.cn (X.S.)

† These authors contributed equally to this work.

Content

Procedure S1 Preparation of compounds **27** and **28**.. 3

Figure S1 HPLC of canthin-6-one **5** 5

Figure S2-S57 The NMR spectra of all synthesized compounds.................................. 5

Figure S58-S74 Mass spectra of all target compounds.. 34

Procedure 1. Preparation of compounds **27** and **28.**

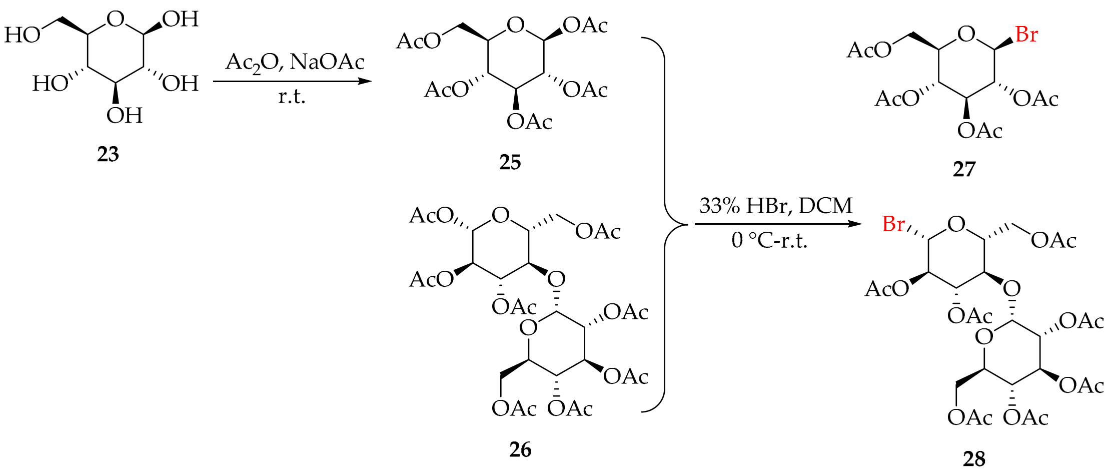

Scheme 2: Preparation of compounds **27** and **28.**

Synthesis of compound **25**

To a gently refluxing solution, Ac_2O (14.2 g, 139.0 mmol) containing NaOAc (0.6 g, 7.0 mmol) and powdered D-glucose **23** (2.5 g, 13.9 mmol) were slowly added over a period of 15 minutes. The mixture was then heated in reflux for additional 5 minutes before being cooled to room temperature. The reaction was quenched by the addition of ice under sonication, during which compound **25** was precipitated. The resulting white powder was filtered and washed with H_2O until the acetic acid odor was removed, then dried under vacuum to yield the desired product as a white powder (4.2 g, 77.5% yield). ^{1}H NMR (400 MHz, MeOD) δ (ppm): 5.82 (d, J = 8.32 Hz, 1H), 5.35 (t, J = 9.48 Hz, 1H), 5.11-5.01 (m, 2H), 4.28 (dd, J = 12.44, 4.52 Hz, 1H), 4.11 (dd, J = 12.48, 2.28 Hz, 1H), 4.05-3.99 (m, 1H), 2.08 (s, 3H), 2.05 (s, 3H), 2.02 (s, 6H), 1.98 (s, 3H).

Synthesis of compounds **27** and **28**

To a solution of **25** or **26** (5.0 mmol) in CH_2Cl_2 (7.0 mL) cooled to 0 °C in an ice-water bath, 33% HBr in acetic acid solution (1.24 mL) was added dropwise. The mixture was gradually warmed to 25 °C and stirred for 1.5 hours. The reaction was quenched by adding saturated Na_2CO_3 solution, and the mixture was extracted with CH_2Cl_2. The solvent was removed under reduced pressure, and the residue was purified by flash column chromatography to afford the desired products.

Compound 27： white solid, yield: 63.2%. ^{1}H NMR (400 MHz, DMSO-d_6) δ

(ppm): 5.40-5.05 (m, 2H), 4.96-4.76 (m, 2H), 4.72-4.64 (m, 1H), 4.13-4.08 (m, 1H), 4.02-3.97 (m, 1H), 2.00 (t, J = 1.12 Hz, 3H), 1.98-1.95 (m, 6H), 1.93 (d, J = 12.80 Hz, 3H).

Compound 28： white solid, yield: 76.2%. ^{1}H NMR (400 MHz, DMSO-d_6) δ (ppm): 6.82 (d, J = 3.76 Hz, 1H), 5.42 (dd, J = 9.84, 8.56 Hz, 1H), 5.28 (d, J = 3.96 Hz, 1H), 5.26-5.19 (m, 1H), 5.04-4.96 (m, 1H), 4.91 (d, J = 3.84 Hz, 1H), 4.88 (dd, J = 3.80, 1.16 Hz, 1H), 4.42 (dd, J =12.96, 2.36 Hz, 1H), 4.26-4.15 (m, 4H), 4.05-4.00 (m, 2H), 2.07 (s, 3H), 2.02 (s, 3H), 2.01 (s, 3H), 2.00 (s, 3H), 1.99 (s, 3H), 1.98 (s, 3H), 1.96 (s, 3H).

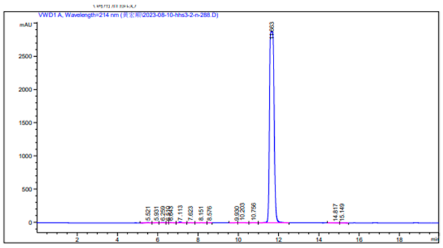

面积百分比报告

排序 : 信号
乘积因子 : 1.0000
稀释因子 : 1.0000
样品量: : 20.00000 [ng/ul] (校正中没有使用)
内标使用乘积因子和稀释因子

信号 1: VWD1 A, Wavelength=214 nm

峰 #	保留时间 [min]	类型	峰宽 [min]	峰面积 [mAU*s]	峰高 [mAU]	峰面积 %
1	5.521	VB R	0.1945	76.73038	5.35941	0.1688
2	5.931	BV	0.1397	28.40681	3.03617	0.0625
3	6.259	VV	0.1658	75.06753	6.37912	0.1652
4	6.510	VV	0.0934	14.36664	2.20811	0.0316
5	6.643	VB	0.1627	49.88564	4.47121	0.1098
6	7.113	BB	0.1179	108.85787	13.52102	0.2395

260-VWD 2023/8/10 15:33:09 系统 页 1/2

居文件: C:\Chem32\2\Data\黄宏顺\2023-08-10-hhs3-2-n-288.D
品名称: hhs3-2-n

峰 #	保留时间 [min]	类型	峰宽 [min]	峰面积 [mAU*s]	峰高 [mAU]	峰面积 %
7	7.623	BB	0.1136	25.12415	3.40605	0.0553
8	8.151	BV R	0.1624	35.31717	3.22272	0.0777
9	8.576	VV	0.1135	20.25756	2.71683	0.0446
10	9.930	BV	0.1648	42.59509	3.09940	0.0937
11	10.203	VV	0.2858	78.68120	3.25123	0.1731
12	10.756	VB	0.1802	45.82644	3.64349	0.1008
13	11.663	BB	0.1839	4.47848e4	2880.92065	98.5326
14	14.817	BV	0.2276	41.73925	2.21703	0.0918
15	15.149	VB	0.1784	24.11667	1.71615	0.0531

Figure S1: HPLC of canthin-6-one **5**.

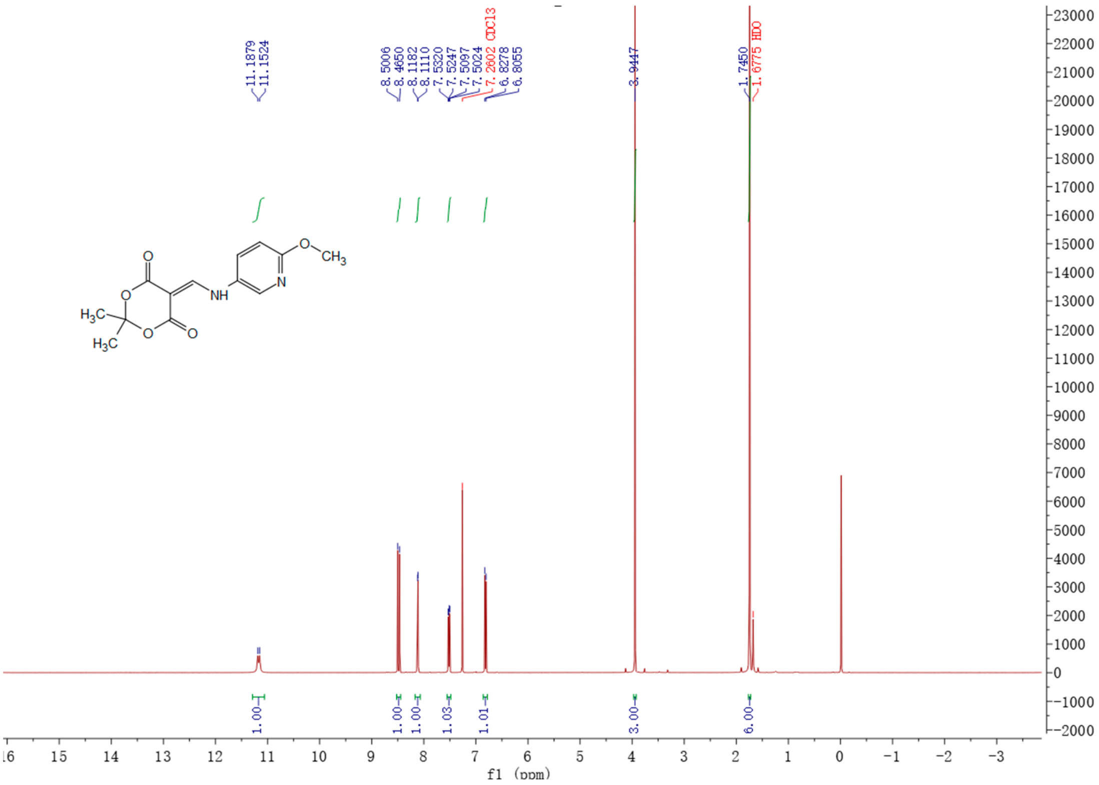

Figure S2: The ^{1}H NMR spectrum of compound **19** in $CDCl_3$ (400 MHz).

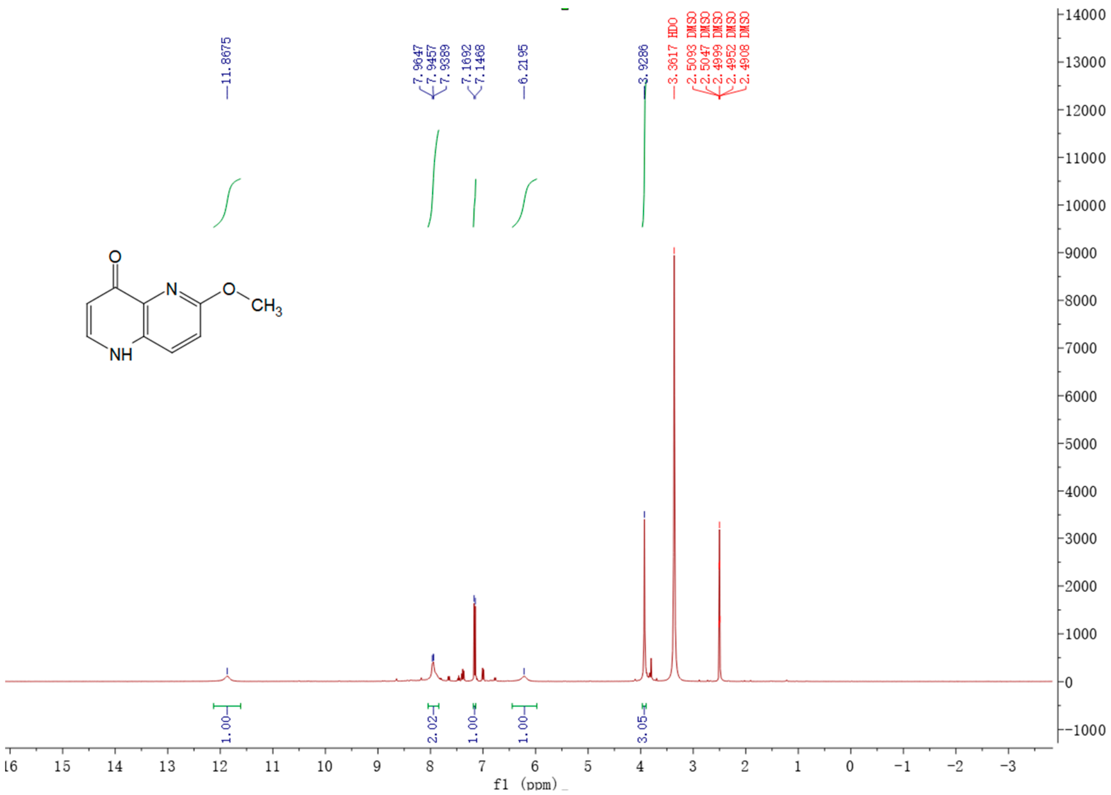

Figure S3: The ^{1}H NMR spectrum of compound **20** in DMSO-*d6* (400 MHz).

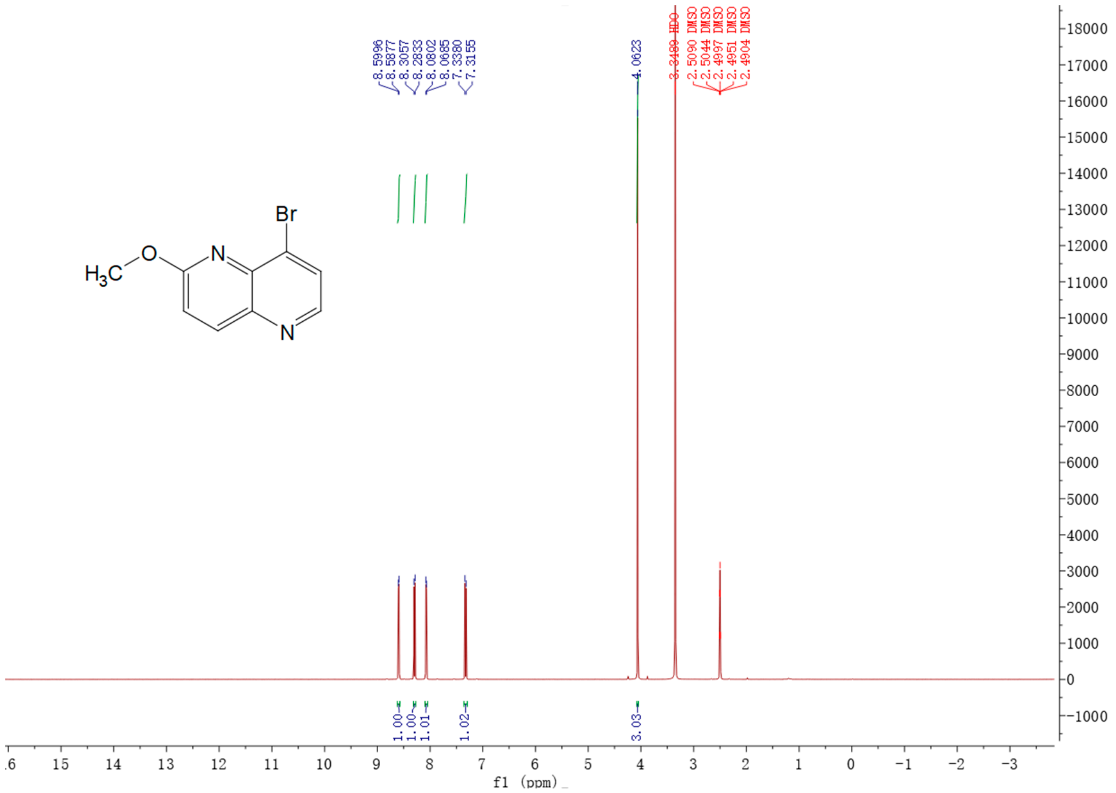

Figure S4: The ^{1}H NMR spectrum of compound **16** in DMSO-*d6* (400 MHz).

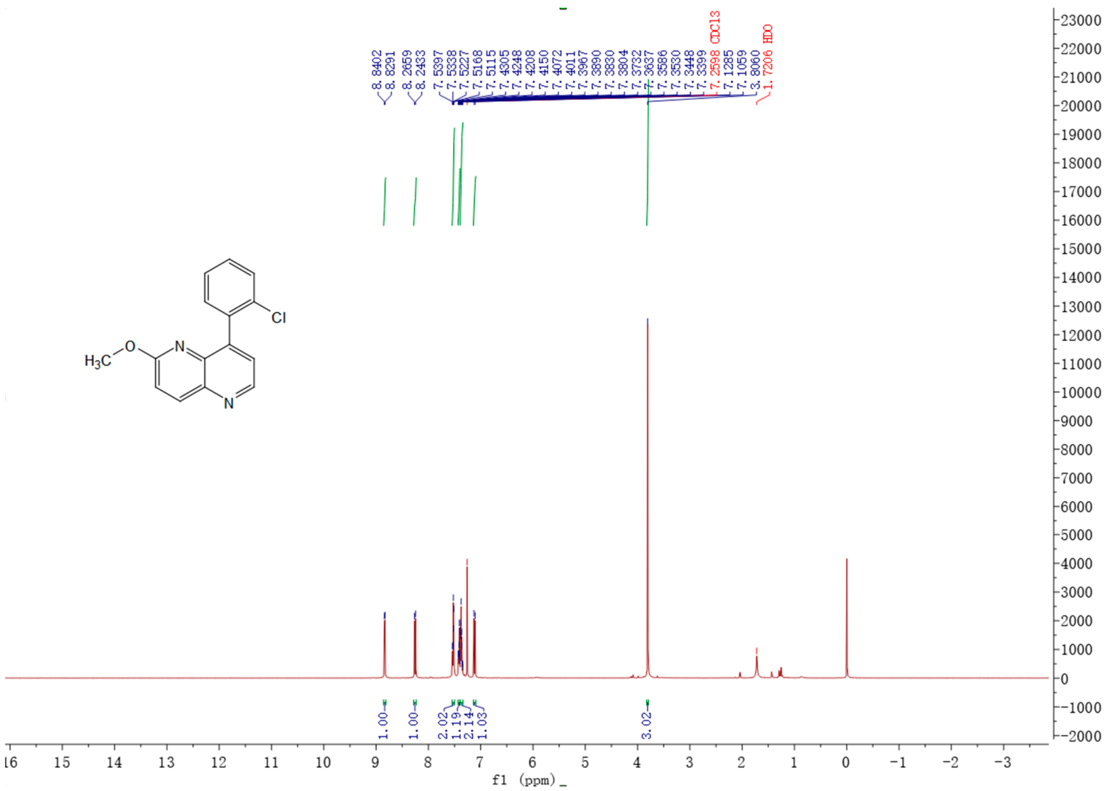

Figure S5: The ^{1}H NMR spectrum of compound **21a** in $CDCl_3$ (400 MHz).

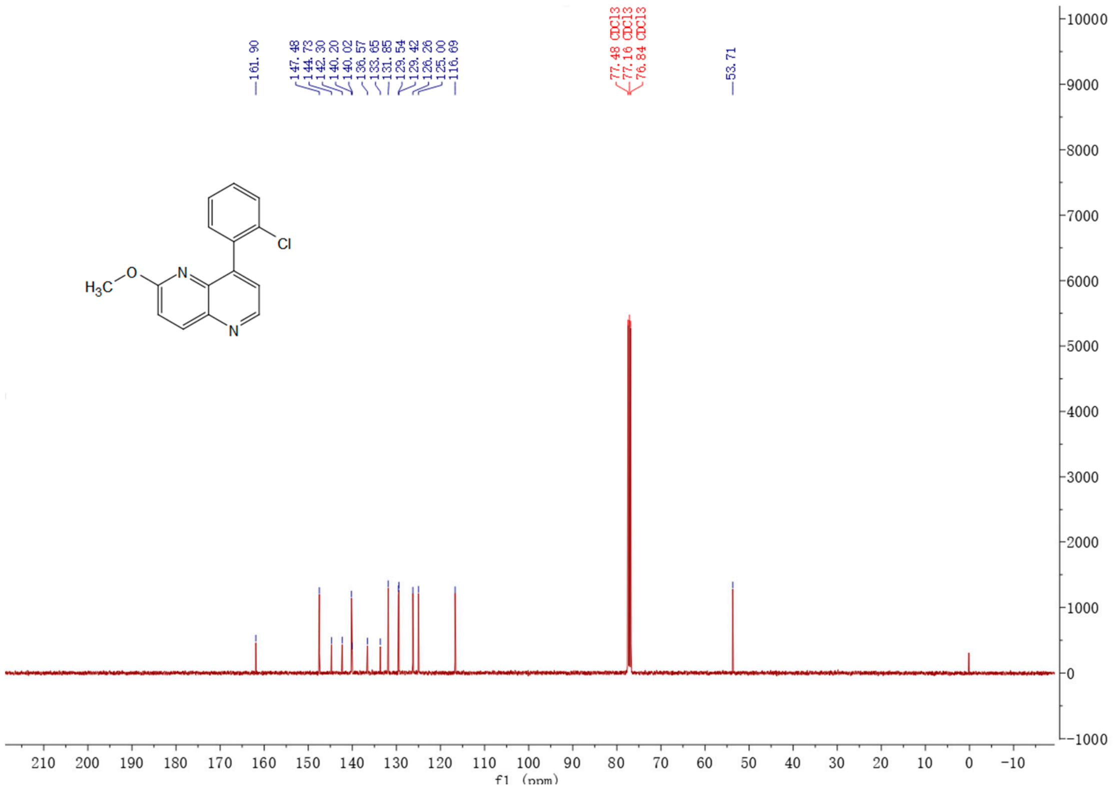

Figure S6: The ^{13}C NMR spectrum of compound **21a** in $CDCl_3$ (100 MHz).

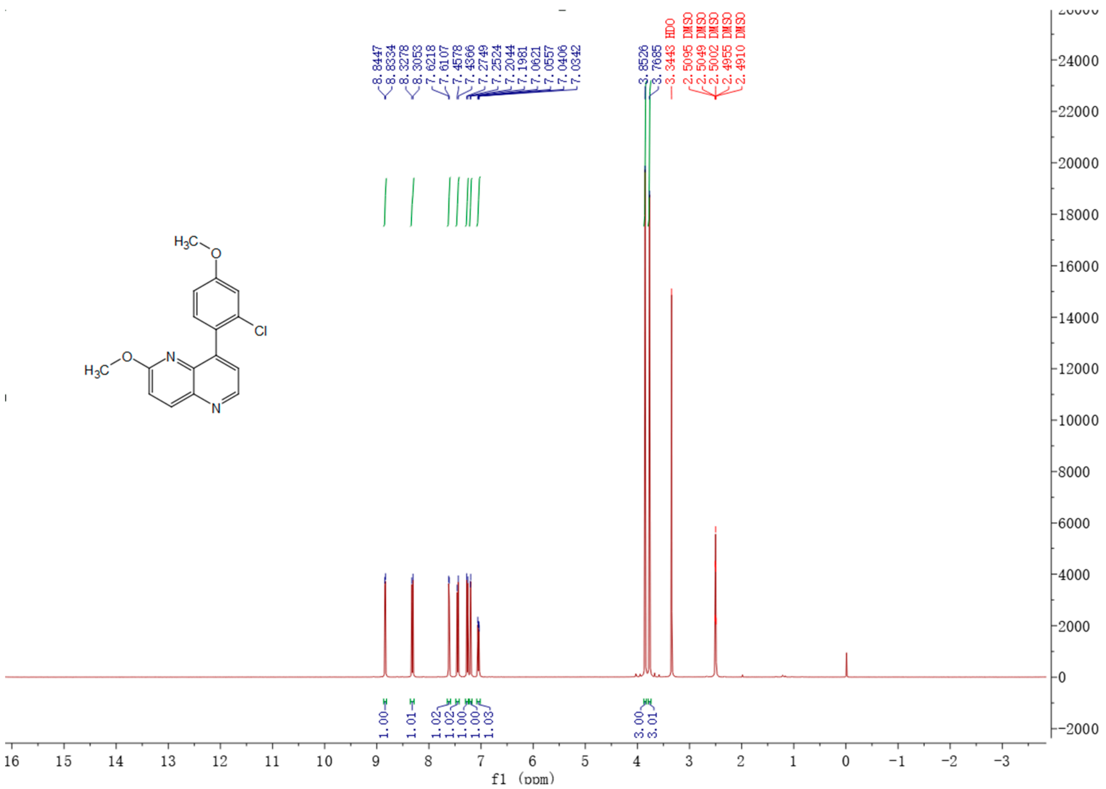

Figure S7: The ^{1}H NMR spectrum of compound **21b** in DMSO-*d6* (400 MHz).

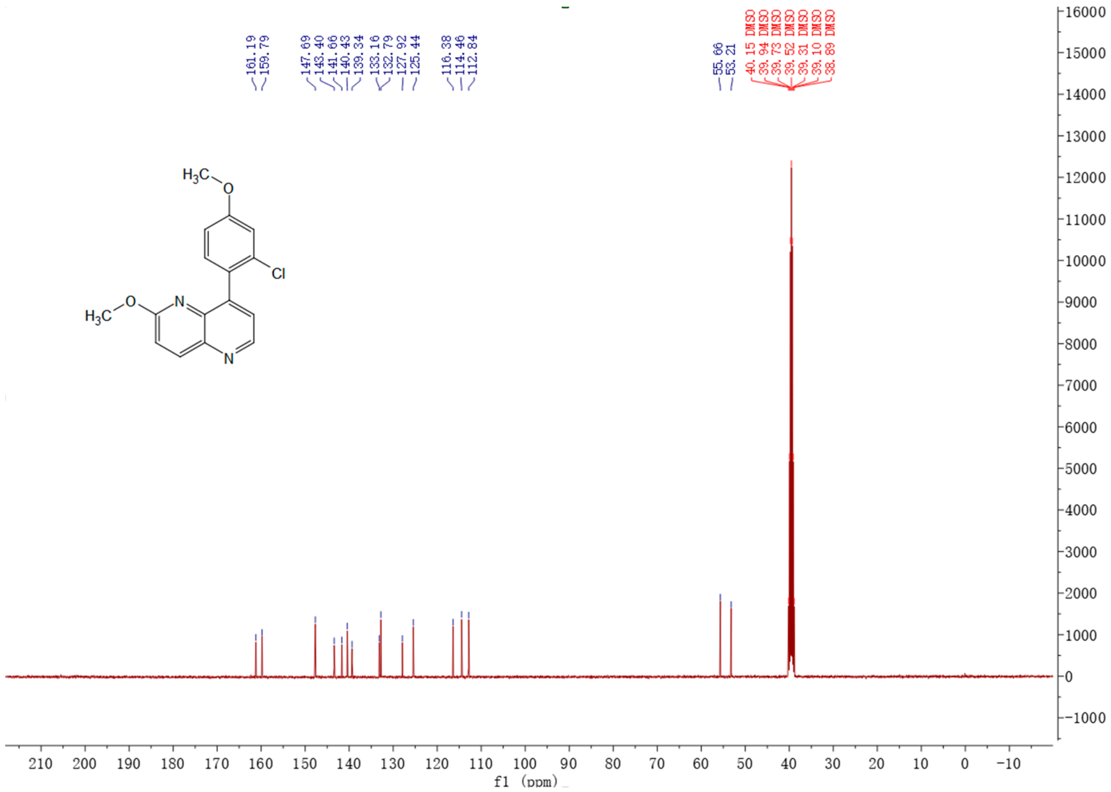

Figure S8: The ^{13}C NMR spectrum of compound **21b** in DMSO-*d6* (100 MHz).

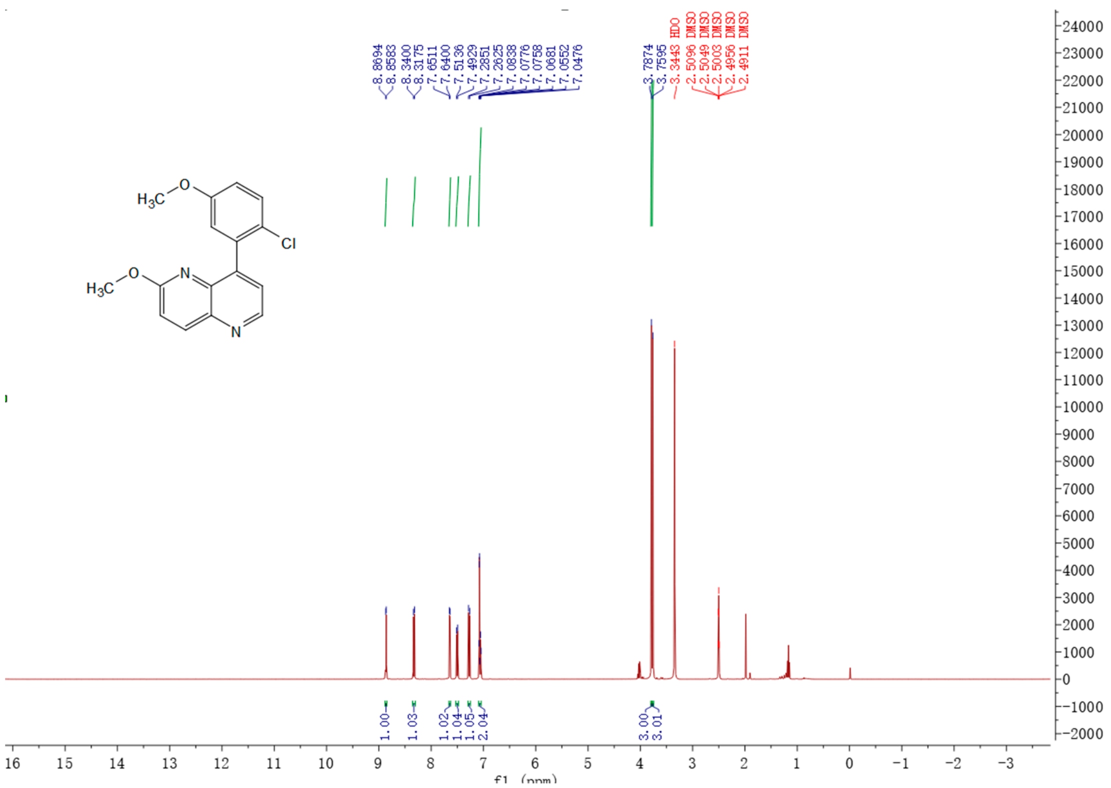

Figure S9: The ^{1}H NMR spectrum of compound **21c** in DMSO-*d6* (400 MHz).

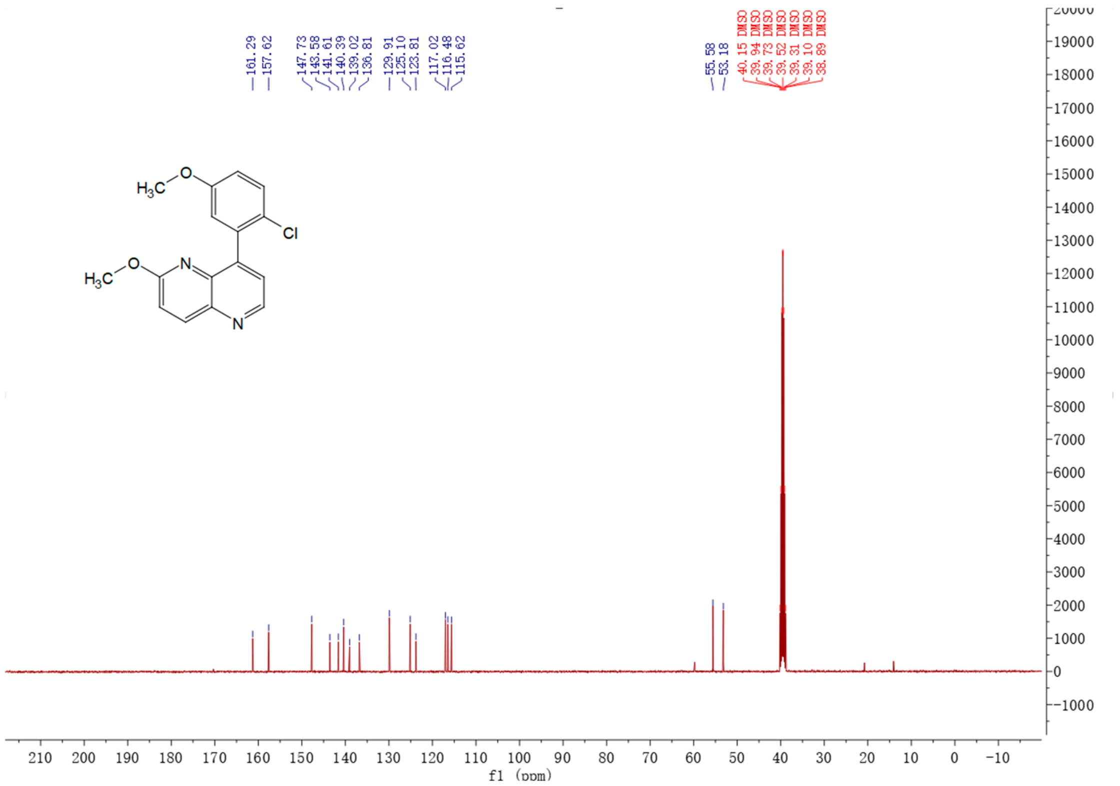

Figure S10: The ^{13}C NMR spectrum of compound **21c** in DMSO-*d6* (100 MHz).

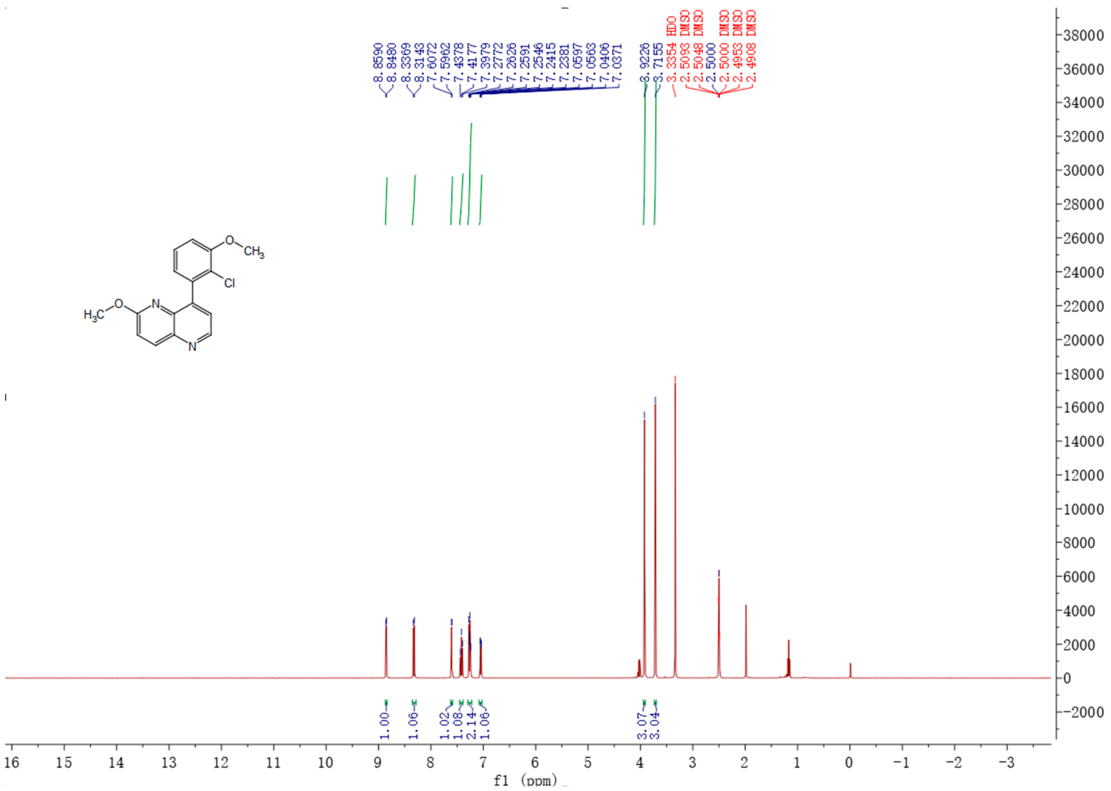

Figure S11: The ^{1}H NMR spectrum of compound **21d** in DMSO-*d6* (400 MHz).

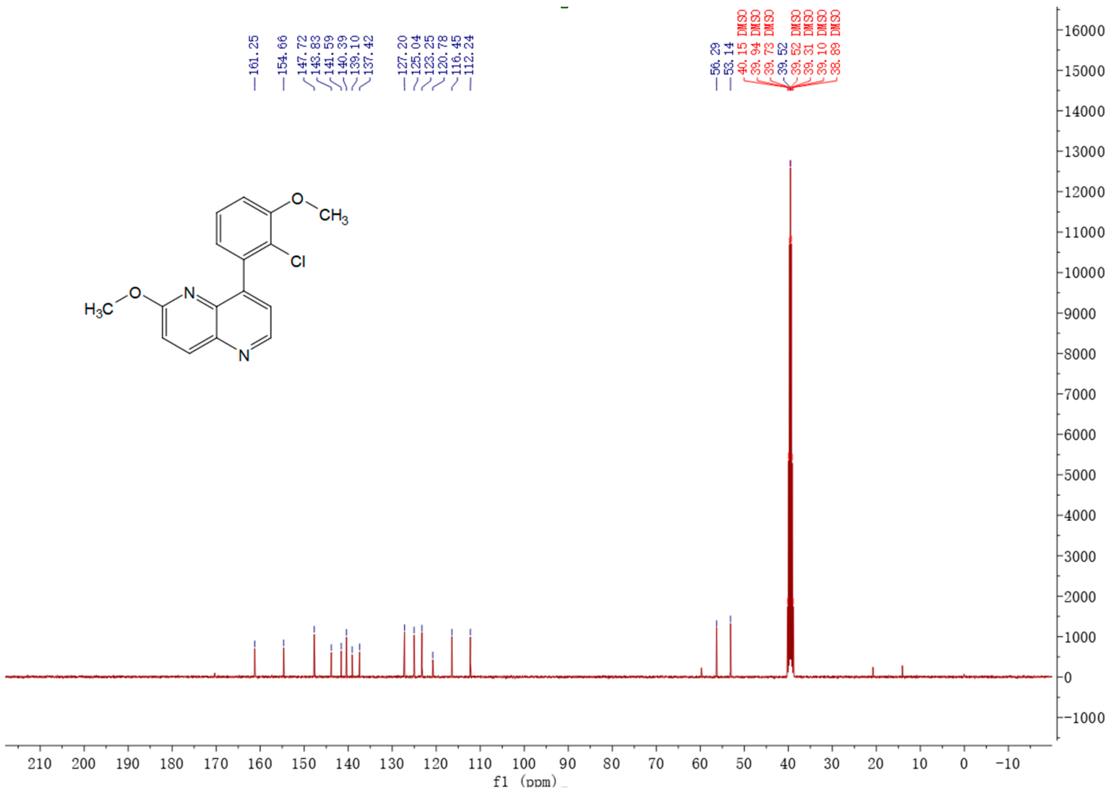

Figure S12: The ^{13}C NMR spectrum of compound **21d** in DMSO-*d6* (100 MHz).

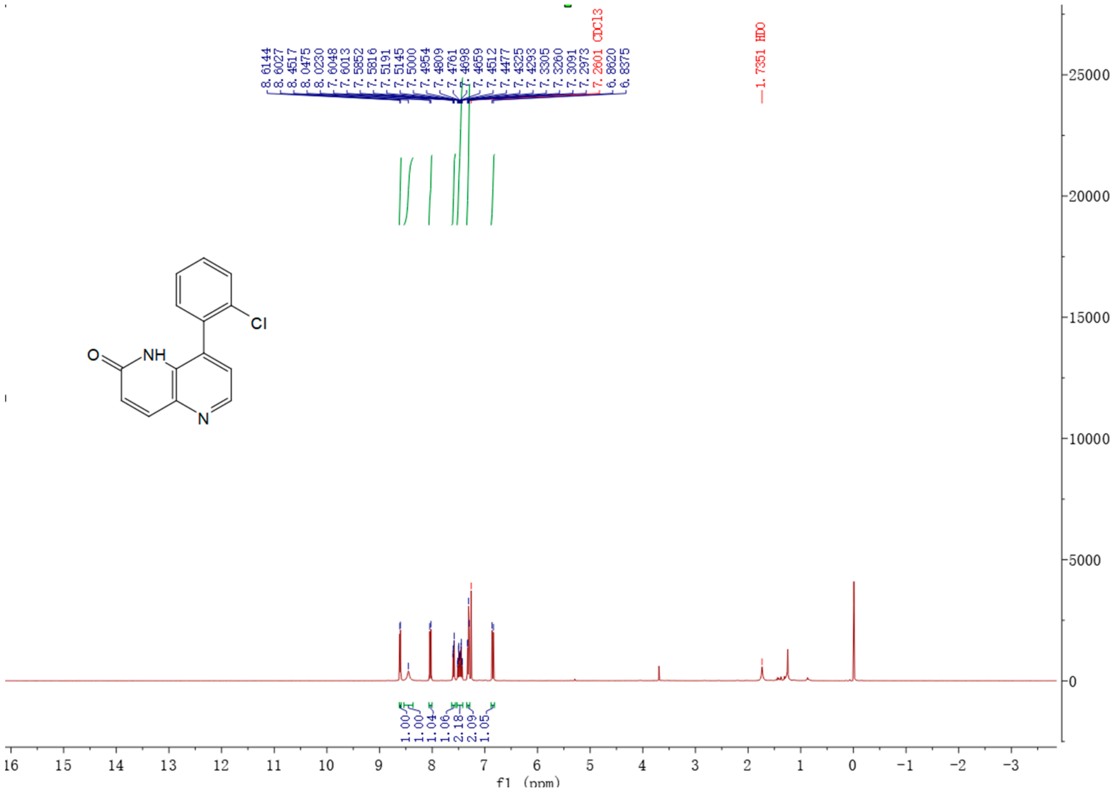

Figure S13: The ^{1}H NMR spectrum of compound **22a** in $CDCl_3$ (400 MHz).

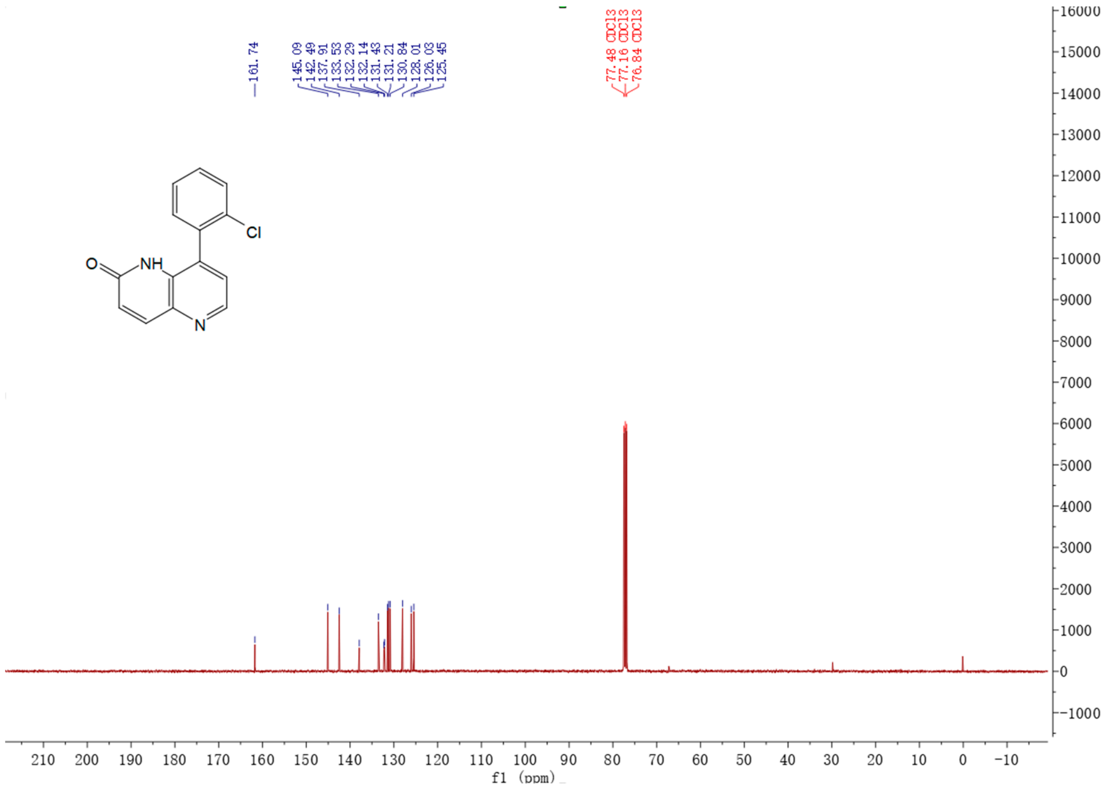

Figure S14: The ^{13}C NMR spectrum of compound **22a** in $CDCl_3$ (100 MHz).

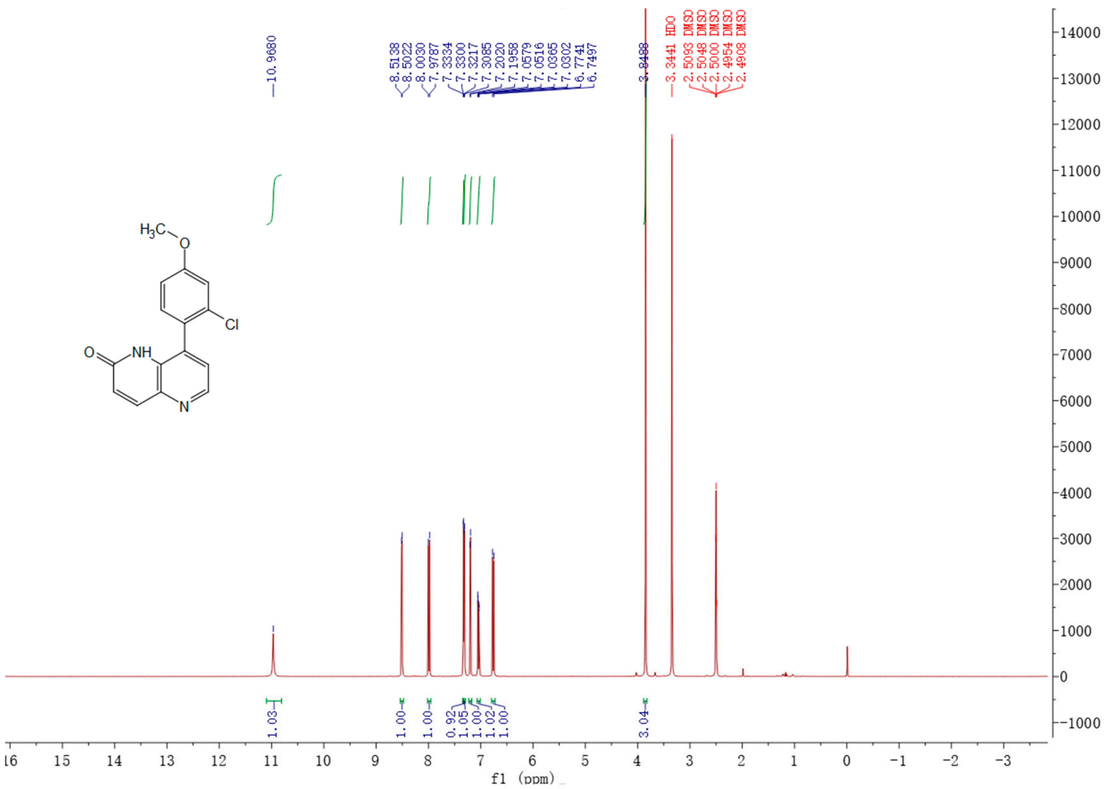

Figure S15: The ^{1}H NMR spectrum of compound **22b** in DMSO-*d6* (400 MHz).

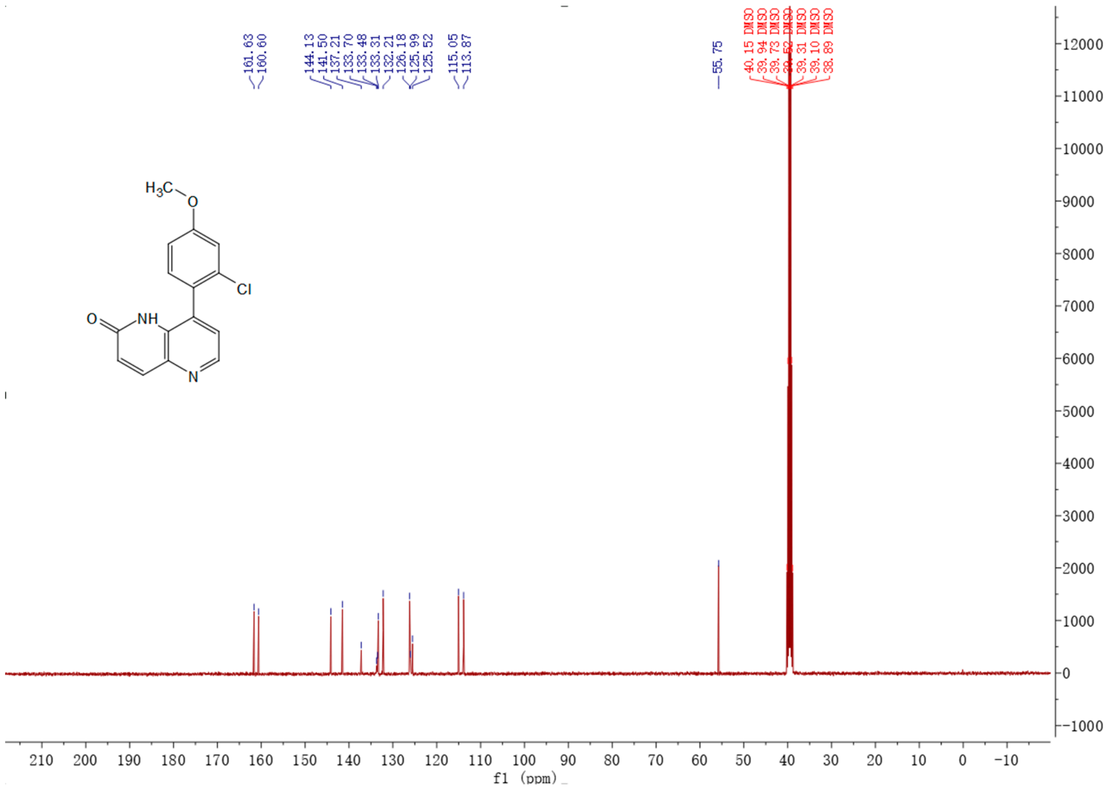

Figure S16: The ^{13}C NMR spectrum of compound **22b** in DMSO-*d6* (100 MHz).

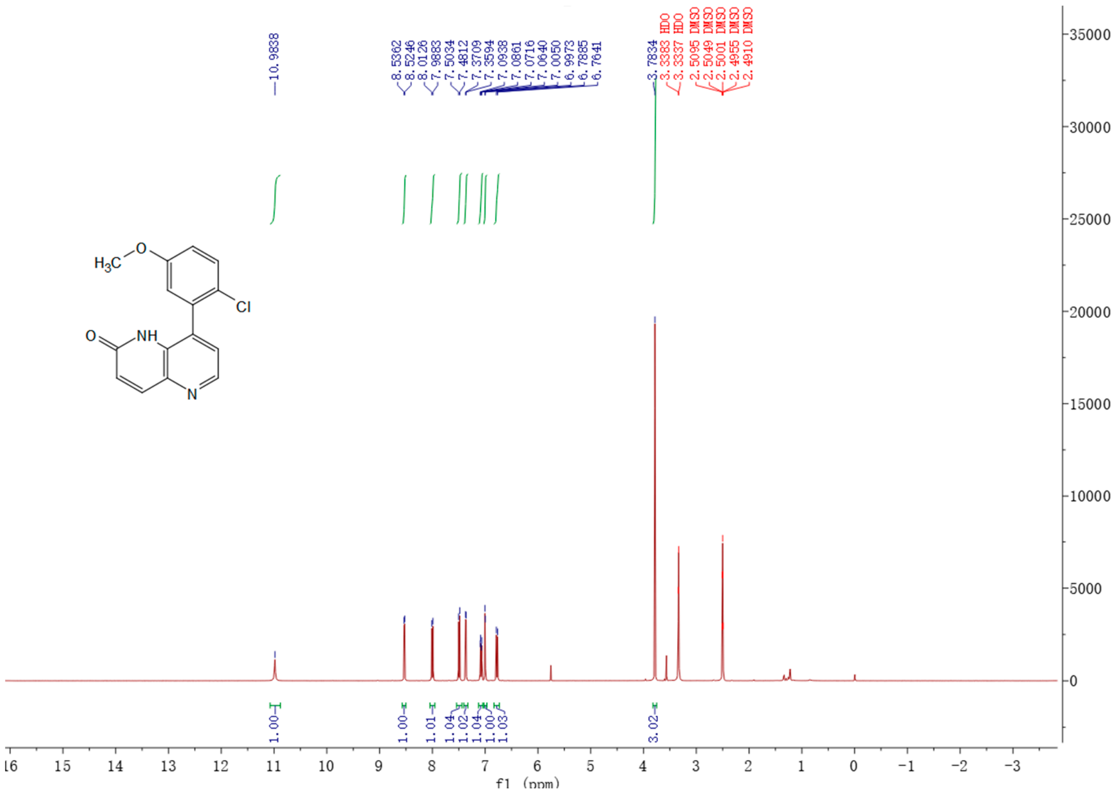

Figure S17: The ^{1}H NMR spectrum of compound **22c** in DMSO-*d6* (400 MHz).

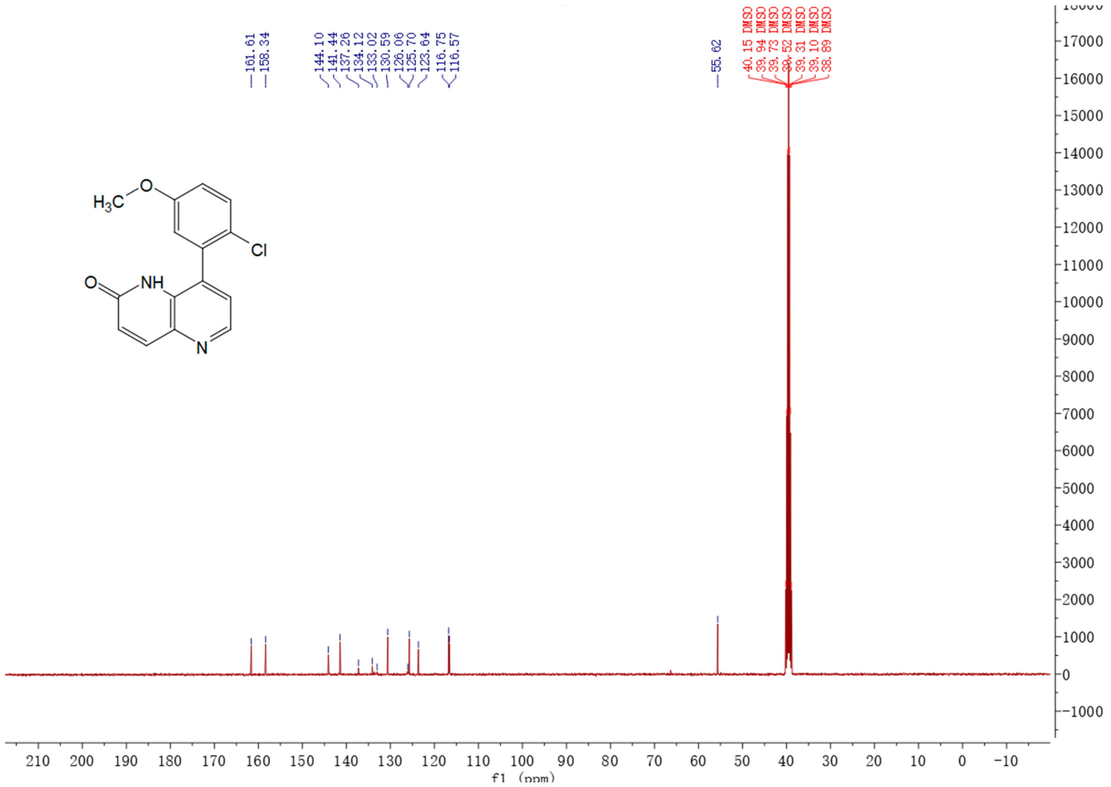

Figure S18: The ^{13}C NMR spectrum of compound **22c** in DMSO-*d6* (100 MHz).

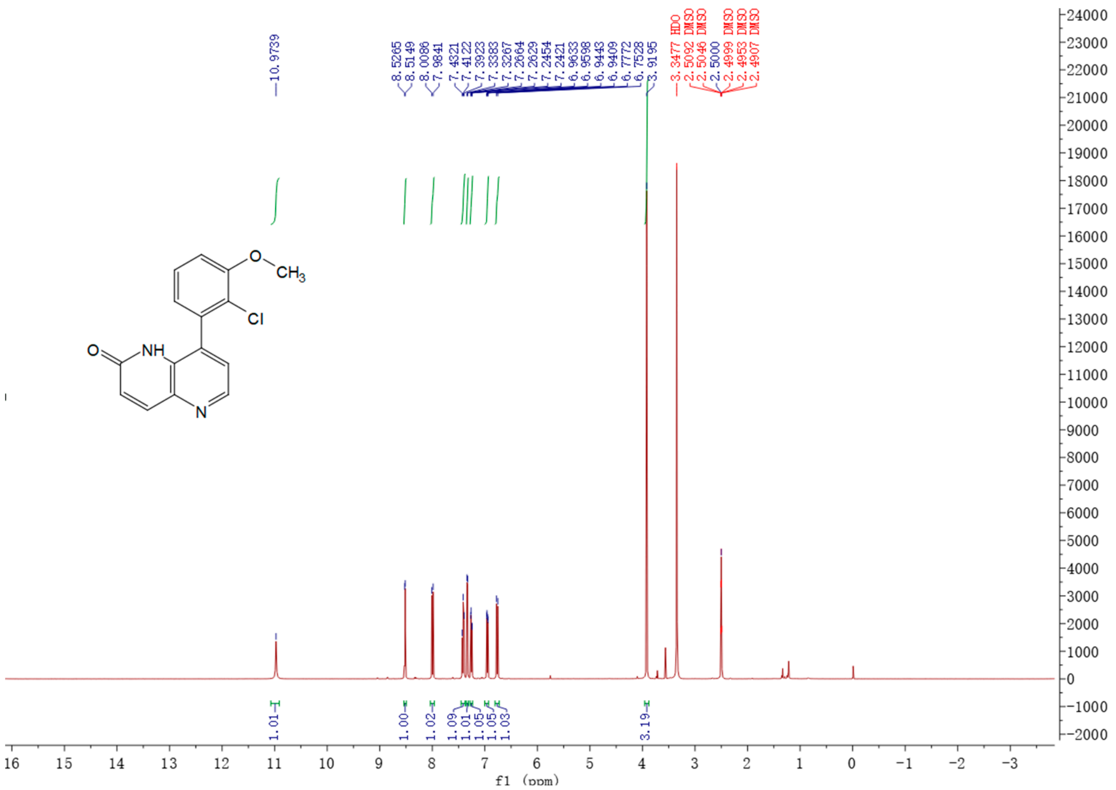

Figure S19: The ^{1}H NMR spectrum of compound **22d** in DMSO-*d6* (400 MHz).

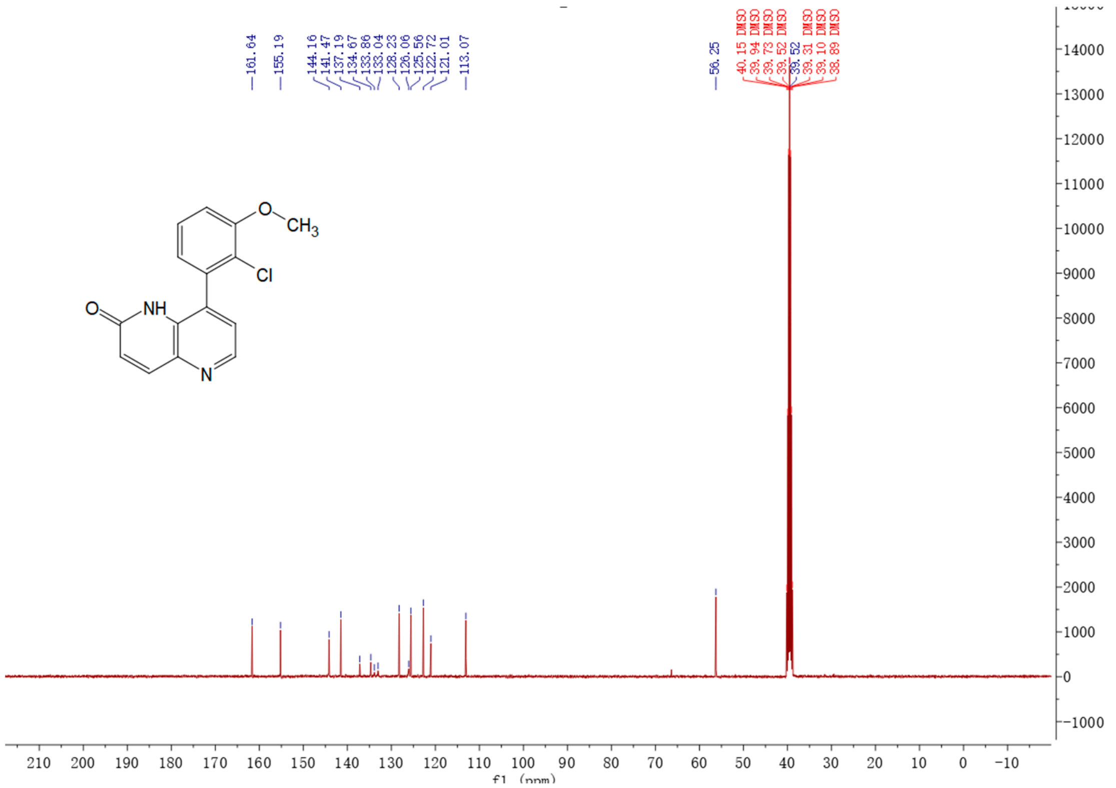

Figure S20: The ^{13}C NMR spectrum of compound **22d** in DMSO-*d6* (100 MHz).

C14H8N2O (220.06)

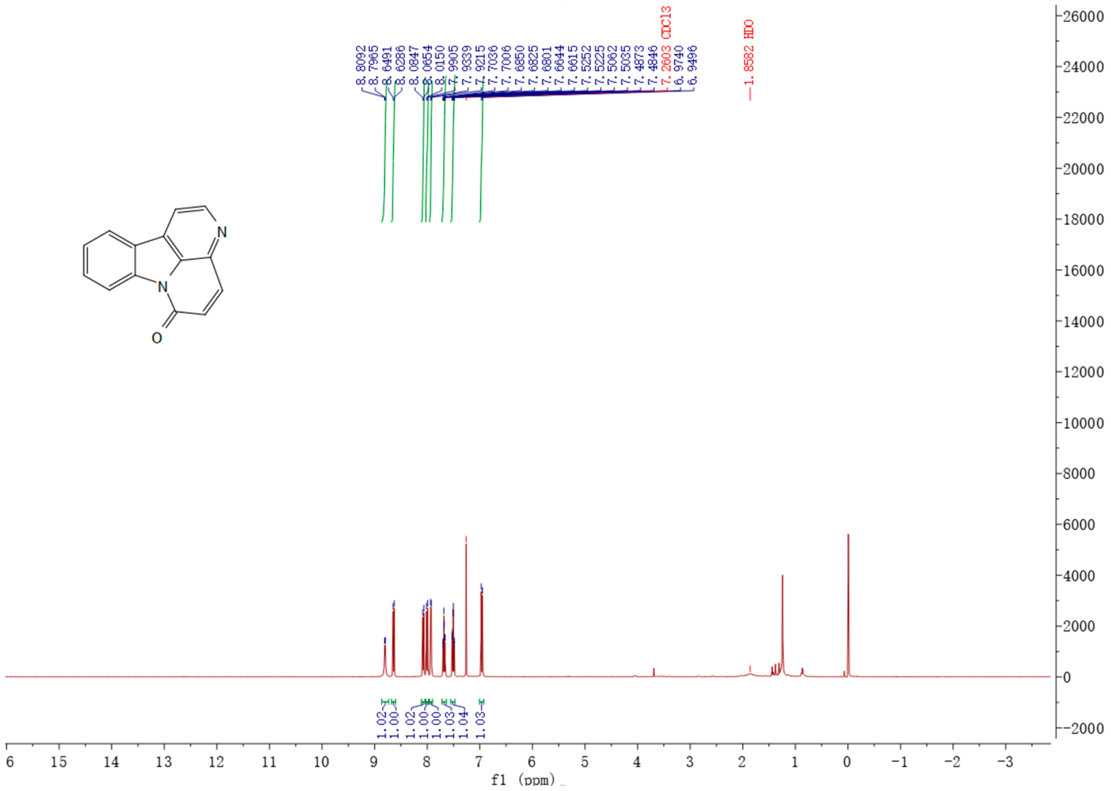

Figure S21: The ^{1}H NMR spectrum of compound **1** in $CDCl_3$ (400 MHz).

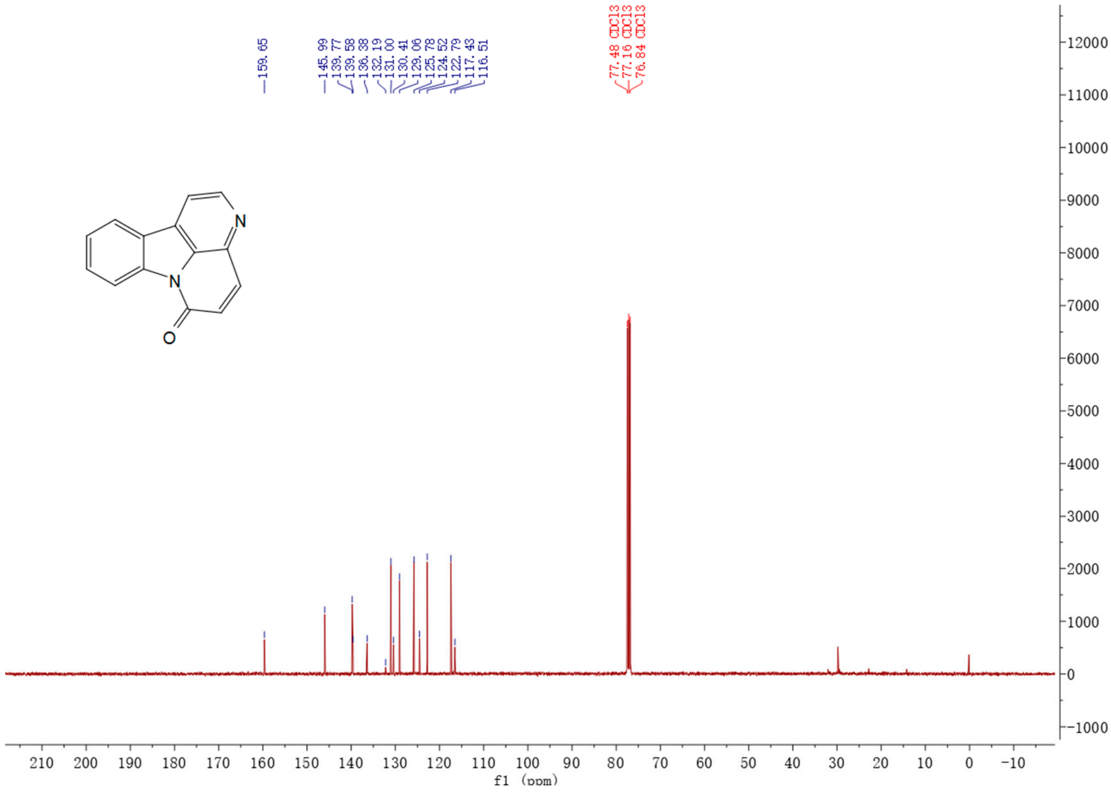

Figure S22: The ^{13}C NMR spectrum of compound **1** in $CDCl_3$ (100 MHz).

$C_{15}H_{10}N_2O_2$ (250.07)

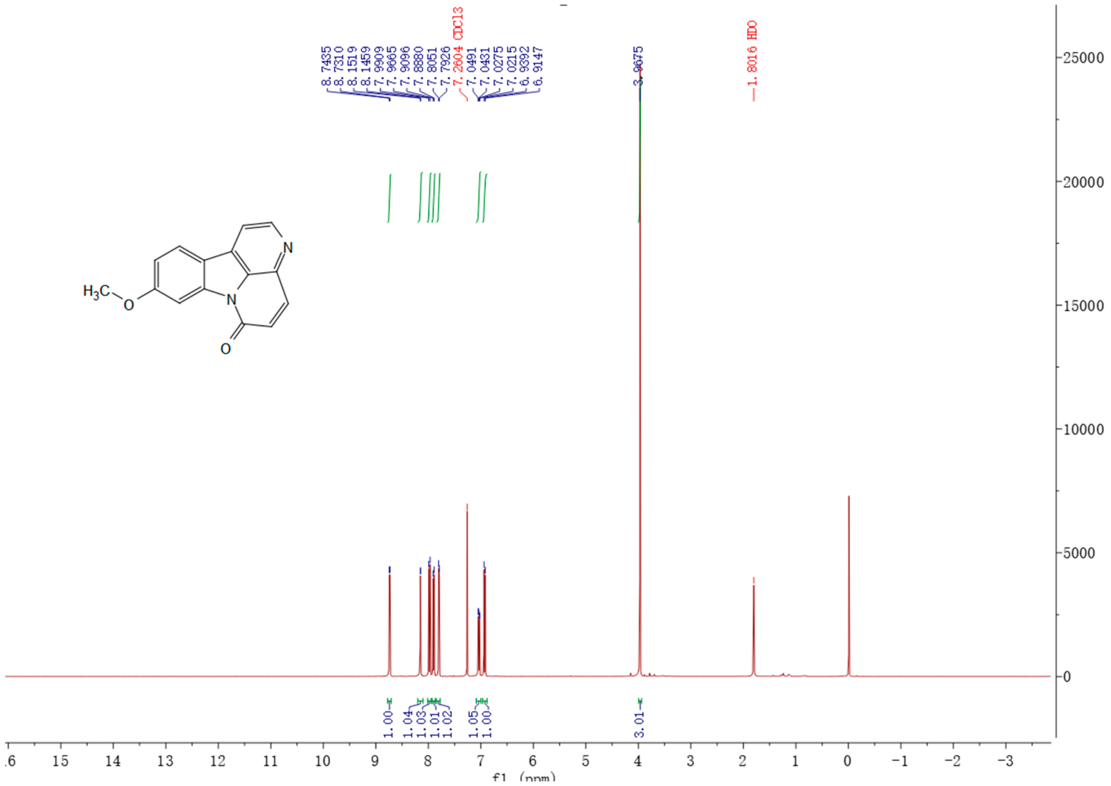

Figure S23: The ^{1}H NMR spectrum of compound **2** in $CDCl_3$ (400 MHz).

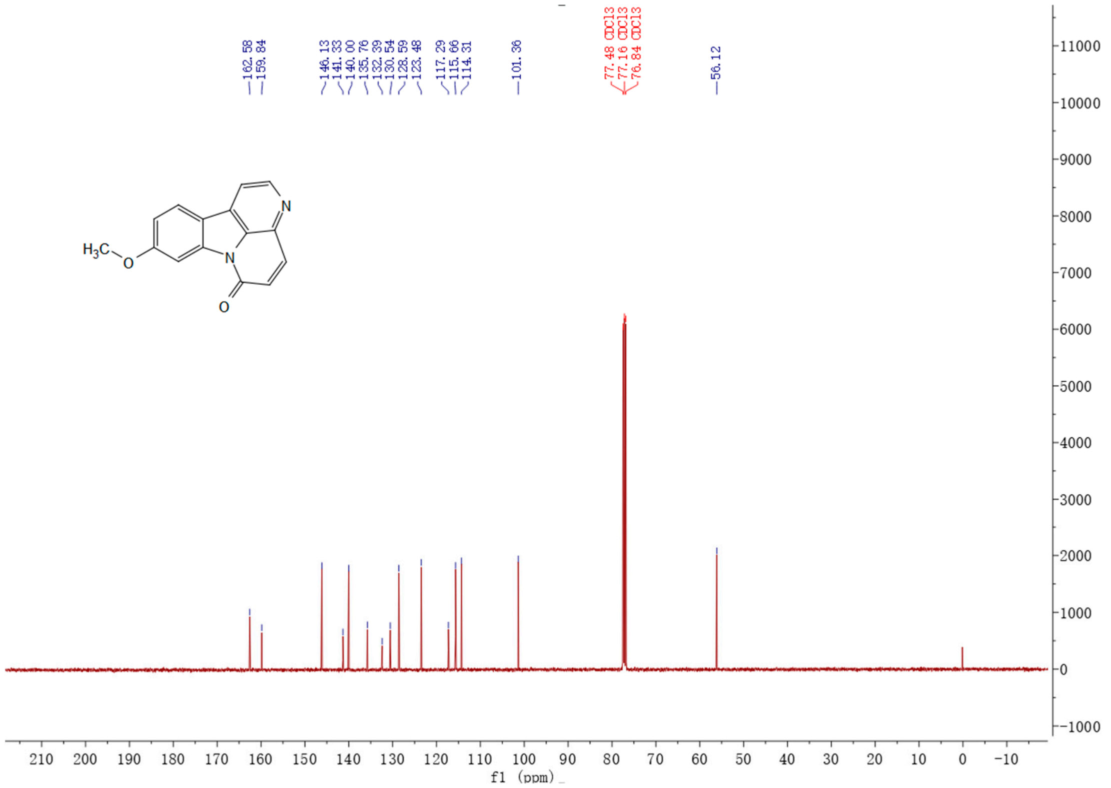

Figure S24: The ^{13}C NMR spectrum of compound **2** in $CDCl_3$ (100 MHz).

C$_{15}$H$_{10}$N$_2$O$_2$ (250.07)

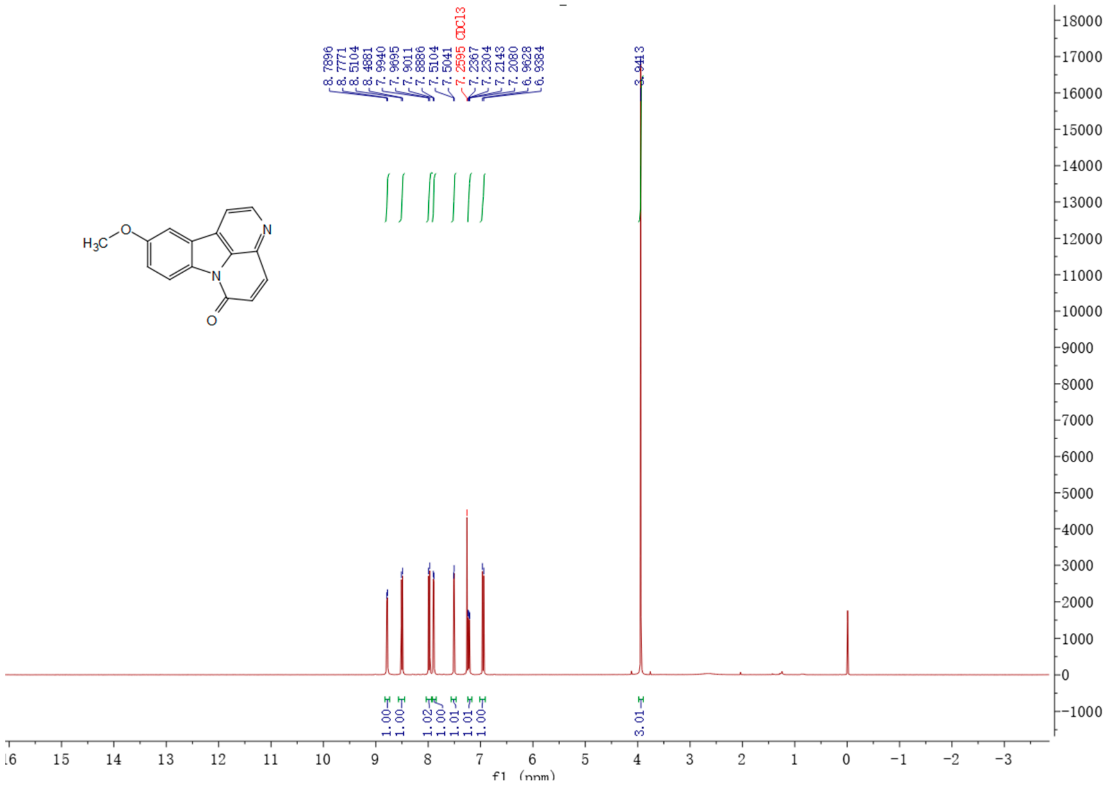

Figure S25: The ^{1}H NMR spectrum of compound **3** in CDCl$_3$ (400 MHz).

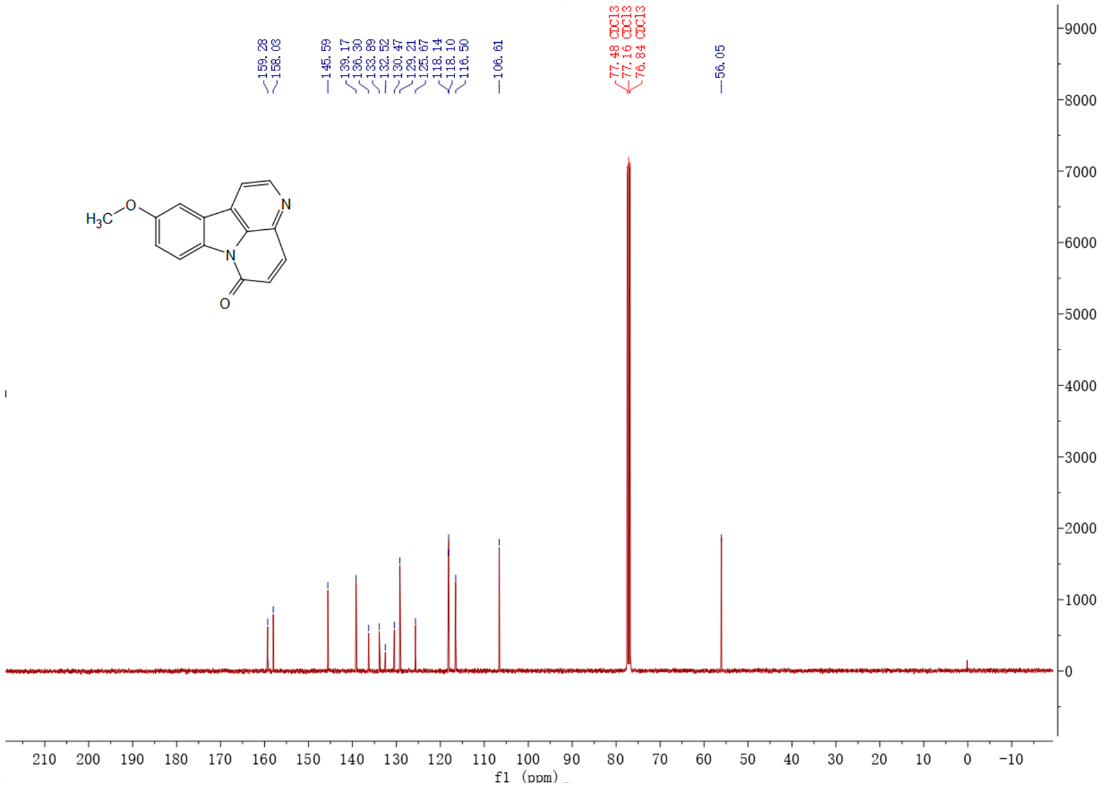

Figure S26: The ^{13}C NMR spectrum of compound **3** in CDCl$_3$ (100 MHz).

$C_{15}H_{10}N_2O_2$ (250.07)

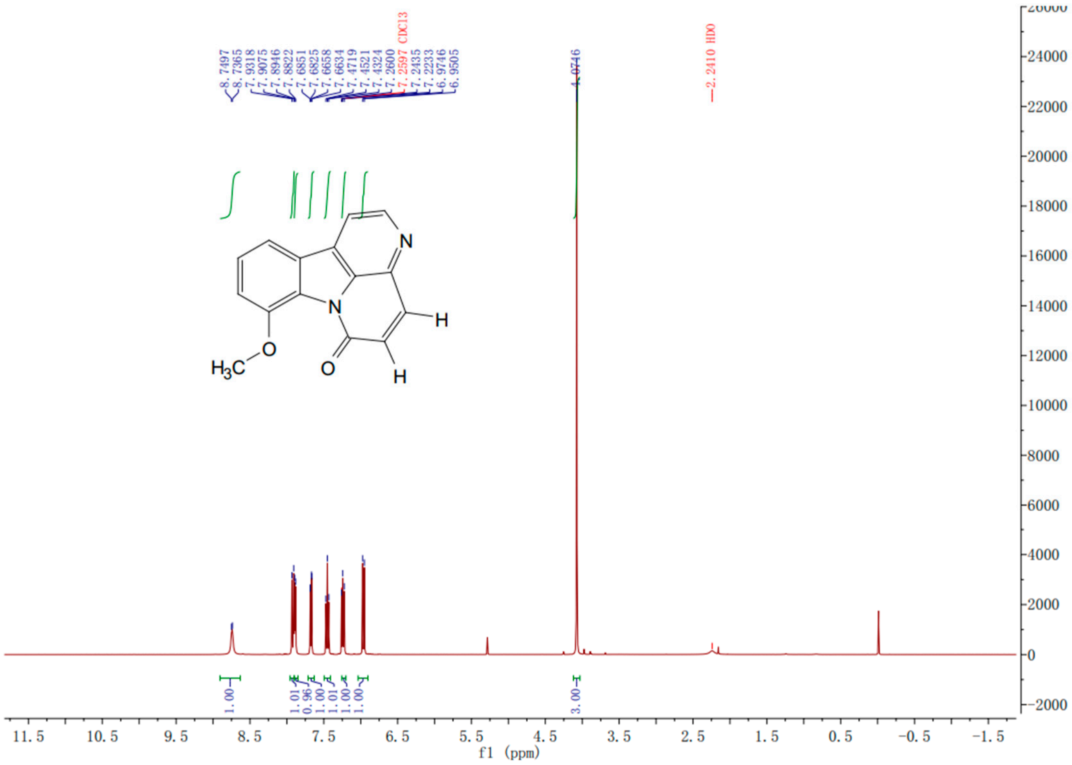

Figure S27: The ^{1}H NMR spectrum of compound **4** in $CDCl_3$ (400 MHz).

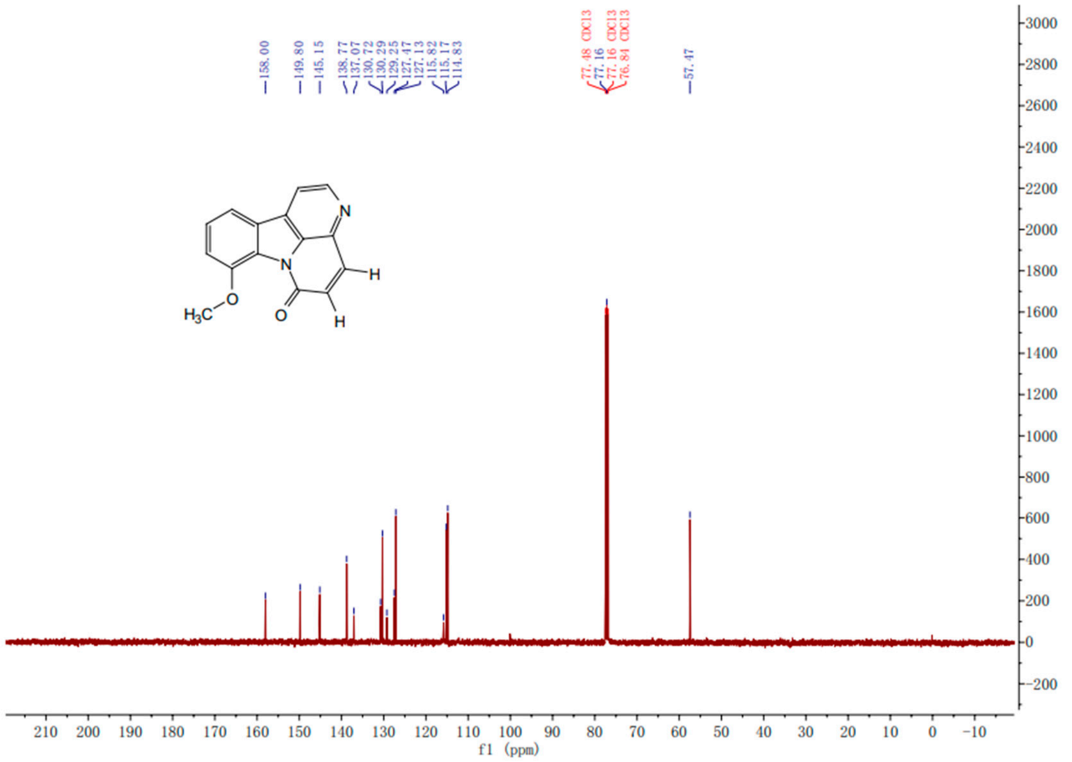

Figure S28: The ^{13}C NMR spectrum of compound **4** in $CDCl_3$ (100 MHz).

$C_{15}H_{22}O_{10}$ (362.12)

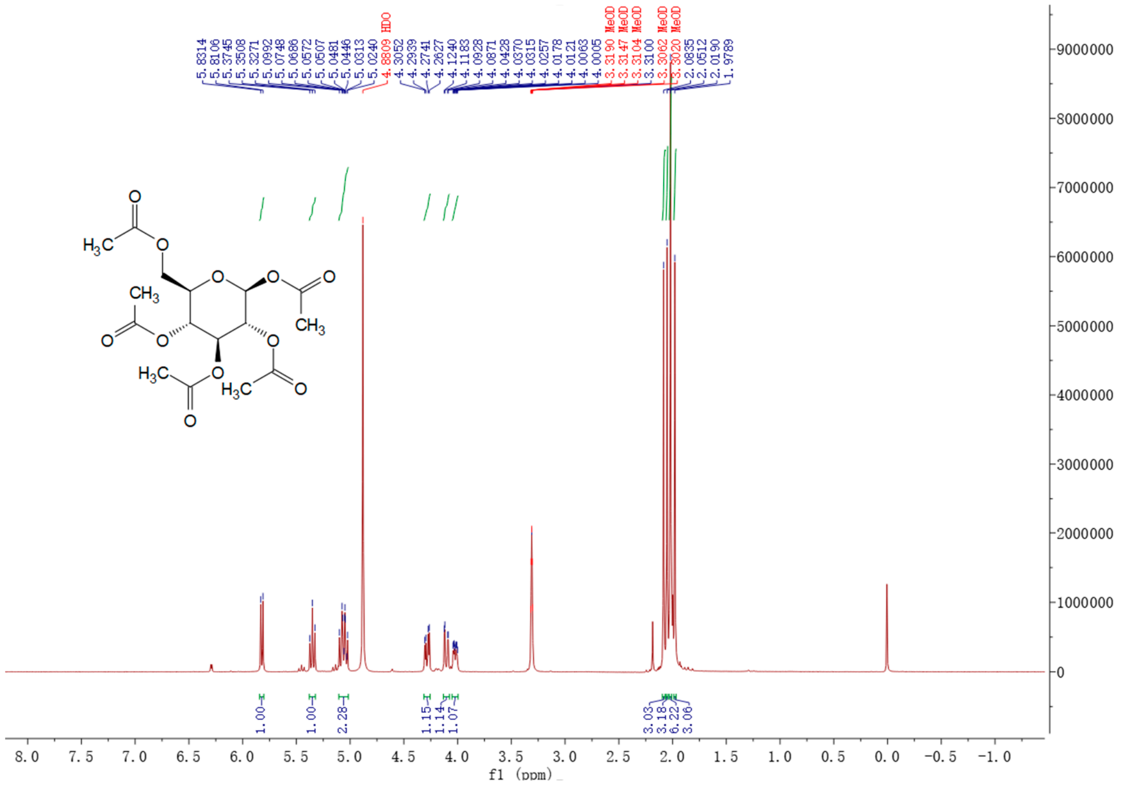

Figure S29: The ^1H NMR spectrum of compound **25** in MeOD (400 MHz).

$C_{14}H_{19}BrO_{9}$ (410.02)

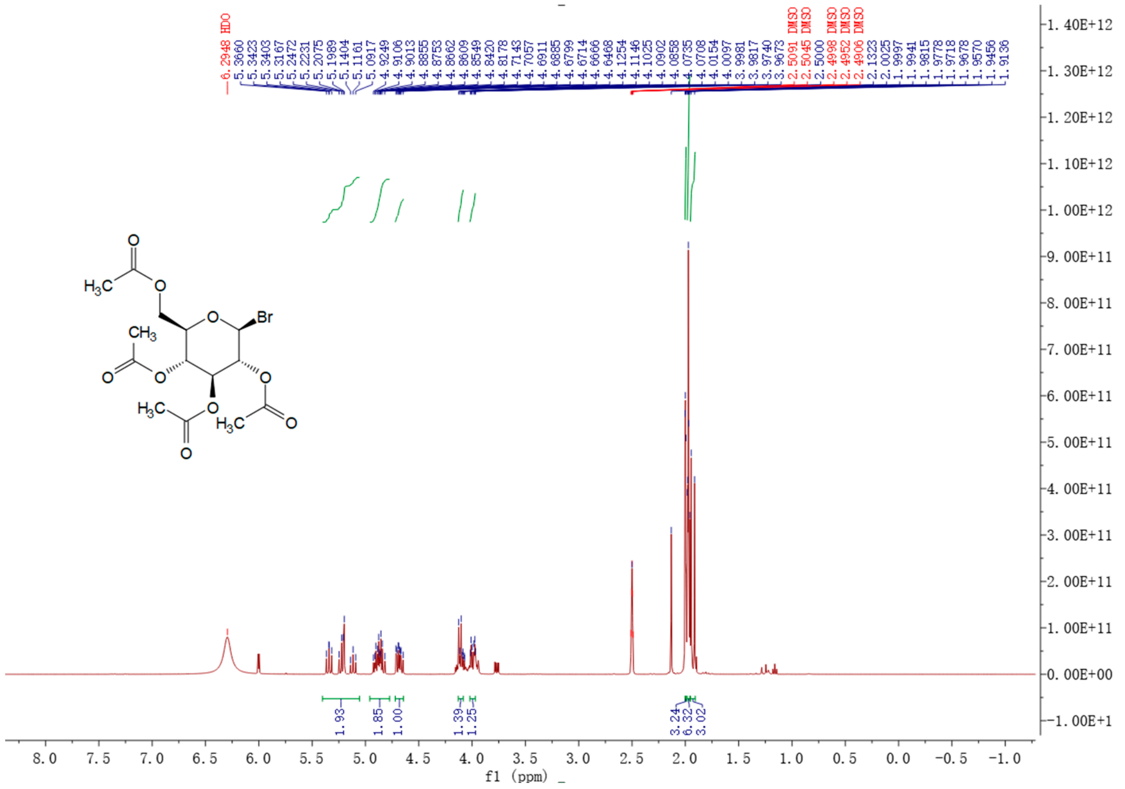

Figure S30: The ^1H NMR spectrum of compound **27** in DMSO-*d6* (400 MHz).

C26H35BrO17 (698.11)

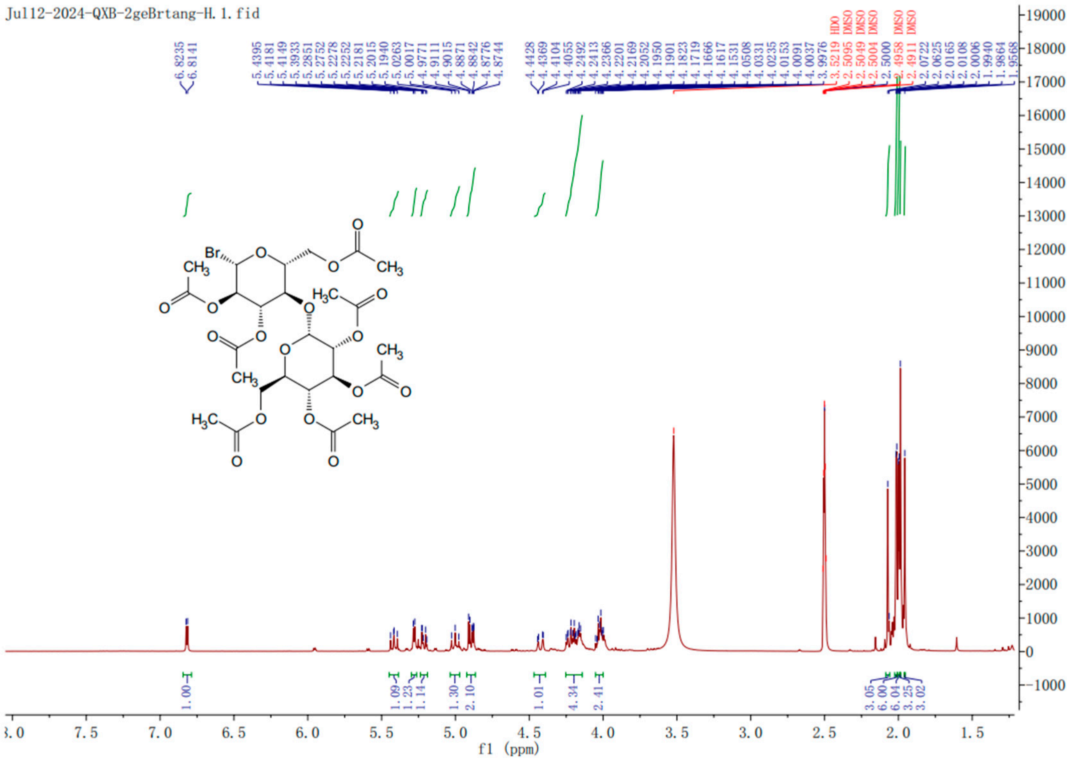

Figure S31: The ^{1}H NMR spectrum of compound **28** in DMSO-*d6* (400 MHz).

C14H8N2O2 (236.06)

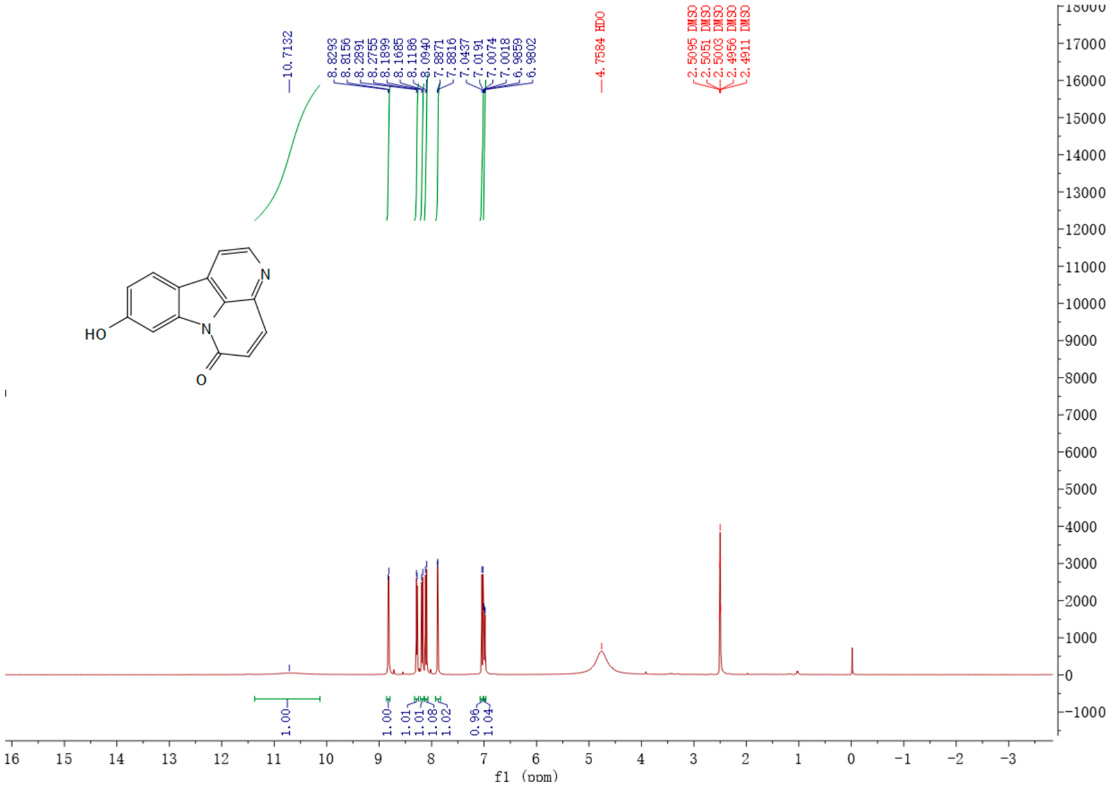

Figure S32: The ^{1}H NMR spectrum of compound **5** in DMSO-*d6* (400 MHz).

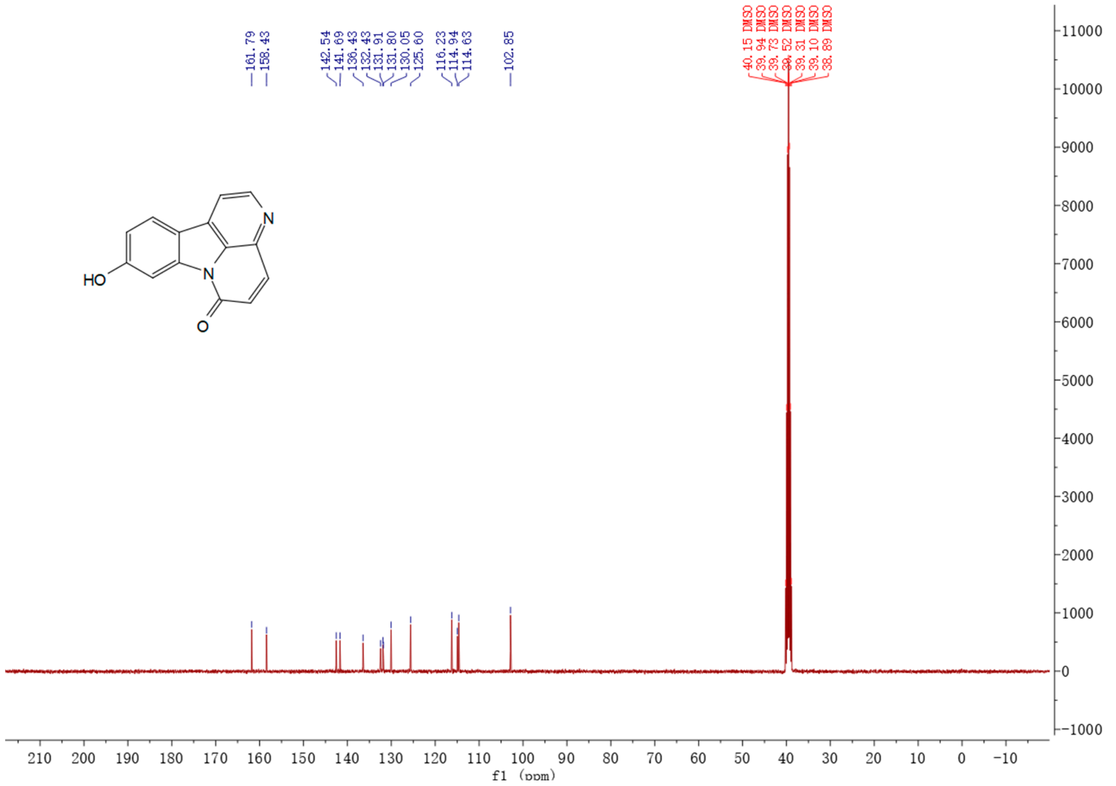

Figure S33: The ^{13}C NMR spectrum of compound **5** in DMSO-*d6* (100 MHz).

$C_{14}H_8N_2O_2$ (236.06)

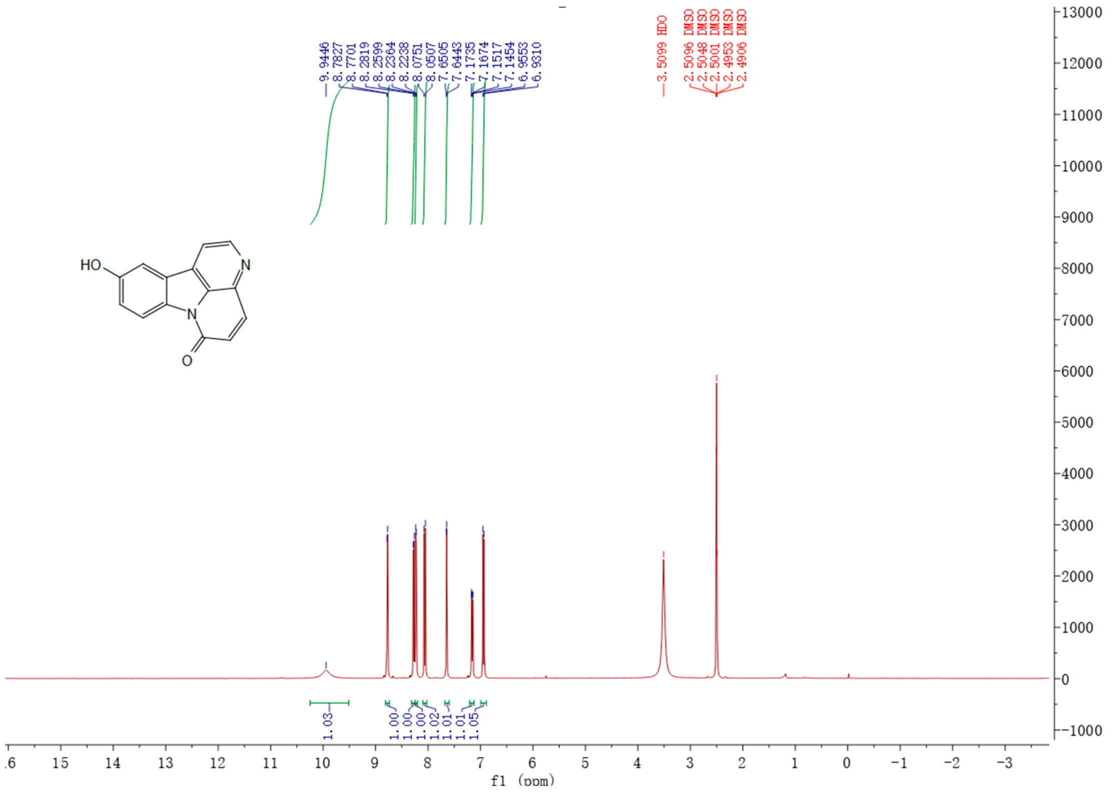

Figure S34: The ^{1}H NMR spectrum of compound **6** in DMSO-*d6* (400 MHz).

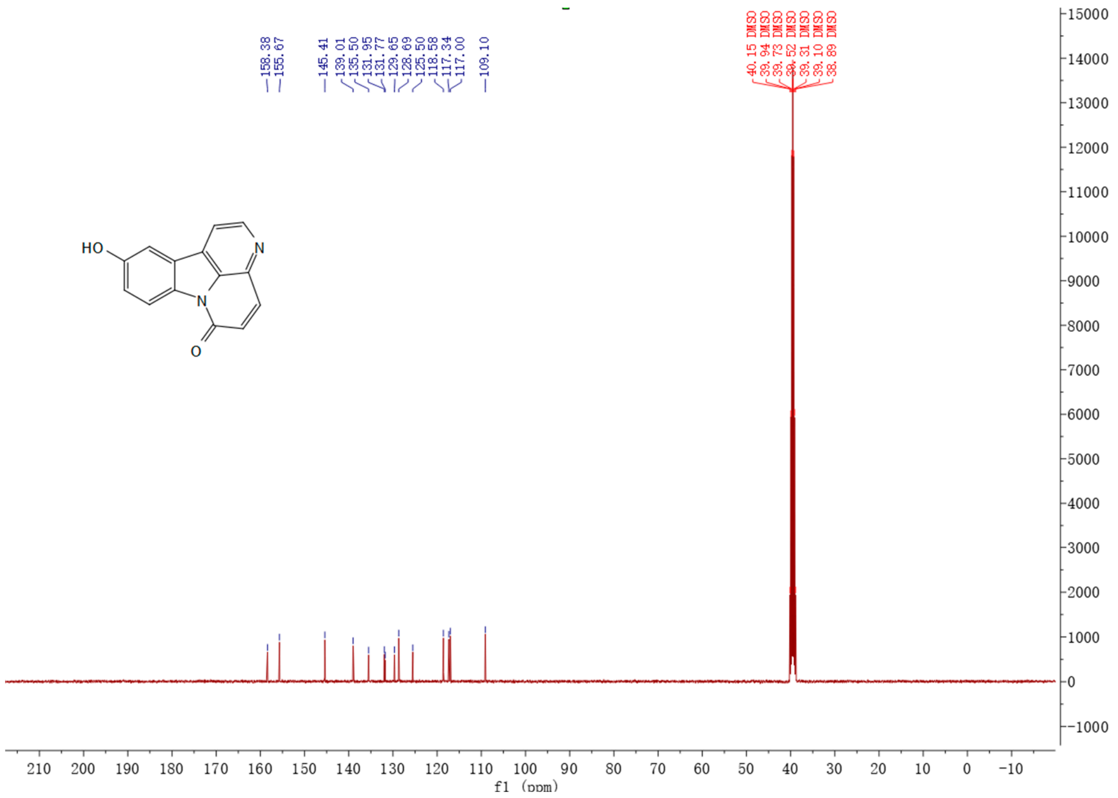

Figure S35: The ^{13}C NMR spectrum of compound **6** in DMSO-*d6* (100 MHz).

C14H8N2O2 (236.06)

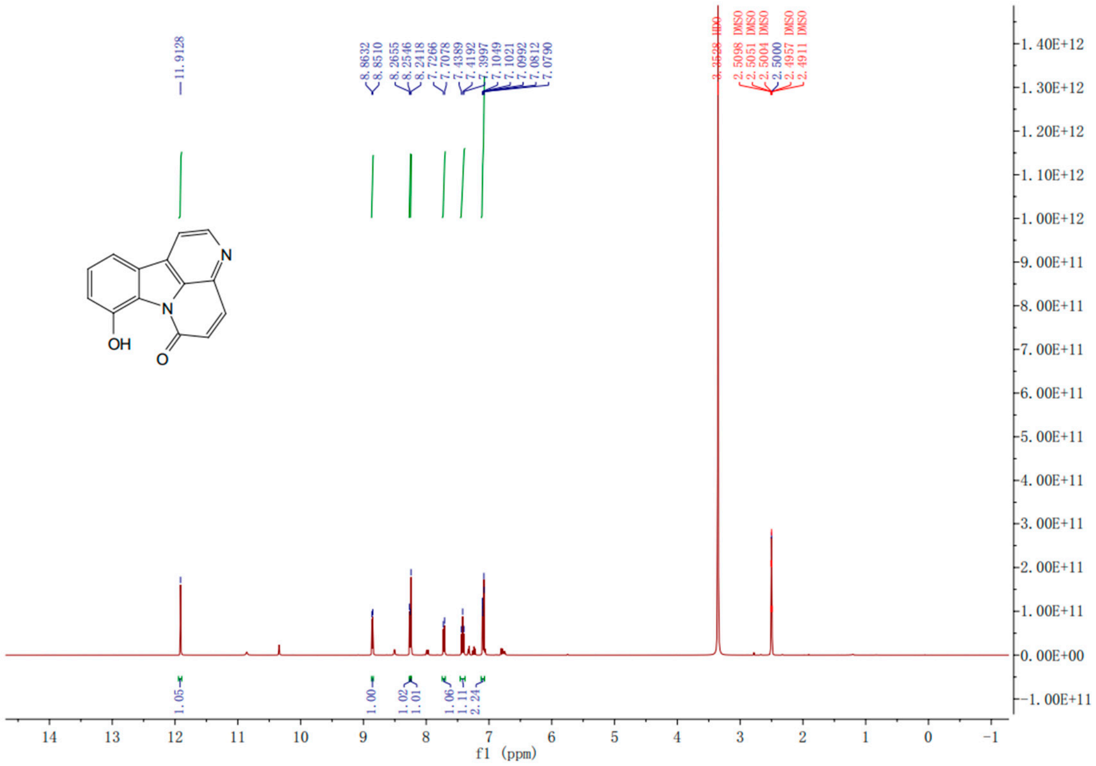

Figure S36: The ^{1}H NMR spectrum of compound **7** in DMSO-*d6* (400 MHz).

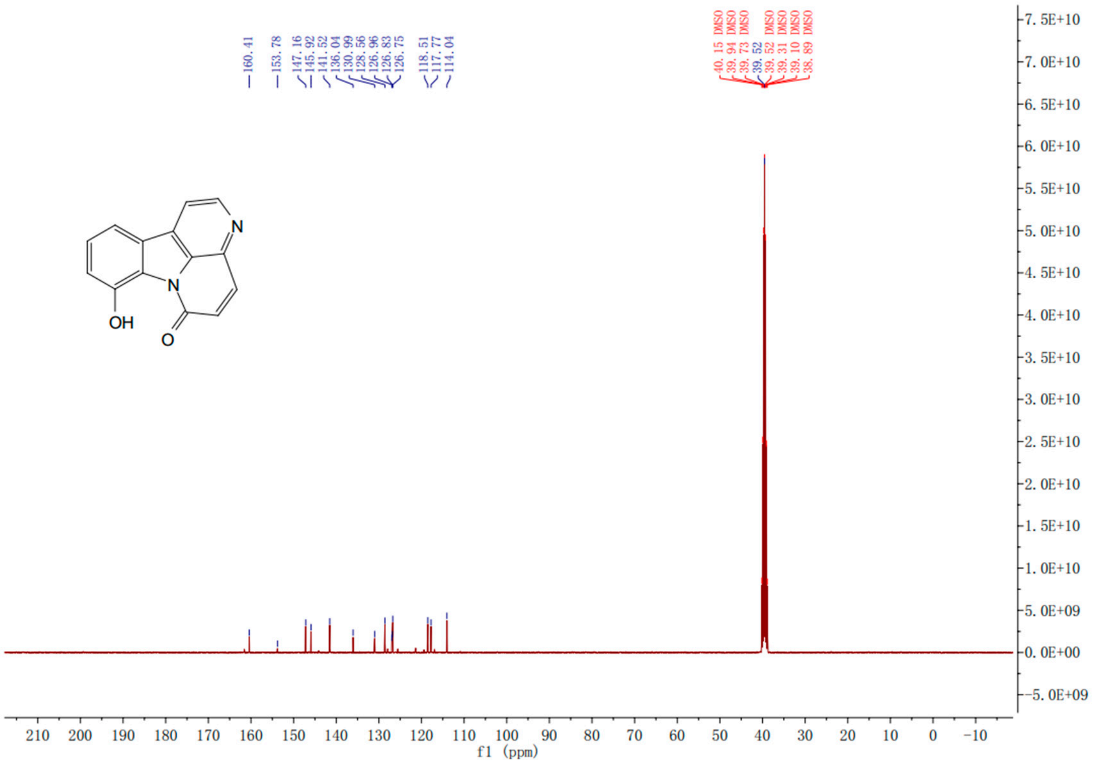

Figure S37: The ^{13}C NMR spectrum of compound **7** in DMSO-*d6* (100 MHz).

$C_{28}H_{26}N_2O_{11}$ (566.15)

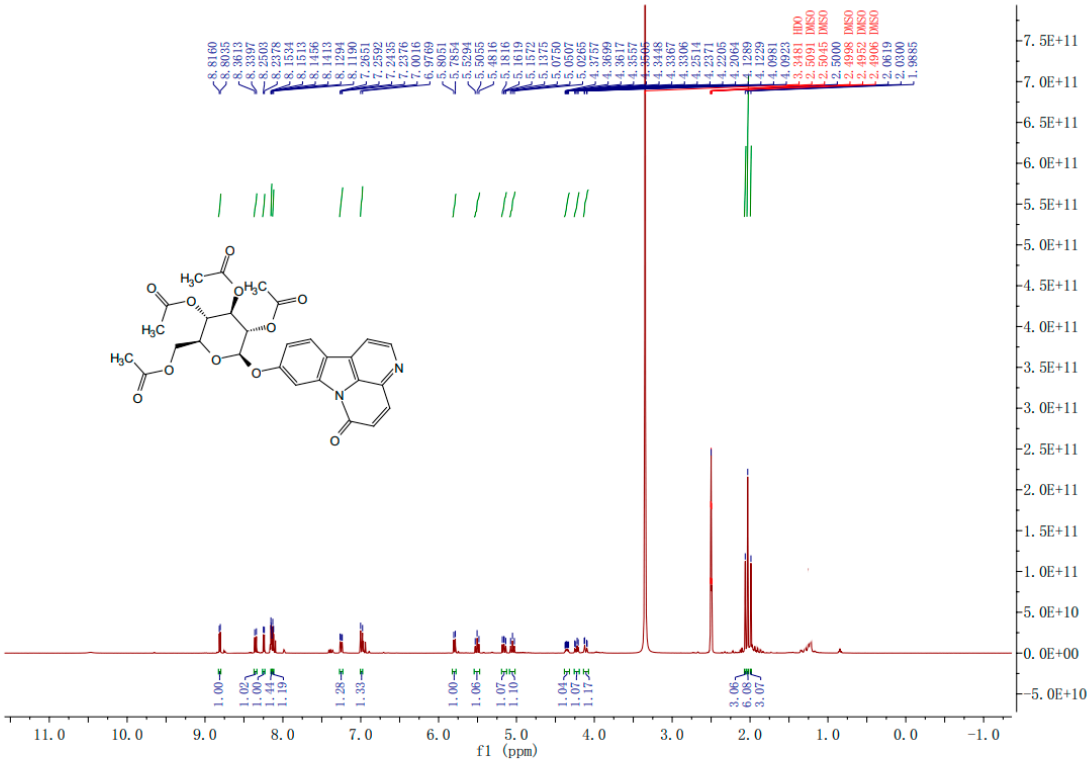

Figure S38: The ^1H NMR spectrum of compound **30a** in DMSO-*d6* (400 MHz).

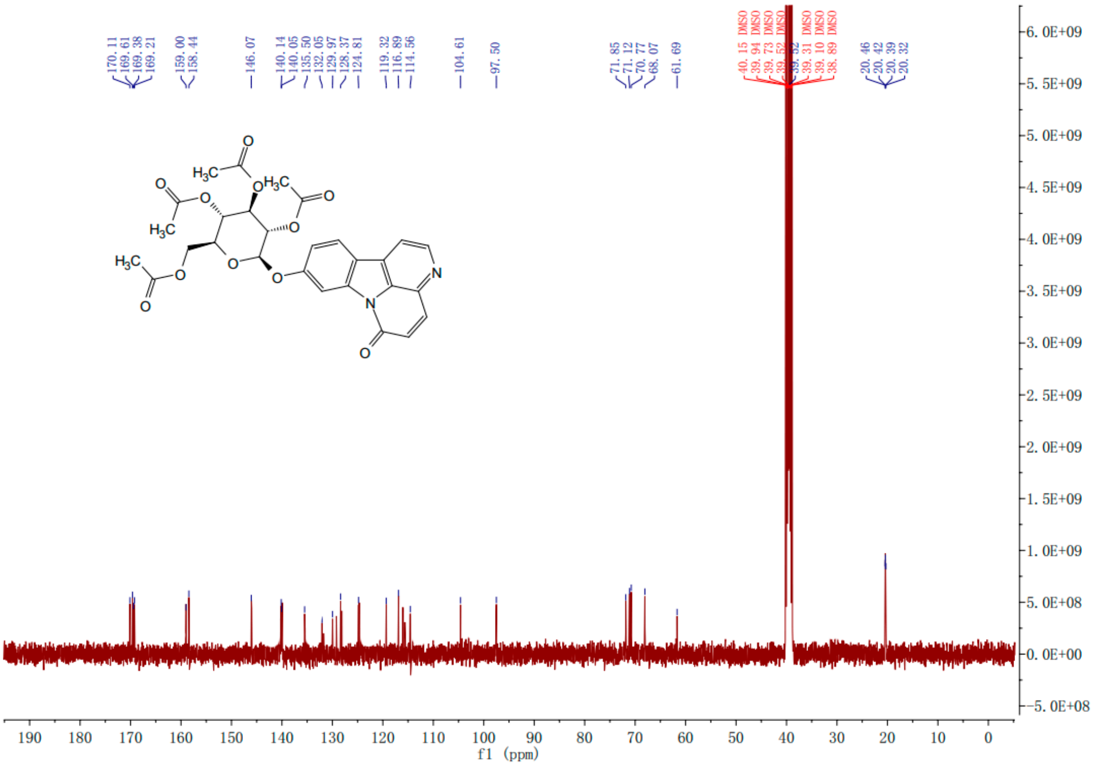

Figure S39: The ^{13}C NMR spectrum of compound **30a** in DMSO-*d6* (100 MHz).

$C_{28}H_{26}N_2O_{11}$ (566.15)

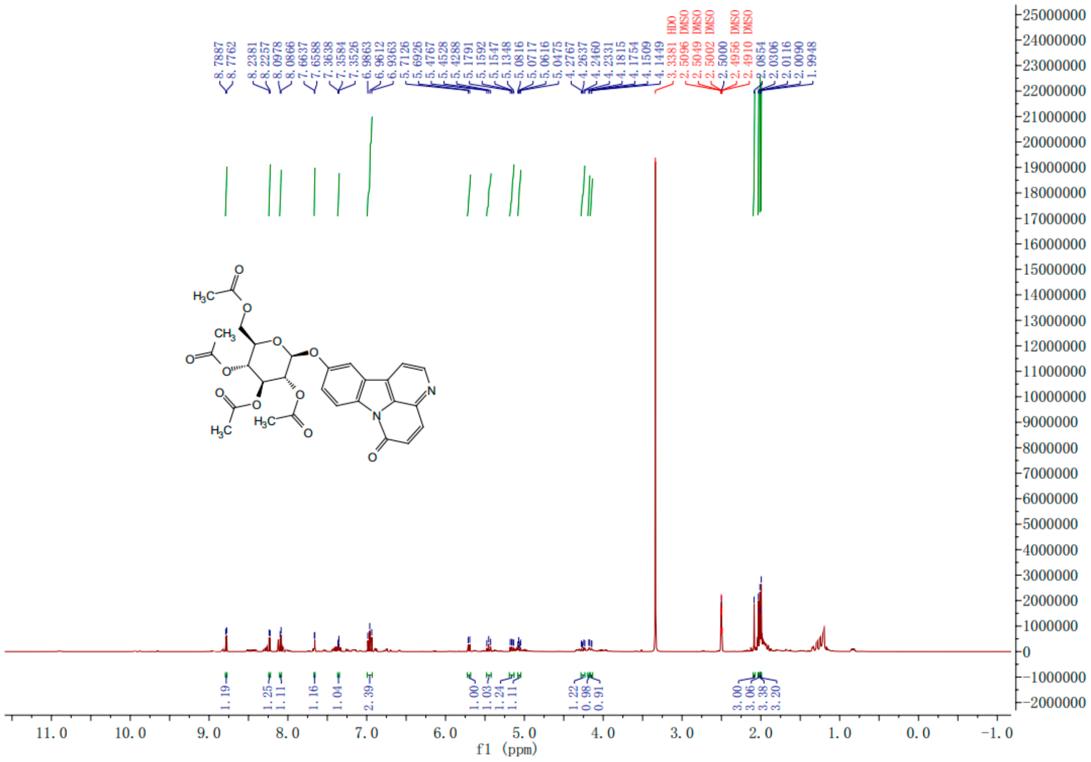

Figure S40: The ^{1}H NMR spectrum of compound **30b** in DMSO-*d6* (400 MHz).

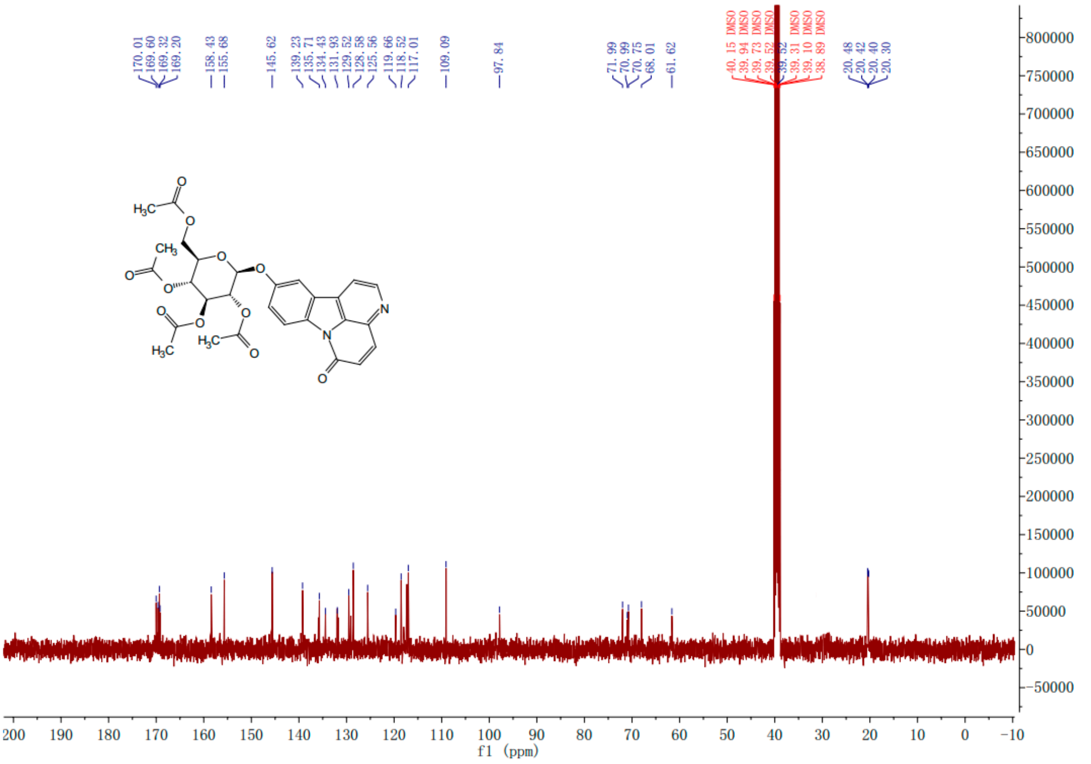

Figure S41: The ^{13}C NMR spectrum of compound **30b** in DMSO-*d6* (100 MHz).

C28H26N2O11 (566.15)

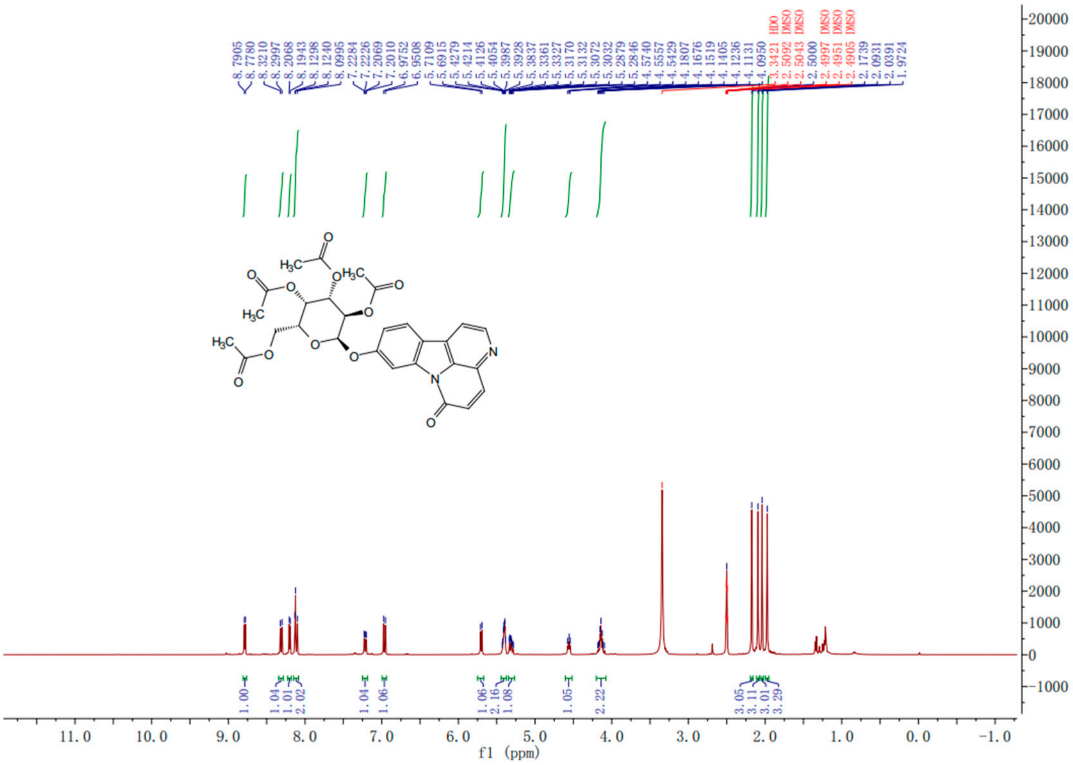

Figure S42: The ^{1}H NMR spectrum of compound **30c** in DMSO-*d6* (400 MHz).

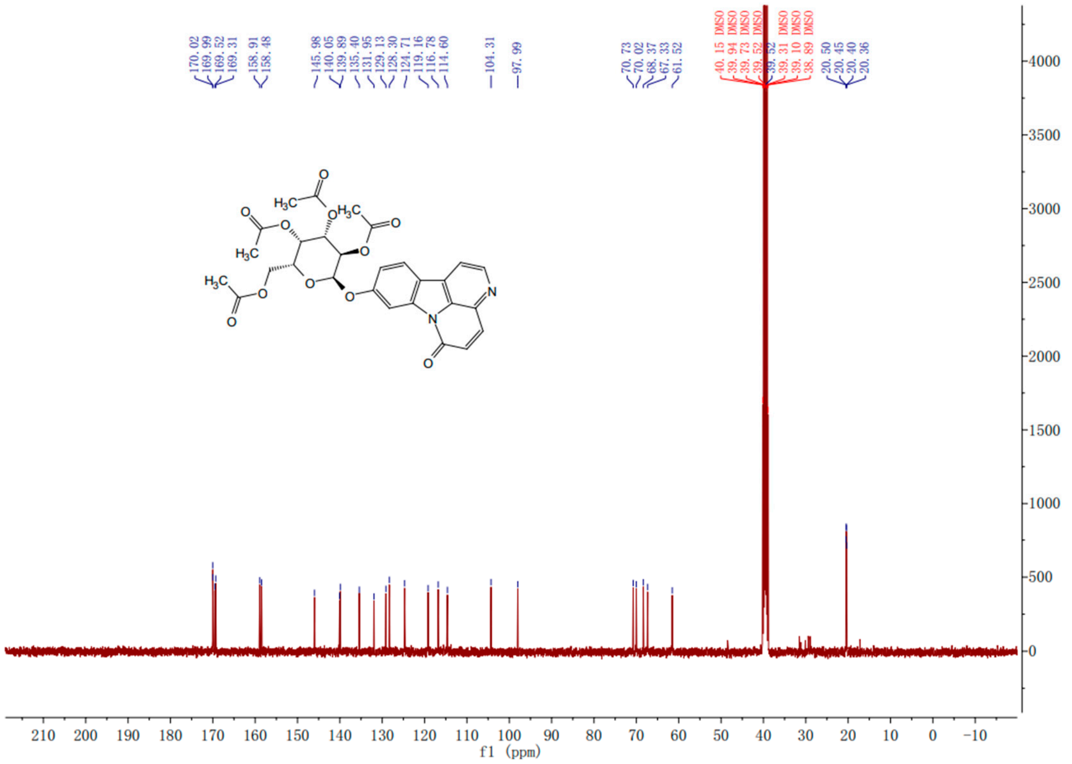

Figure S43: The ^{13}C NMR spectrum of compound **30c** in DMSO-*d6* (100 MHz).

$C_{28}H_{26}N_2O_{11}$ (566.15)

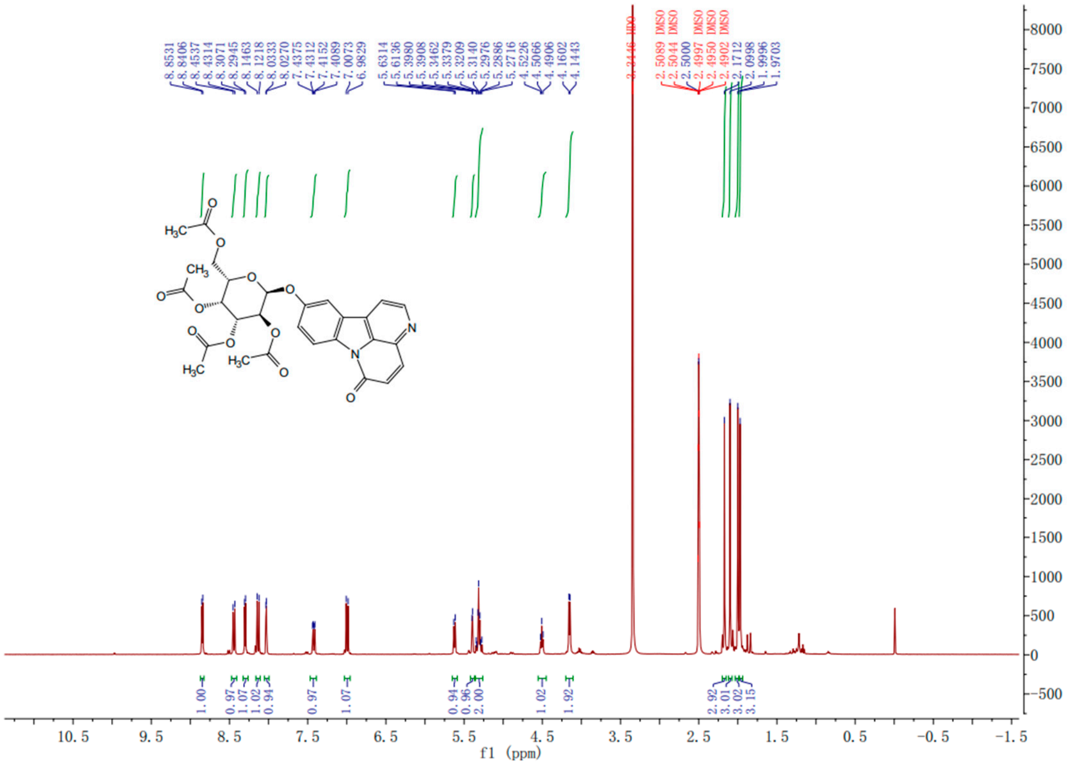

Figure S44: The ^{1}H NMR spectrum of compound **30d** in DMSO-*d6* (400 MHz).

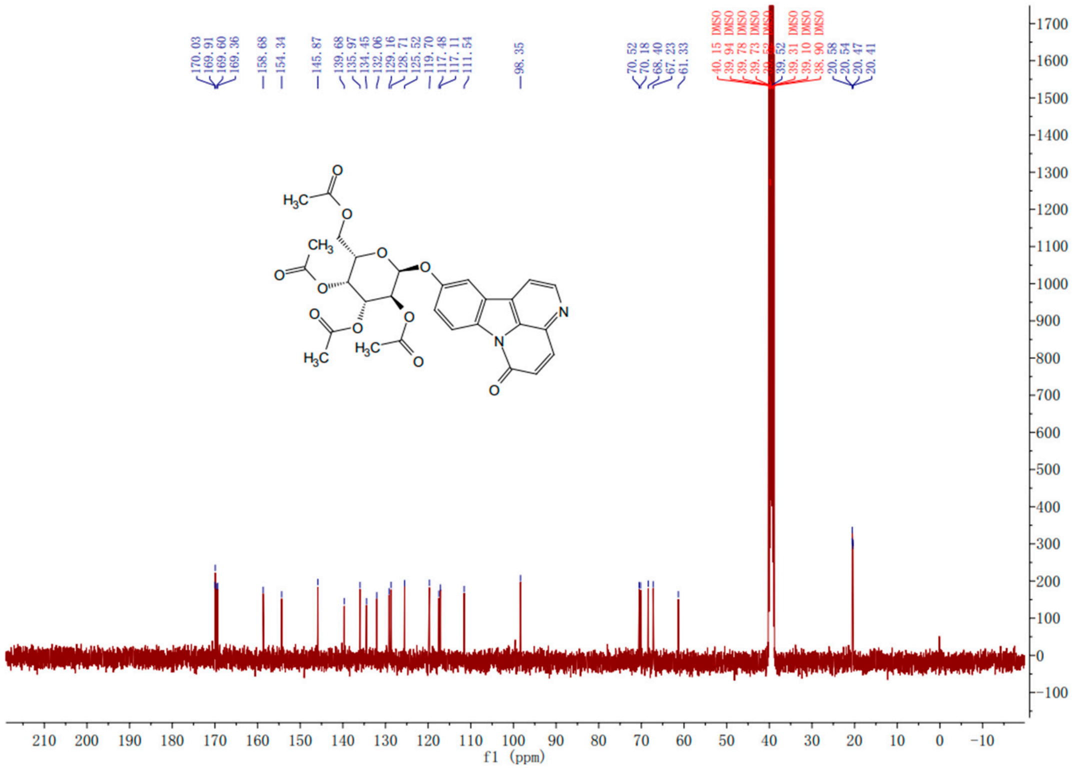

Figure S45: The ^{13}C NMR spectrum of compound **30d** in DMSO-*d6* (100 MHz).

$C_{40}H_{42}N_2O_{19}$ (854.24)

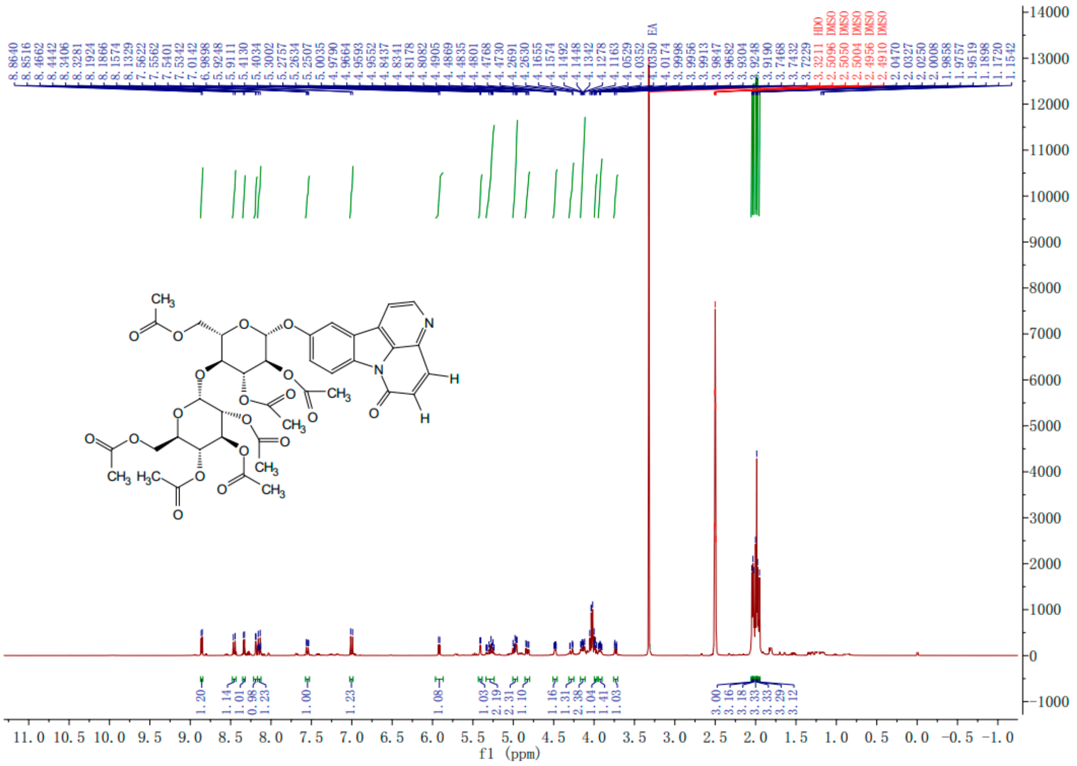

Figure S46: The ^{1}H NMR spectrum of compound **30e** in DMSO-*d6* (400 MHz).

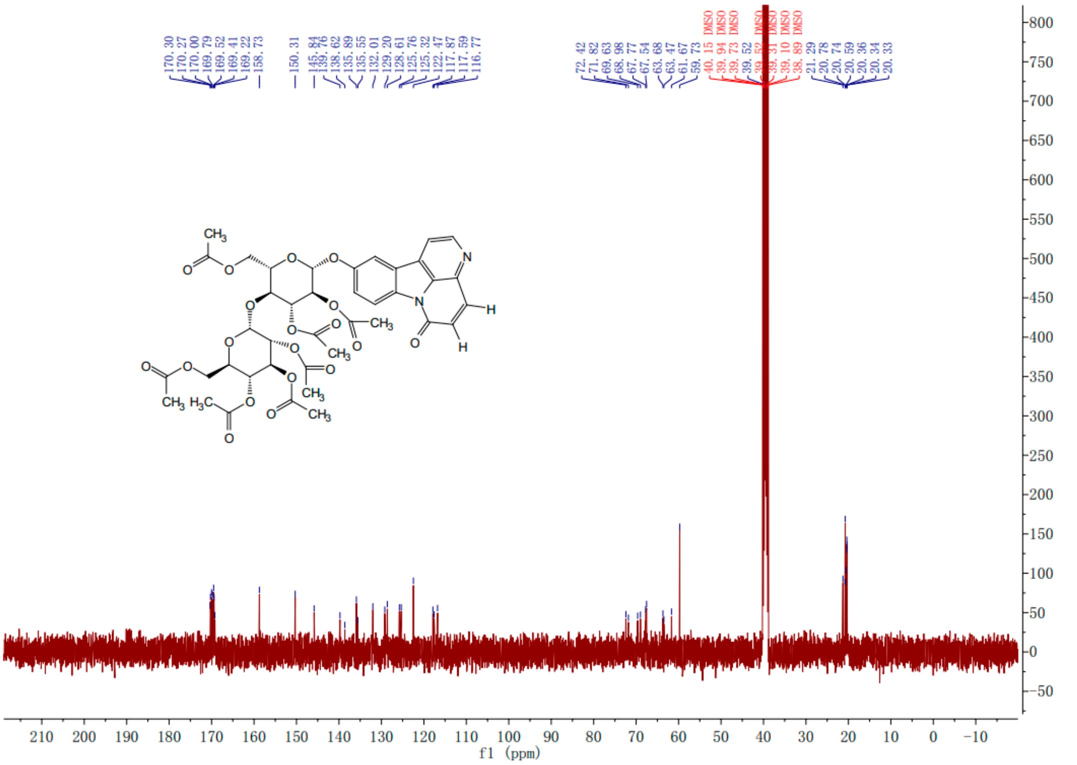

Figure S47: The ^{13}C NMR spectrum of compound **30e** in DMSO-*d6* (100 MHz).

$C_{20}H_{18}N_2O_7$ (398.11)

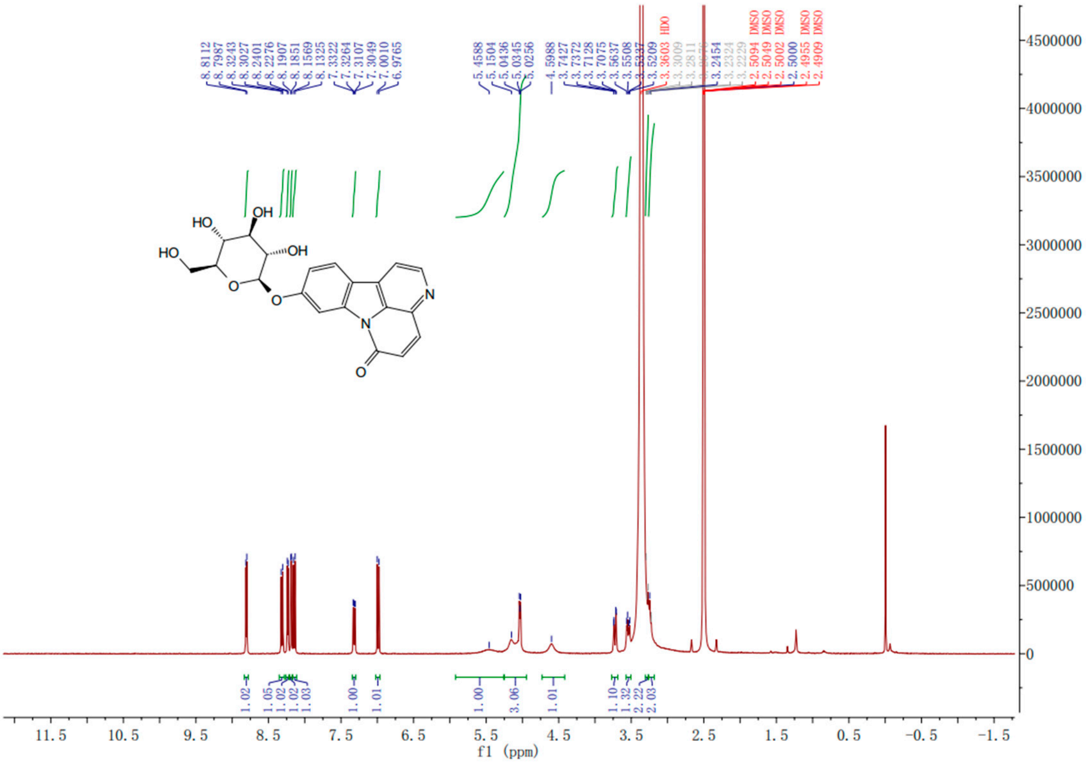

Figure S48: The ^{1}H NMR spectrum of compound **12** in DMSO-*d6* (400 MHz).

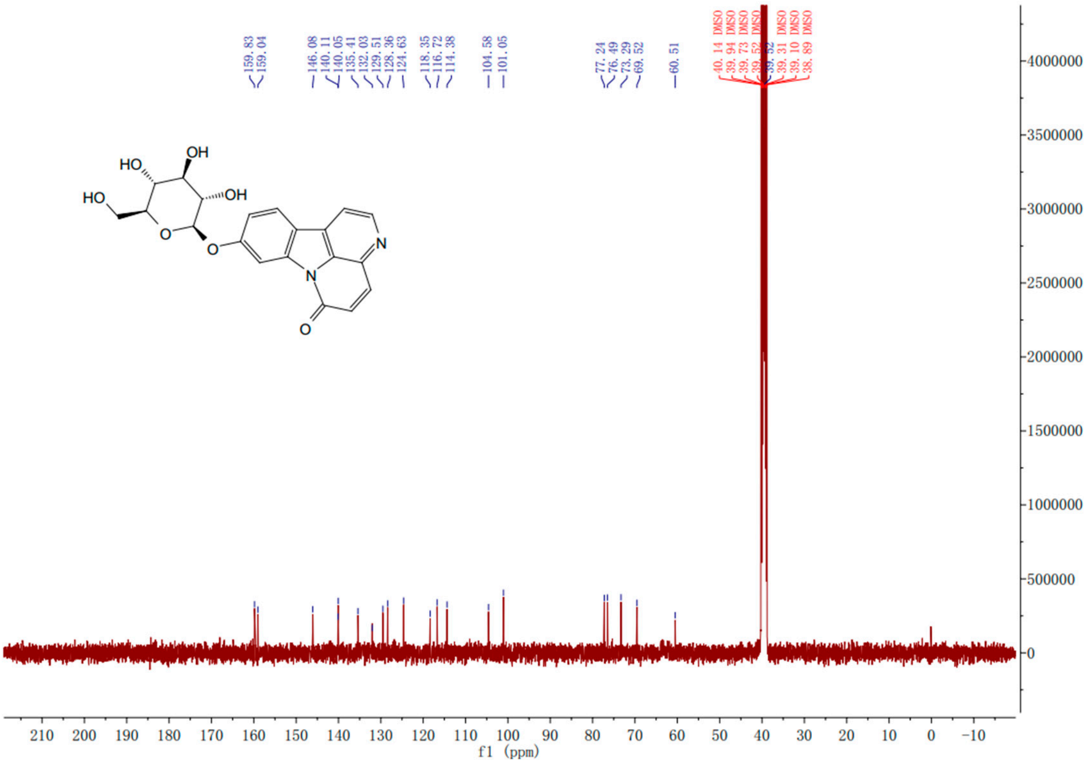

Figure S49: The ^{13}C NMR spectrum of compound **12** in DMSO-*d6* (100 MHz).

$C_{20}H_{18}N_2O_7$ (398.11)

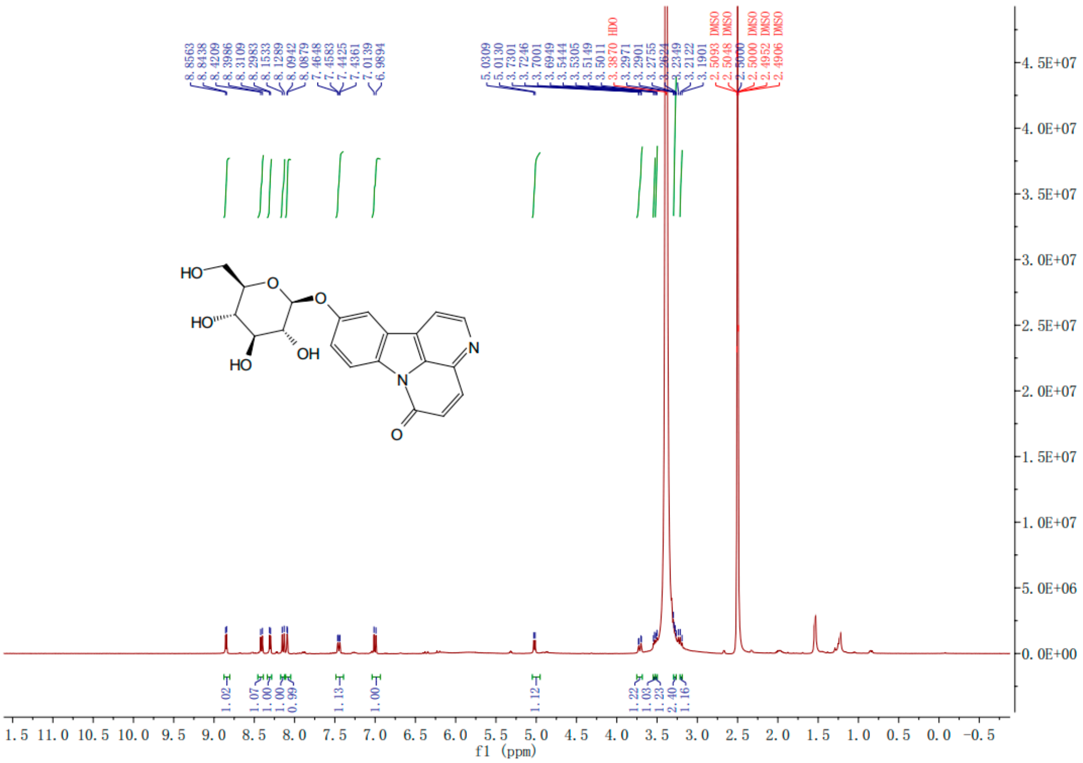

Figure S50: The ^{1}H NMR spectrum of compound **13** in DMSO-*d6* (400 MHz).

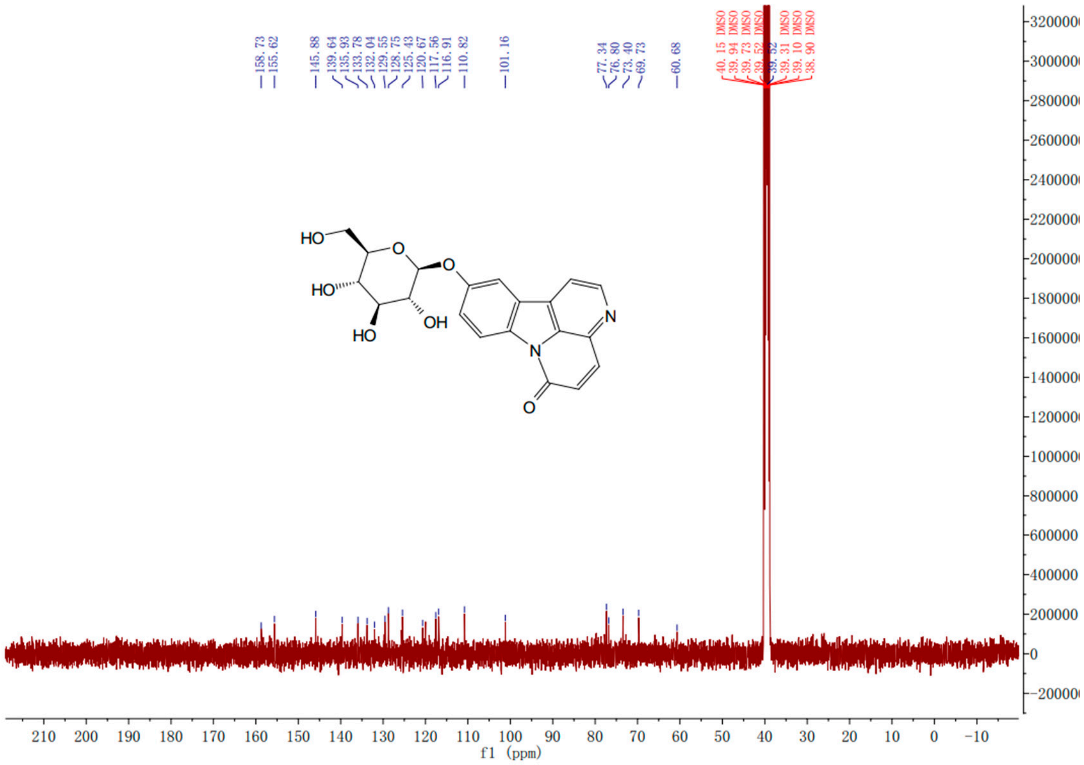

Figure S51: The ^{13}C NMR spectrum of compound **13** in DMSO-*d6* (100 MHz).

$C_{20}H_{18}N_2O_7$ (398.11)

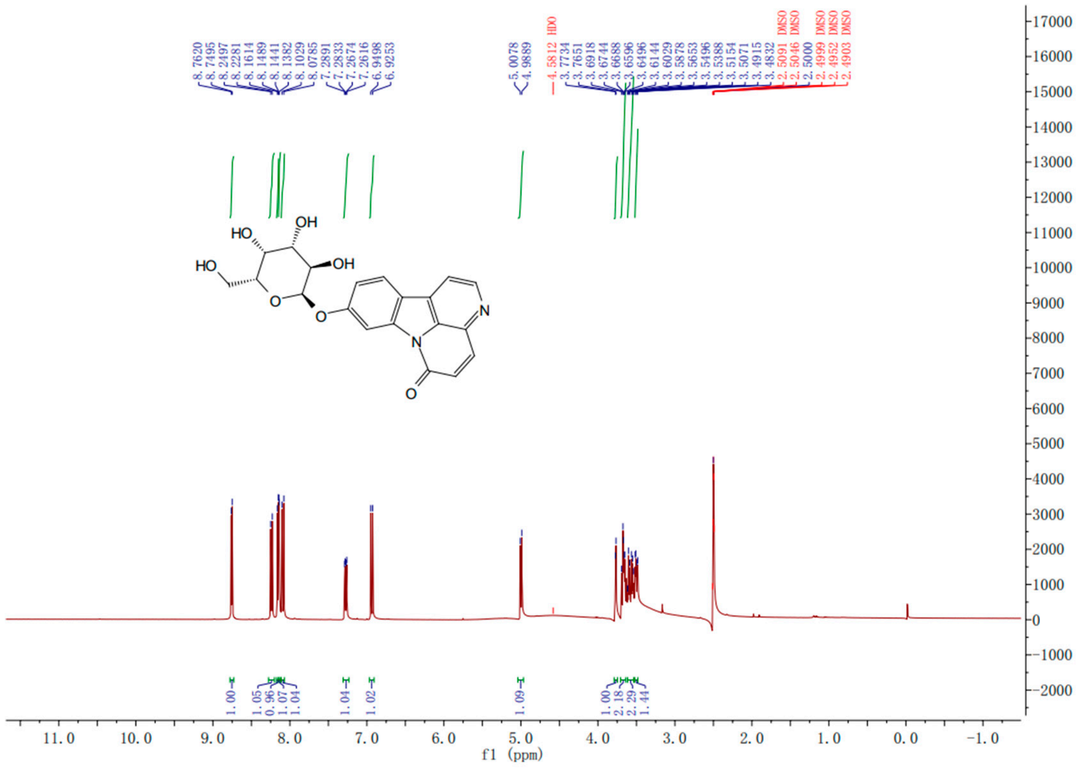

Figure S52: The ^{1}H NMR spectrum of compound **31a** in DMSO-*d6* (400 MHz).

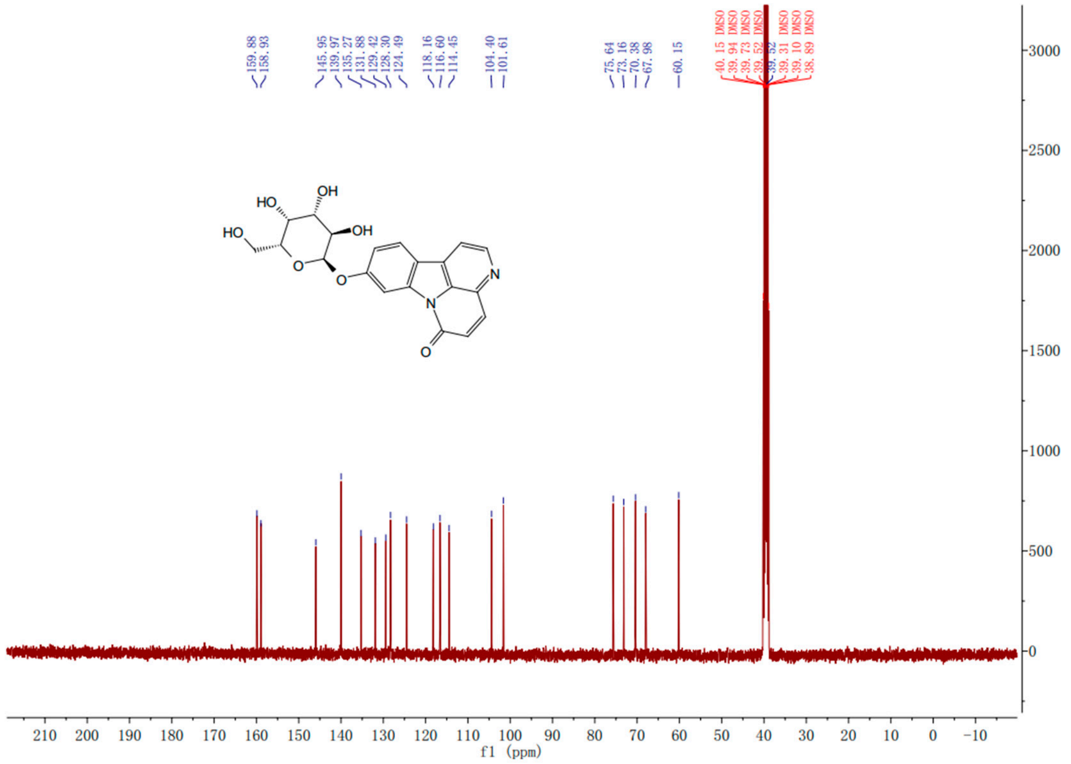

Figure S53: The ^{13}C NMR spectrum of compound **31a** in DMSO-*d6* (100 MHz).

$C_{20}H_{18}N_2O_7$ (398.11)

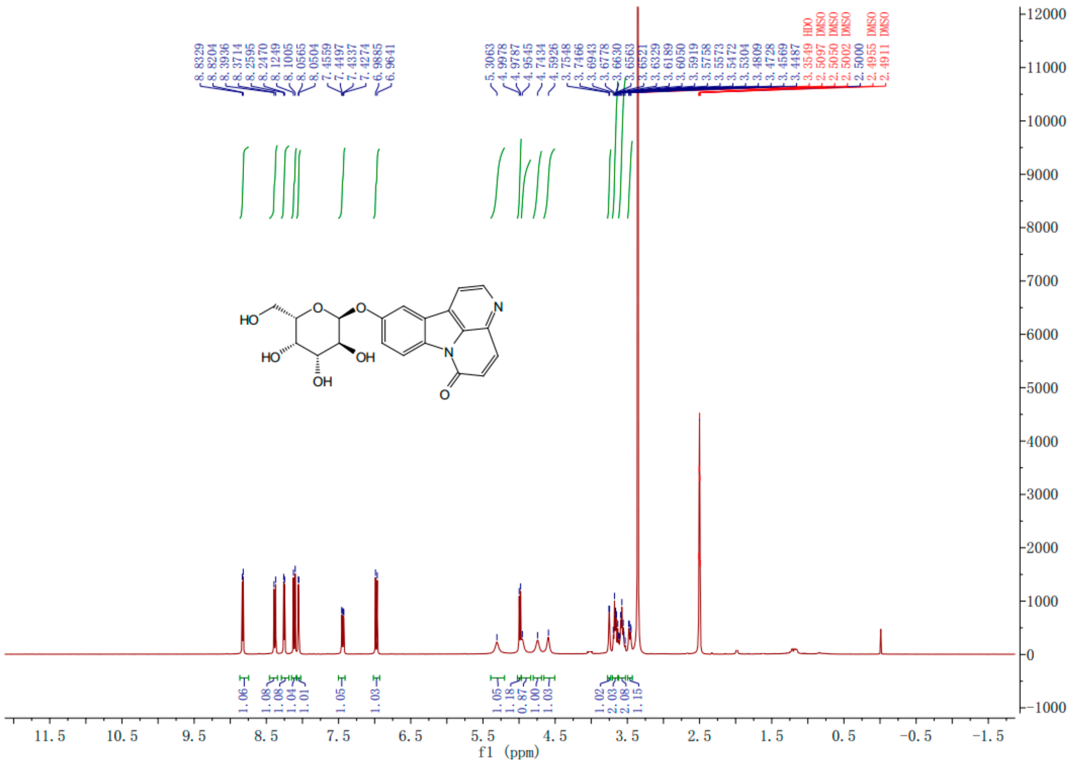

Figure S54: The ^{1}H NMR spectrum of compound **31b** in DMSO-*d6* (400 MHz).

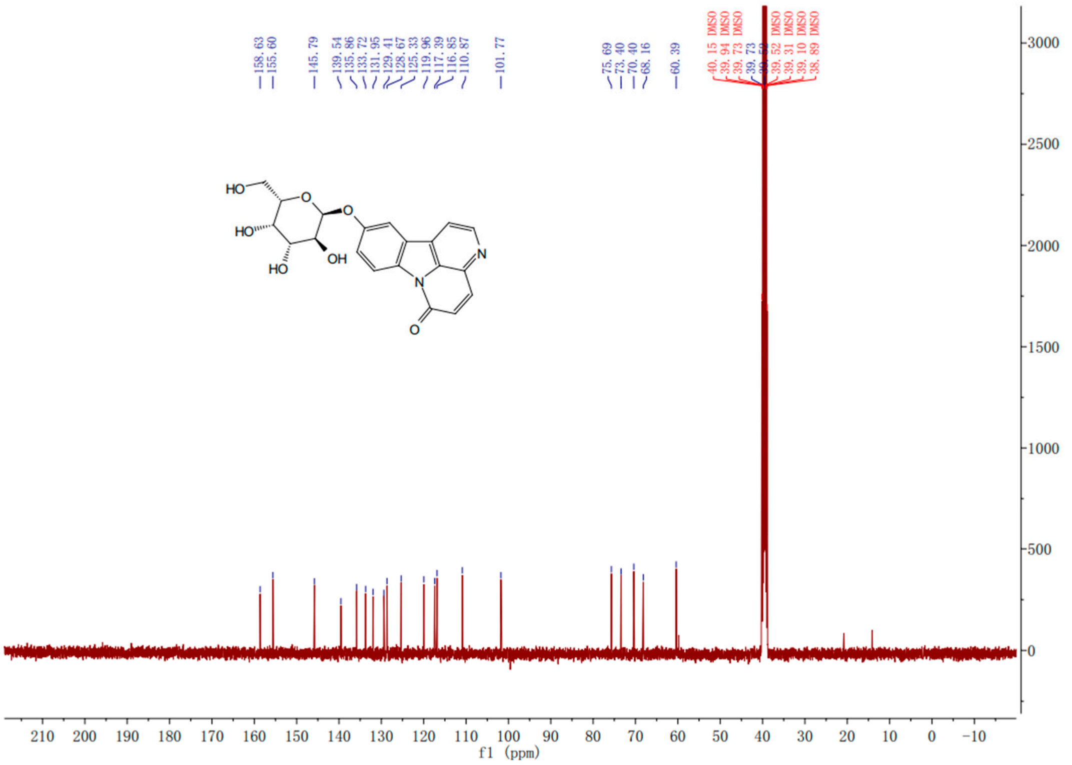

Figure S55: The ^{13}C NMR spectrum of compound **31b** in DMSO-*d6* (100 MHz).

$C_{26}H_{28}N_2O_{12}$ (560.16)

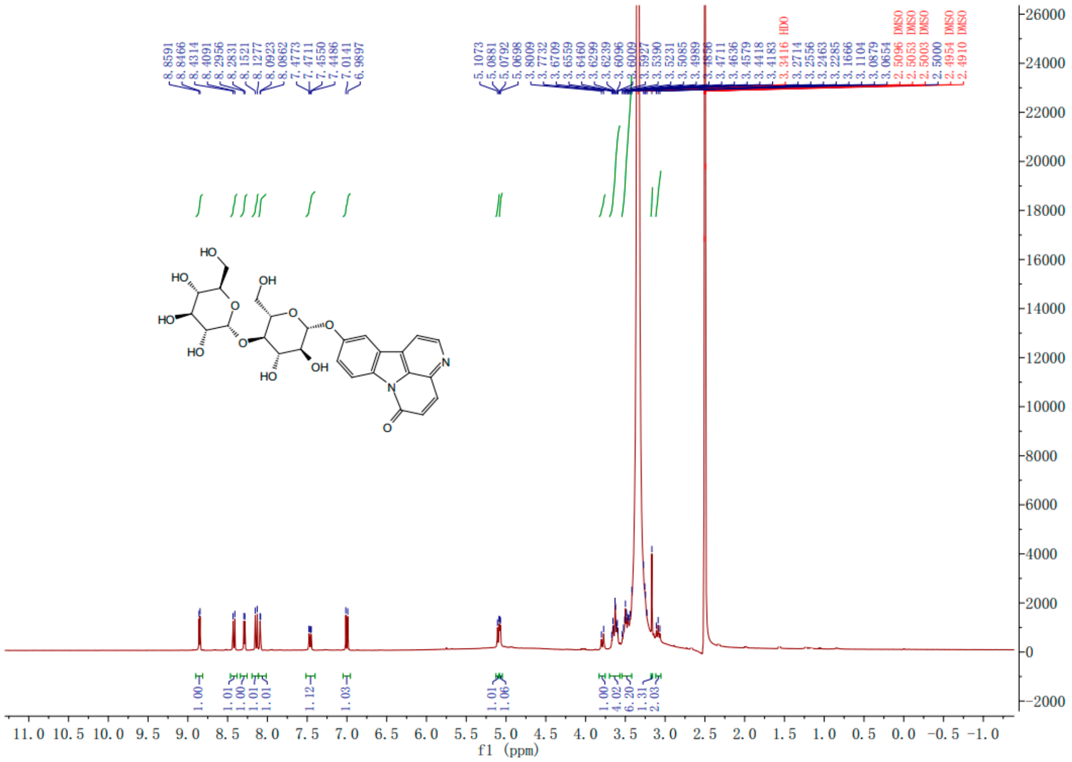

Figure S56: The ^{1}H NMR spectrum of compound **31c** in DMSO-*d6* (400 MHz).

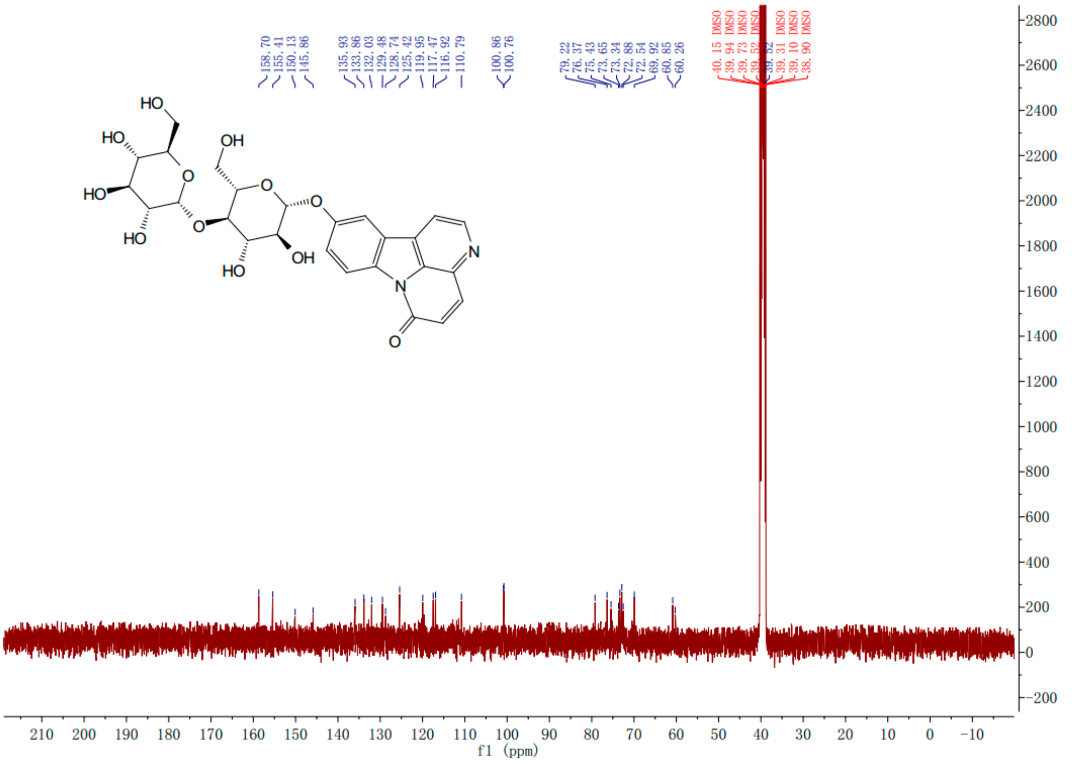

Figure S57: The ^{13}C NMR spectrum of compound **31c** in DMSO-*d6* (100 MHz).

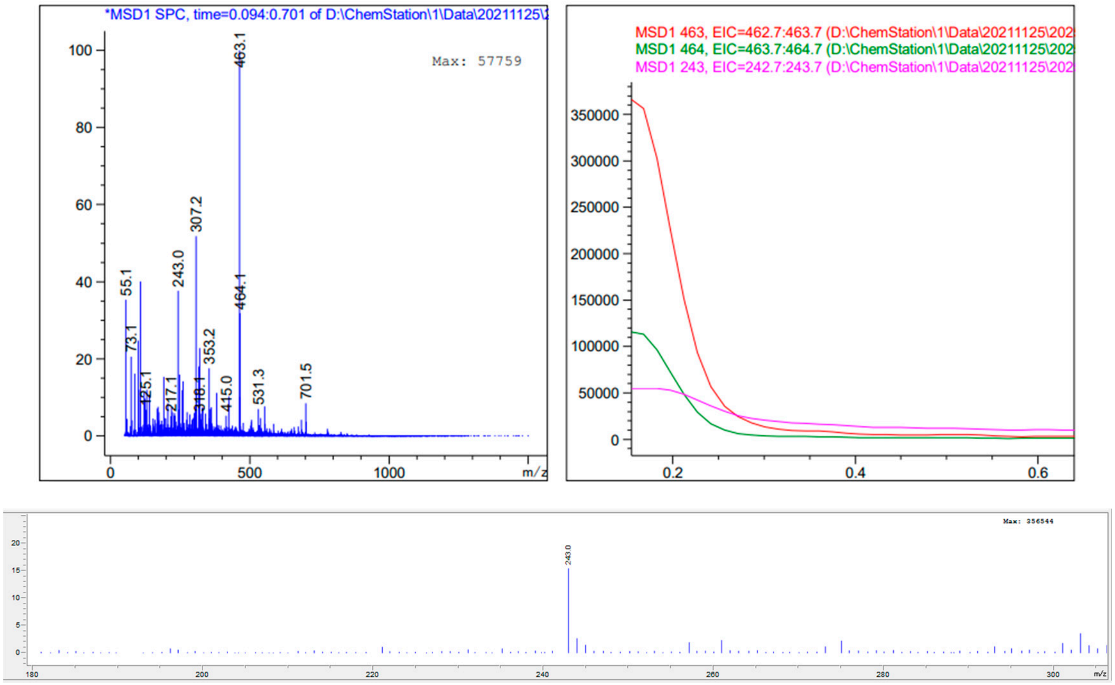

Figure S58: Mass spectra of compound **1**.

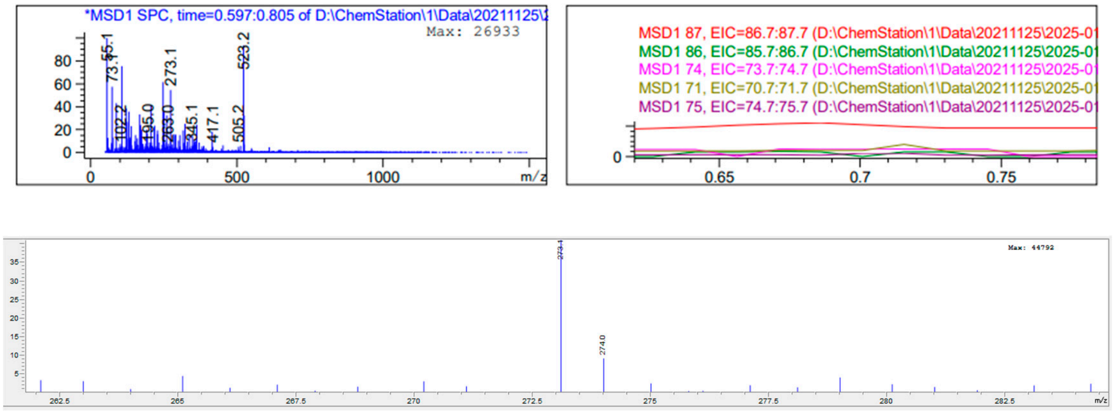

Figure S59: Mass spectra of compound **2**.

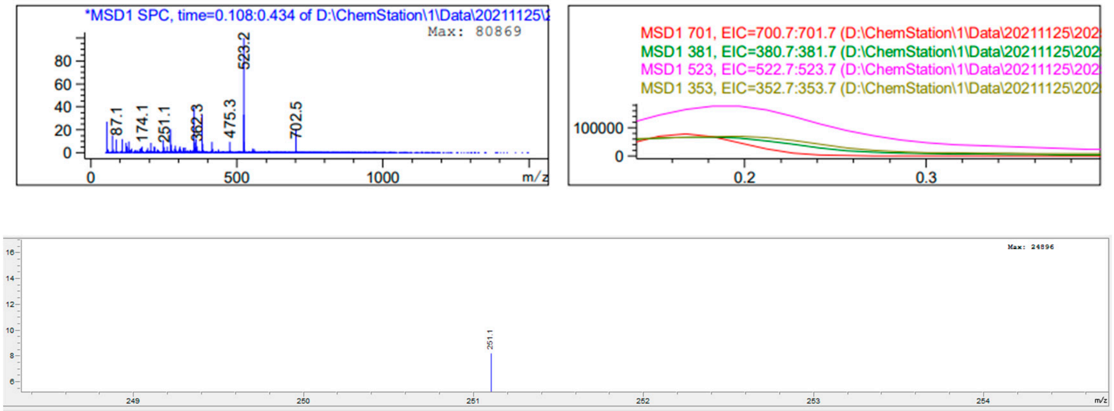

Figure S60: Mass spectra of compound **3**.

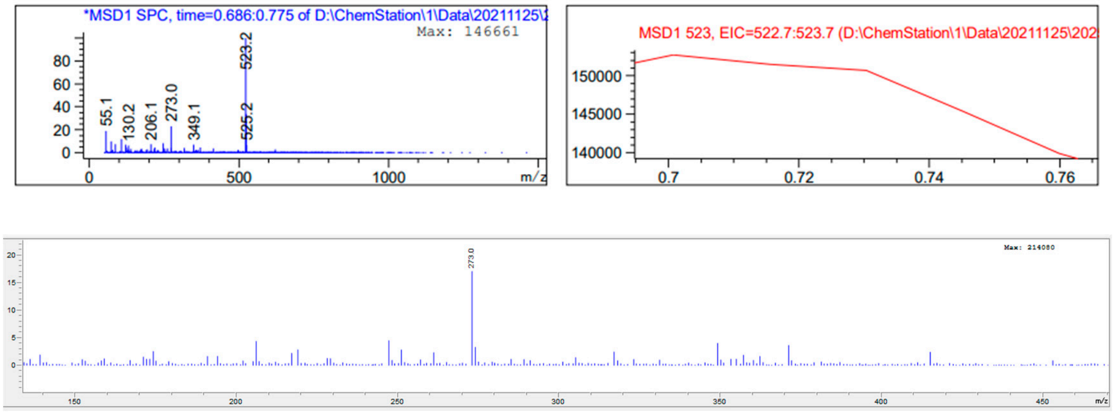

Figure S61: Mass spectra of compound **4**.

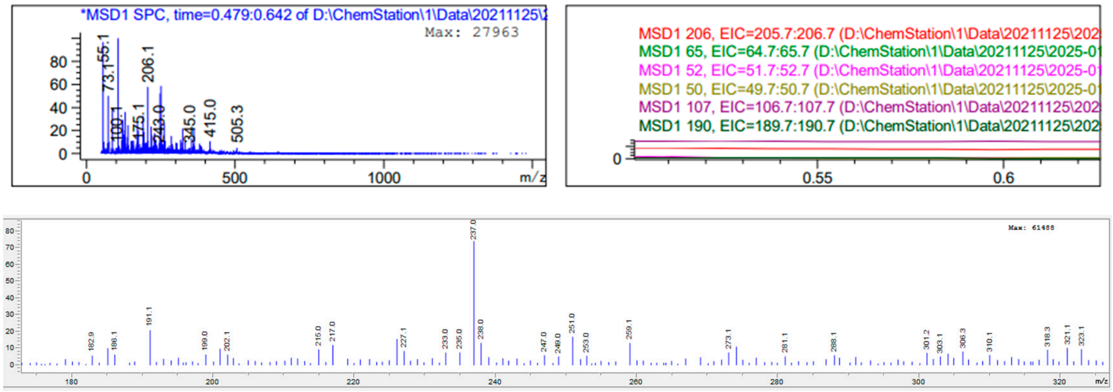

Figure S62: Mass spectra of compound **5**.

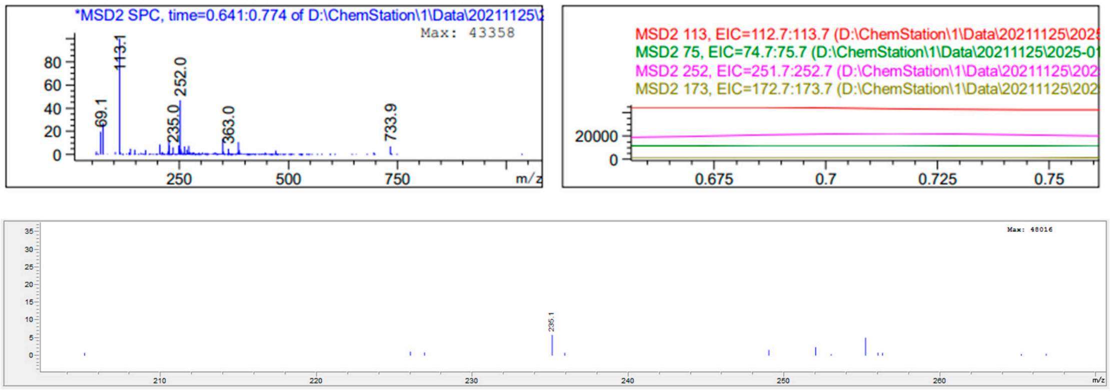

Figure S63: Mass spectra of compound **6**.

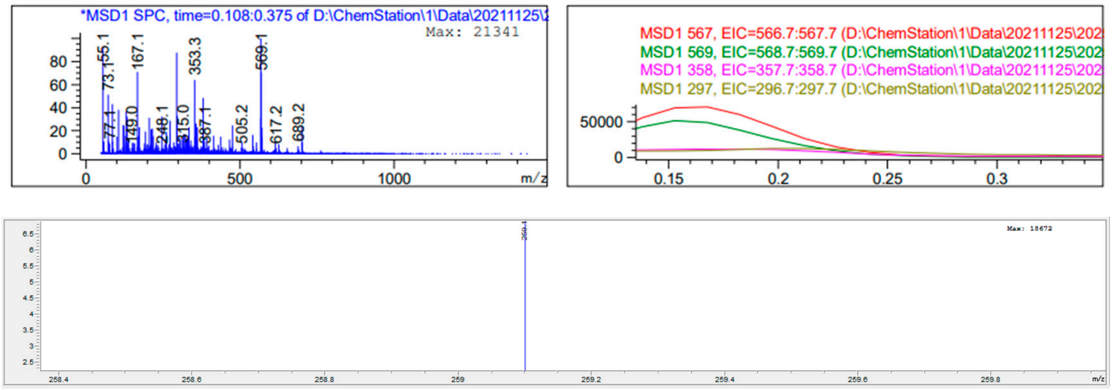

Figure S64: Mass spectra of compound **7**.

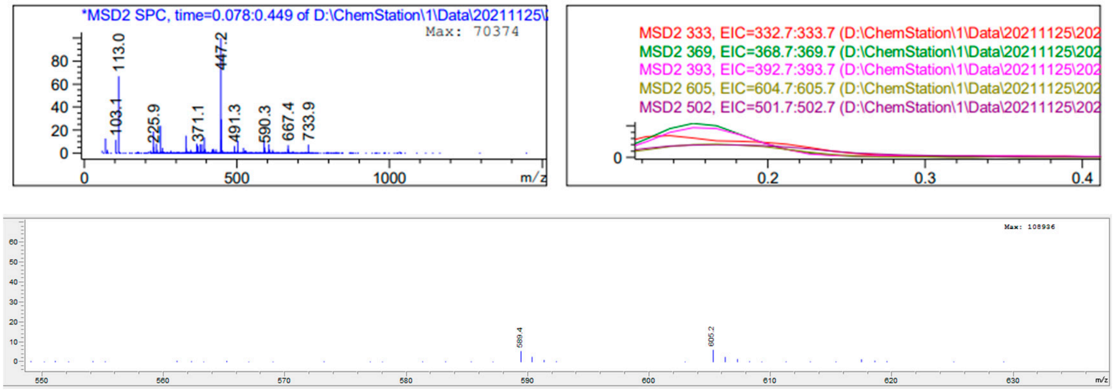

Figure S65: Mass spectra of compound **30a**.

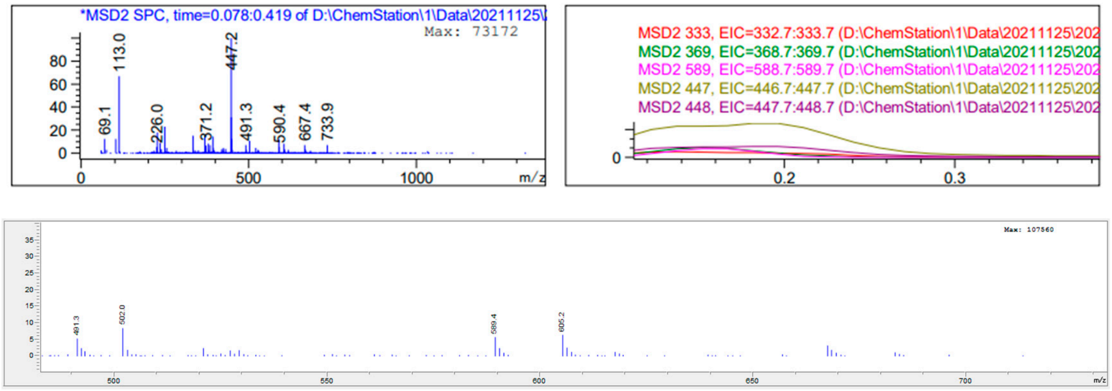

Figure S66: Mass spectra of compound **30b**.

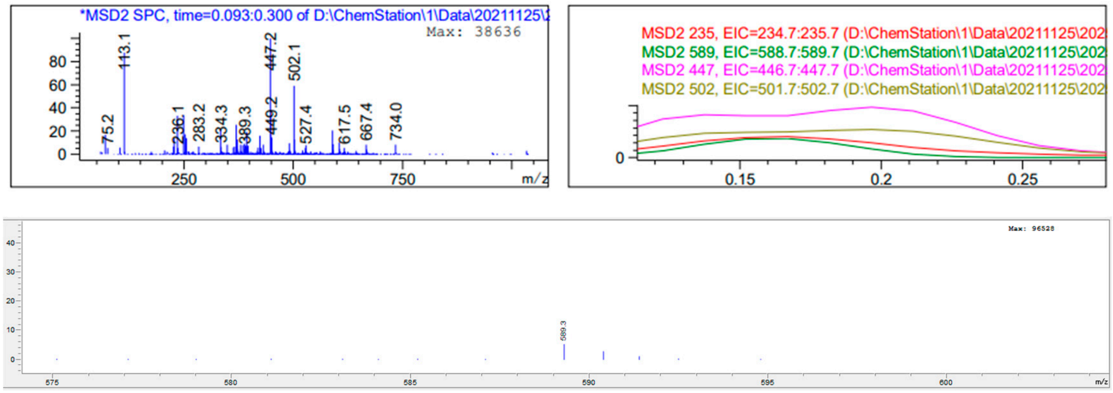

Figure S67: Mass spectra of compound **30c**.

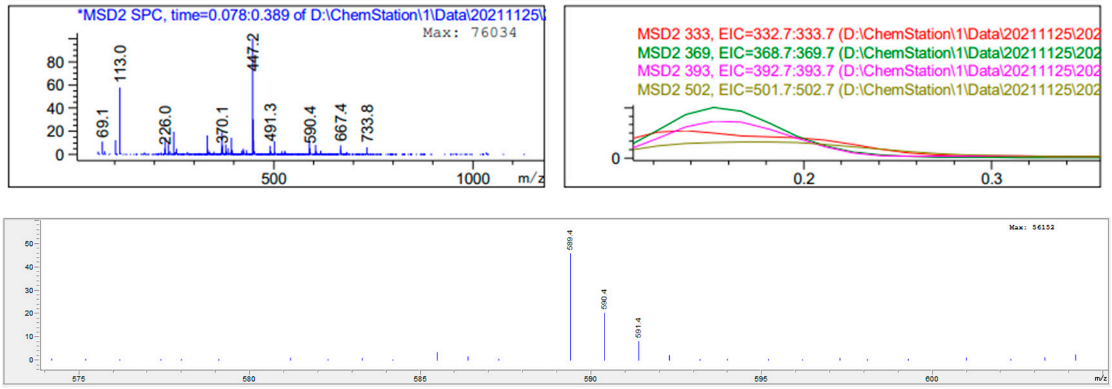

Figure S68: Mass spectra of compound **30d**.

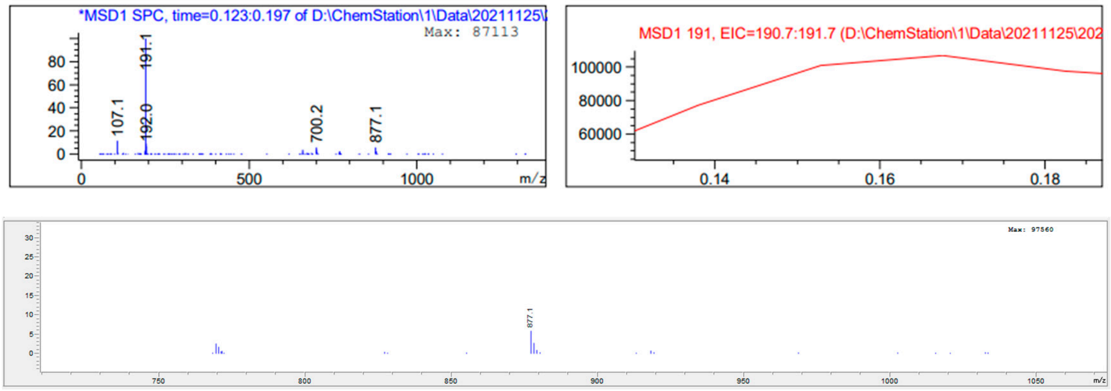

Figure S69: Mass spectra of compound **30e**.

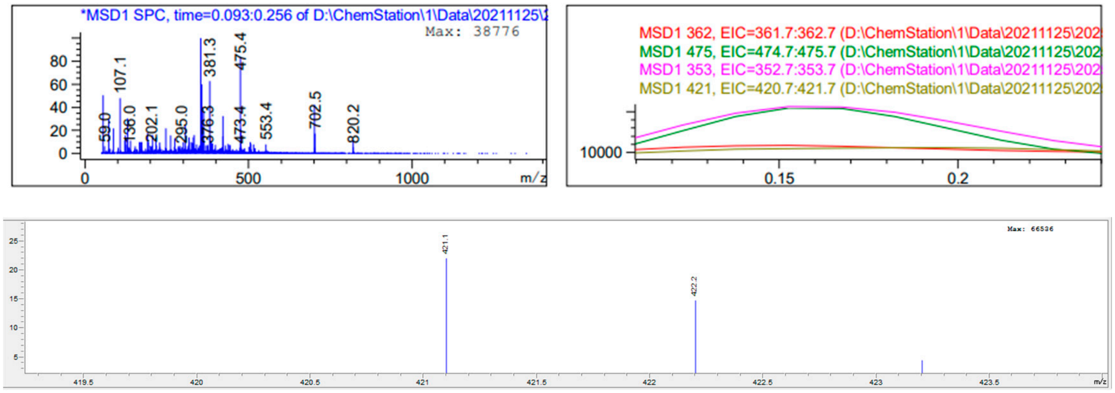

Figure S70: Mass spectra of compound **12**.

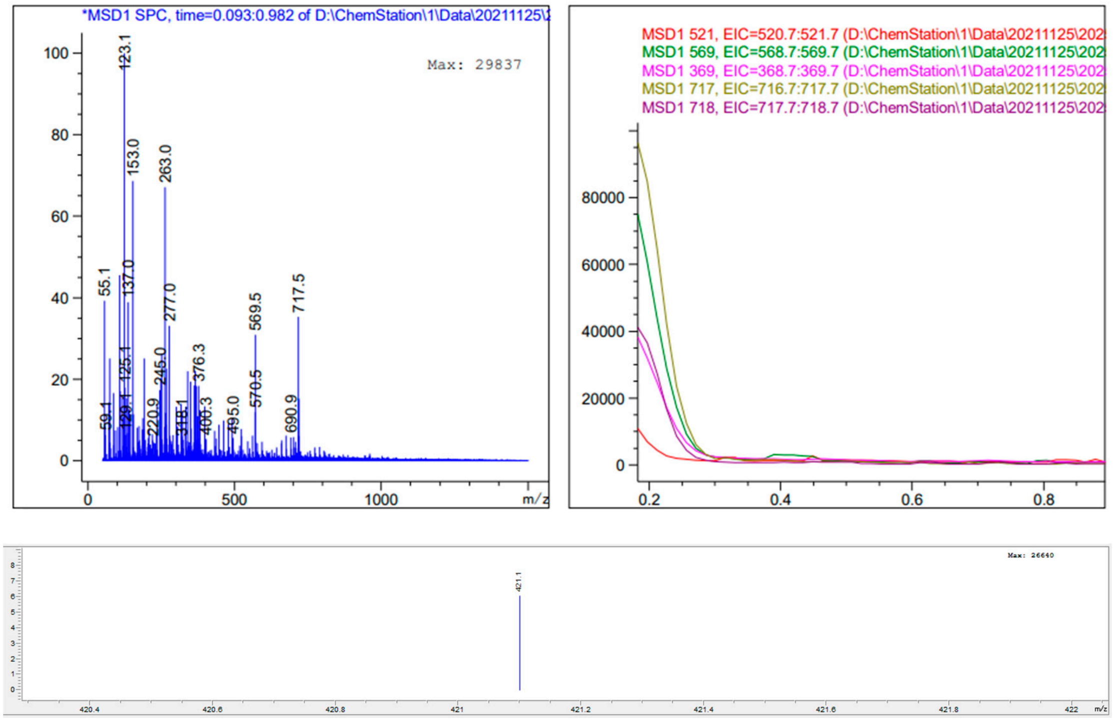

Figure S71: Mass spectra of compound **13**.

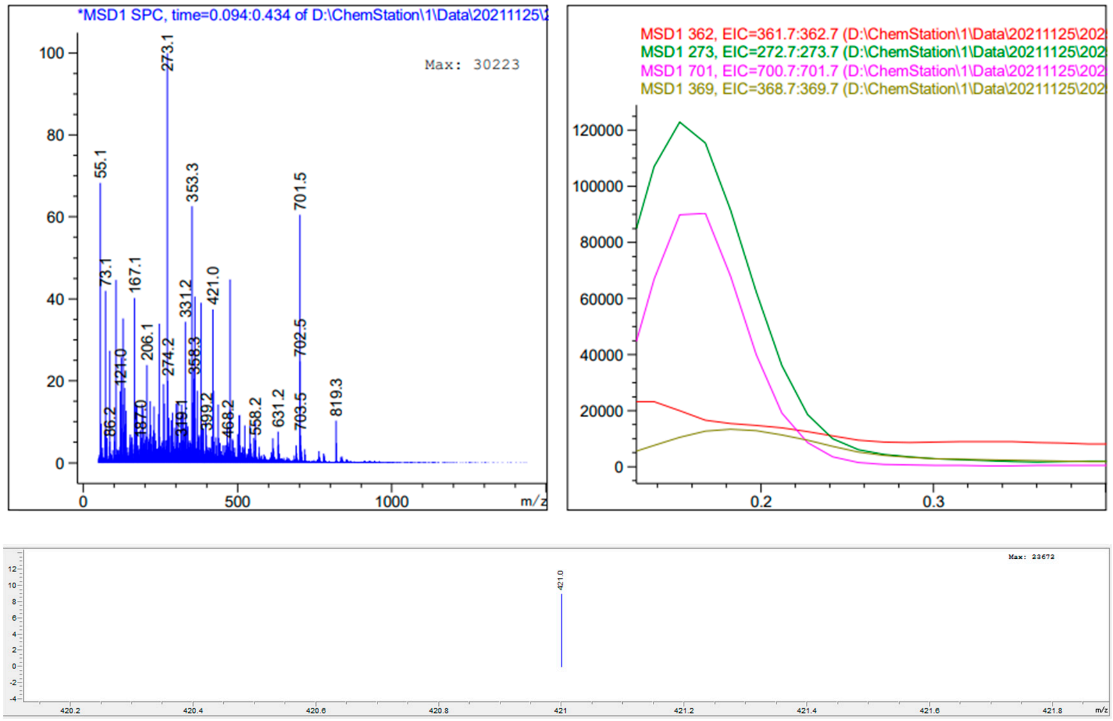

Figure S72: Mass spectra of compound **31a**.

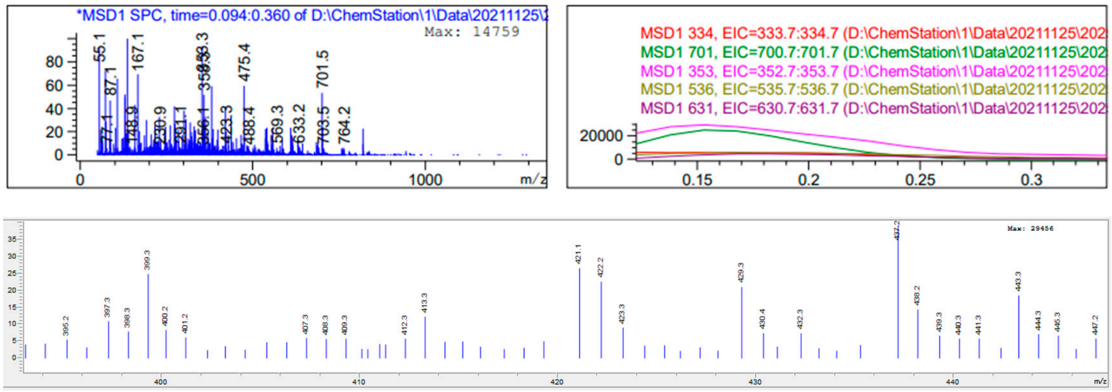

Figure S73: Mass spectra of compound **31b**.

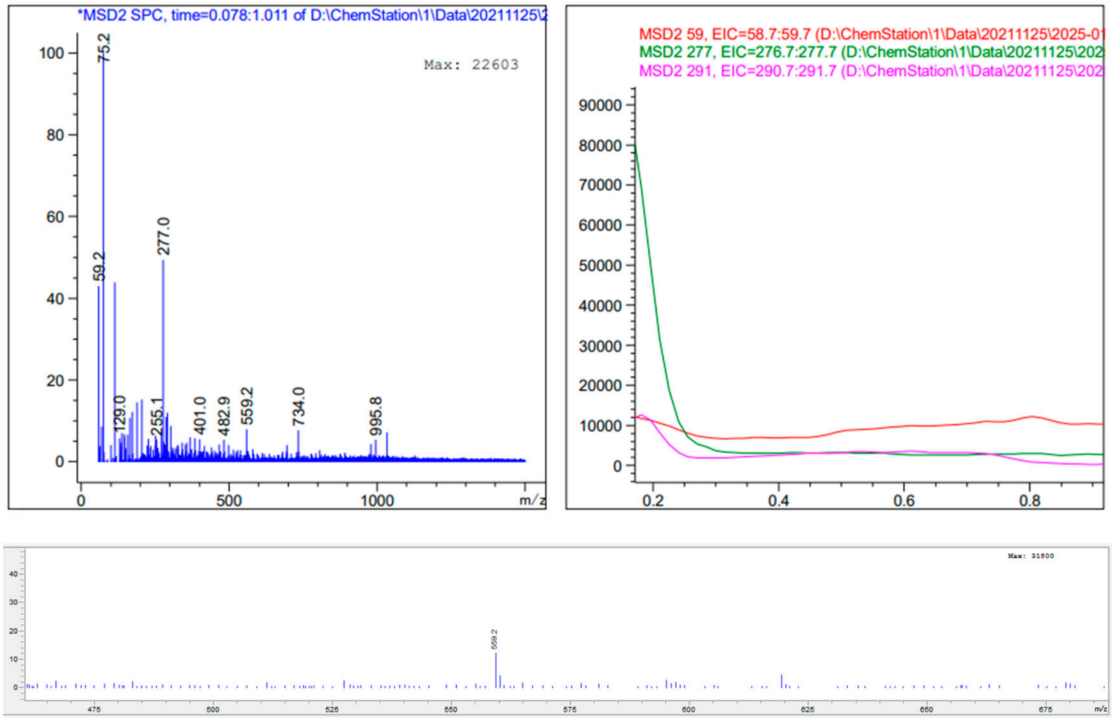

Figure S74: Mass spectra of compound **31c**.
